# Characterization of an *Aplysia* vasotocin signaling system and actions of posttranslational modifications and individual residues of the ligand on receptor activity

**DOI:** 10.3389/fphar.2023.1132066

**Published:** 2023-03-20

**Authors:** Ju-Ping Xu, Xue-Ying Ding, Shi-Qi Guo, Hui-Ying Wang, Wei-Jia Liu, Hui-Min Jiang, Ya-Dong Li, Ping Fu, Ping Chen, Yu-Shuo Mei, Guo Zhang, Hai-Bo Zhou, Jian Jing

**Affiliations:** ^1^ State Key Laboratory of Pharmaceutical Biotechnology, Jiangsu Engineering Research Center for MicroRNA Biology and Biotechnology, Chemistry and Biomedicine Innovation Center, Institute for Brain Sciences, Advanced Institute for Life Sciences, School of Life Sciences, Chinese Academy of Medical Sciences Research Unit of Extracellular RNA, Nanjing University, Nanjing, Jiangsu, China; ^2^ School of Electronic Science and Engineering, Nanjing University, Nanjing, Jiangsu, China; ^3^ Peng Cheng Laboratory, Shenzhen, China; ^4^ Department of Neuroscience and Friedman Brain Institute, Icahn School of Medicine at Mount Sinai, New York, NY, United States

**Keywords:** vasotocin, vasotocin receptor, *Aplysia californica*, IP1 accumulation assay, disulfide bond, C-terminal amidation

## Abstract

The vasopressin/oxytocin signaling system is present in both protostomes and deuterostomes and plays various physiological roles. Although there were reports for both vasopressin-like peptides and receptors in mollusc *Lymnaea* and Octopus, no precursor or receptors have been described in mollusc *Aplysia*. Here, through bioinformatics, molecular and cellular biology, we identified both the precursor and two receptors for *Aplysia* vasopressin-like peptide, which we named *Aplysia* vasotocin (apVT). The precursor provides evidence for the exact sequence of apVT, which is identical to conopressin G from cone snail venom, and contains 9 amino acids, with two cysteines at position 1 and 6, similar to nearly all vasopressin-like peptides. Through inositol monophosphate (IP1) accumulation assay, we demonstrated that two of the three putative receptors we cloned from *Aplysia* cDNA are true receptors for apVT. We named the two receptors as apVTR1 and apVTR2. We then determined the roles of post-translational modifications (PTMs) of apVT, i.e., the disulfide bond between two cysteines and the C-terminal amidation on receptor activity. Both the disulfide bond and amidation were critical for the activation of the two receptors. Cross-activity with conopressin S, annetocin from an annelid, and vertebrate oxytocin showed that although all three ligands can activate both receptors, the potency of these peptides differed depending on their residue variations from apVT. We, therefore, tested the roles of each residue through alanine substitution and found that each substitution could reduce the potency of the peptide analog, and substitution of the residues within the disulfide bond tended to have a larger impact on receptor activity than the substitution of those outside the bond. Moreover, the two receptors had different sensitivities to the PTMs and single residue substitutions. Thus, we have characterized the *Aplysia* vasotocin signaling system and showed how the PTMs and individual residues in the ligand contributed to receptor activity.

## Introduction

**FIGURE 1:**
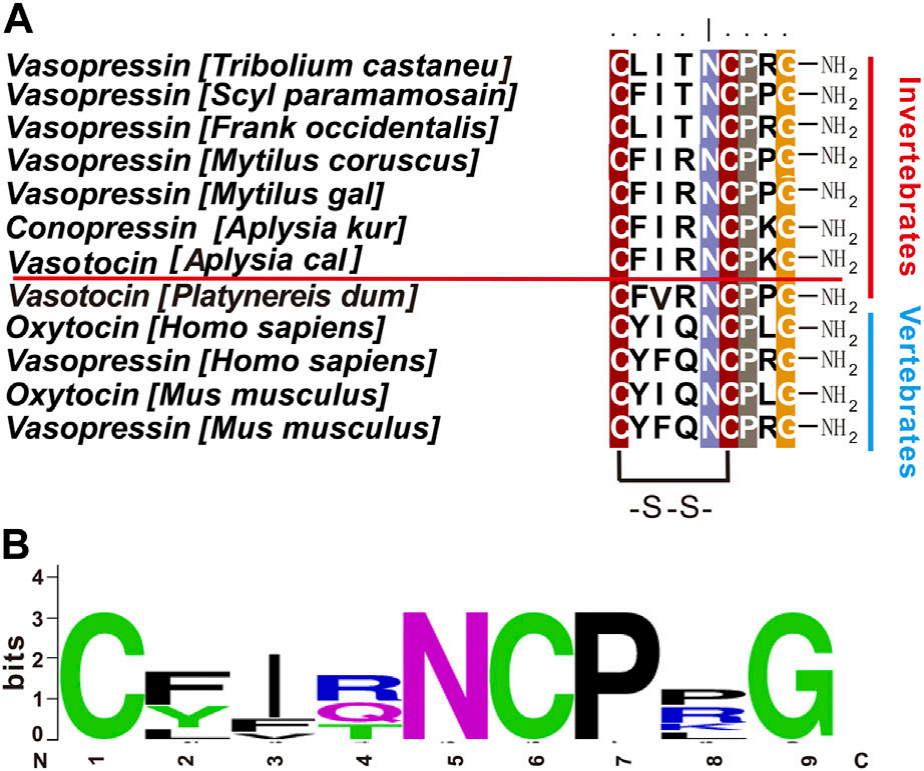
Vasopressin/oxytocin-like peptides in different species. **(A)** Comparison of vasopressin/oxytocin-like peptides in different species using BioEdit (ClustalW Multiple alignments—Graphic View). *Aplysia* vasotocin is underlined. **(B)** A frequency plot for the sequences in **(A)** using Weblogo v2.8.2 (http://weblogo.berkeley.edu/logo.cgi). Other than the two cysteine residues, residues at positions 5, 7, 9 are also highly conserved.

Neuropeptides are the most diverse class of neuromodulators that act on G-protein coupled receptors (GPCRs) to regulate a variety of motivated behaviors (Nassel and Zandawala, 2019; Taghert and Nitabach, 2012; Cropper et al., 2018a; Abid et al., 2021; Nusbaum et al., 2017; Zhang et al., 2022). Among them, vasopressin (VP) and oxytocin (OT) signaling systems are of significant interest (Carter et al., 2020) because they have been shown to play a variety of roles in olfaction (Gravati et al., 2010; Tobin et al., 2010; Knobloch et al., 2012), social interactions (Arakawa et al., 2010; Choleris et al., 2007; Griffin and Flanagan-Cato, 2011), metabolism (Altirriba et al., 2014; Deblon et al., 2011; Le et al., 2011; Mohan et al., 2018), fear conditioning (Huber et al., 2005; Wilson et al., 2005; Cenquizca and Swanson, 2007), learning (Bielsky et al., 2005; Veenema et al., 2010; Curley et al., 2012), and sensory and motor regulation (Breton et al., 2008; Schorscher-Petcu et al., 2010; Wagenaar et al., 2010). In addition, most of the peptide ligands of the vasopressin and oxytocin signaling systems consist of 9 amino acids, with two cysteines at position 1 and 6, and the amidated C terminus, and are highly conserved in both protostomes and deuterostomes (Figure 1) (Elphick et al., 2018). In general, there is only one type of vasopressin/oxytocin-like peptide in most protostomes, whereas there are two, i.e., vasopressin and oxytocin, in deuterostomes. It is widely believed that the separation of the oxytocin and vasopressin genes resulted from the duplication of genes in the common ancestor of the jawless fish 500 million years ago (Elphick et al., 2018).

We sought to study an oxytocin/vasopressin signaling system in the gastropod mollusc *Aplysia californica*. *Aplysia* is an experimentally-advantageous system and has provided fundamental insight into the neural basis of motivated behaviors (Jing and Weiss, 2002; Jing et al., 2004; Sasaki et al., 2009; Jing et al., 2010; Sasaki et al., 2013; Zhang et al., 2020; Bedecarrats et al., 2021; Evans et al., 2021; Due et al., 2022; Wang et al., 2023), learning and memory (Sieling et al., 2014; Byrne and Hawkins, 2015; Orvis et al., 2022) and neuromodulation (Cropper et al., 2018b; Zhang et al., 2022), including neuropeptides (Livnat et al., 2016; Zhang et al., 2017; Do et al., 2018; Zhang et al., 2018; Chan-Andersen et al., 2022) and receptors (Bauknecht and Jekely, 2015; Checco et al., 2018; Guo et al., 2022; Jiang et al., 2022; Zhang et al., 2022). The first evidence for the presence of oxytocin/vasopressin-related neuropeptide in protostomes comes from an early immunohistochemical study (Remy et al., 1979), and the later identification of an arginine vasopressin-like diuretic hormone (Proux et al., 1987), both in insects. In gastropod molluscs, five types of vasopressin/oxytocin homologs have been identified in venoms of different species of *Conus*, two of which have been named as Lys-Conopressin G in *Conus geographus* (CFIRNCPKG-NH2) and Lys-Conopressin S in *Conus striatus* (CIIRNCPRG-NH2) (Cruz et al., 1987; Lewis et al., 2012; Lebbe and Tytgat, 2016), although currently there were no reports of endogenous vasopressin-like peptides in these cone snails. Early studies in *Aplysia* have shown that endogenous oxytocin/vasopressin-related substances are present in this species (Moore et al., 1981), and based on mass, its sequence appears to be consistent with Lys-conopressin G of *Conus* (Moore et al., 1981; Thornhill et al., 1981). Immunohistochemical studies of the *Aplysia* central nervous system suggested that VP-like immunoreactivity is restricted to a single neuron in the abdominal ganglion and two small neurons located bilaterally in each pedal ganglion (Martinez-Padron et al., 1992). VP/OT-type neuropeptides decrease the spiking frequency of the gill motor neuron L7 in the abdominal ganglion and accordingly inhibit the gill-withdrawal reflex (Thornhill et al., 1981). It is also reported that VP/OT-type neuropeptides increase the spiking frequency of the abdominal R15 neuron (Lukowiak et al., 1980).

Despite the progress described above, the exact sequence of *Aplysia* conopressin remains to be determined, and neither the precursor nor the receptors have been described in *Aplysia*. Previous work has identified the precursor (Van Kesteren et al., 1995a) and one vasopressin-like receptor in gastropod mollusc *Lymnaea* (van Kesteren et al., 1995b). Later work identified one additional receptor in *Lymnaea* (van Kesteren et al., 1996). In cephalopod mollusc, octopus, it has been shown to have two members of vasopressin/oxytocin peptides derived from two different precursors, and three corresponding receptors (Kanda et al., 2003; Takuwa-Kuroda et al., 2003). Previous work (Tessmar-Raible et al., 2007; Bauknecht and Jekely, 2015; Williams et al., 2017) has also shown that there are two receptors for a vasopressin-like peptide in annelid *Platynereis dumerilii*, which together with molluscs, belong to superphylum: lophotrochozoa. Thus, there may be two or more receptors in *Aplysia*. Here, we first cloned the precursor for *Aplysia* vasopressin, which provided direct evidence for its exact sequence. Although the sequence is identical to conopressin G, we chose to name the peptide *Aplysia* vasotocin (apVT) instead of conopressin G because conopressin G is only present in the venom of cone snails. This naming convention has been adopted previously in *P. dumerilii* (Bauknecht and Jekely, 2015). We then identified two receptors for apVT, i.e., apVTR1 and apVTR2. We also explored the roles of each residue in apVT by single residue alanine substitution, as well as post-translational modifications (PTMs), i.e., the disulfide bond and C-terminal amidation, to the activation of the two receptors. Our results indicate that the disulfide bond, C-terminal amidation, and most residues are important for the activation of the receptors. Moreover, the two receptors might have different sensitivities to the PTMs and single residue substitution. Thus, we have characterized the *Aplysia* vasotocin signaling system and provided an important basis for the study of its physiological roles.

## Materials and methods

### Subjects and reagents

Experiments were performed on *A. californica* (100–350 g) obtained from Marinus, California, United States. *Aplysia* are hermaphroditic (i.e., each animal has reproductive organs normally associated with both male and female sexes). Animals were maintained in circulating artificial seawater at 14°C–16°C and the animal room was equipped with a 24 h light cycle with the light period from 6:00 a.m. to 6:00 p.m. All chemicals were purchased from Sigma-Aldrich unless otherwise stated.

### Bioinformatic analysis of peptide precursors and receptors

We first used NCBI to find specific sequences of interests. In addition, we also searched AplysiaTools databases (Dr. Thomas Abrams, University of Maryland, United States) to obtain additional sequences for comparison. These latter databases (http://aplysiatools.org) include databases for the *Aplysia* transcriptome and *Aplysia* genome.

The open reading frames (ORFs) from the full-length cDNA sequences of the apVT precursor and putative receptors were obtained using ORF Finder (https://www.ncbi.nlm.nih.gov/orffinder/). For the apVT precursor, the putative signal peptide was predicted using SignalP-5.0 (http://www.cbs.dtu.dk/services/SignalP/) and the putative peptides encoded by the apVT precursor were predicted using NeuroPred (http://stagbeetle.animal.uiuc.edu/cgi-bin/neuropred.py). We also compared the apVT with those of other species using BioEdit software and generated a frequency plot of each amino acid (aligned from the c-terminus) using Weblogo software (http://weblogo.berkeley.edu/logo.cgi). For the putative apVT receptors, transmembrane domains were predicted using TMHMM Server v. 2.0 (http://www.cbs.dtu.dk/services/TMHMM/). For proteins that were difficult to annotate using blast, we also used the Pfam database (http://pfam.xfam.org/search#tabview=tab1) to determine what type of protein it is. The phylogenetic trees of sequences from different species were constructed by MEGA X software (https://www.megasoftware.net/) using the maximum likelihood method with 1,000 replicates. For Figure 4B, we used the “Parathyroid hormone peptide receptor_C.gigas” as an out-group, and LG + G + F model to generate our final tree; for Figure 6, the “RYamide Receptor *Drosophila melanogaster*” was used as an out-group, and LG + F + G + I model was performed which was different from Figure 4B. The selection of the models was based on the results of an initial MEGA analysis.

### Cloning of mRNA in *Aplysia*

#### RNA extraction

After anesthesia with 30%–50% of the body weight with 333 mM MgCl_2_, *Aplysia* cerebral, pleural-pedal, buccal, and abdominal ganglia were dissected out and maintained in artificial seawater containing the following (in mM): 460 NaCl, 10 KCl, 55 MgCl_2_, 11 CaCl_2_, and 10 HEPES buffer, pH 7.6, in a dish lined with Sylgard (Dow Corning). RNA was prepared from the *Aplysia* ganglia using the TRIzol reagent method. Specifically, the dissected ganglia were placed into 200 μL TRIzol (Sigma, T9424) and stored at −80°C until use. The frozen ganglia in TRIzol were thawed and homogenized with a plastic pestle, then TRIzol was added to a total volume of 1 mL, which were incubated at room temperature for 10 min. Then, 200 μL chloroform was added, and the solution was mixed thoroughly by a shaker, and let stand on ice for 15 min. The solution was centrifuged (12,000 × *g*, 4°C, 15 min), and the supernatant was added to an equal volume of isopropanol. The tube was shaken gently by hand and let stand at −20°C for 2 h. After 2 h, it was centrifuged (12,000 × *g*, 4°C, 15 min) again, the supernatant was discarded, 1 mL of 75% ethanol/water was added, and the centrifuge tube was shaken gently by hand to suspend the pellet. It was centrifuged (12,000 × *g*, 4°C, 10 min), the supernatant discarded and the precipitant was dried at room temperature for 5–10 min. Finally, 30 μL of nuclease-free water was added to dissolve the RNA pellet, and the RNA concentration was determined with a Nanodrop ND-1000 spectrophotometer (Thermo Fisher Scientific).

#### Reverse transcription

Using the above-extracted RNA as a template, cDNA was synthesized by reverse transcription using PrimeScript RT Master Mix Kit (Takara, RR036A) according to the instructions and then stored at −20°C until use. The synthesized first-strand cDNA serves as a template for subsequent PCR.

#### PCR

The synthesized cDNA above was used as a template for PCR. Each pair of specific primers was designed (Supplementary Table S1) in Primer Premier 6 and Oligo7, based on protein-coding sequences for the apVT precursor and putative receptors. The PCR reaction was performed with 98°C/2 min pre-denaturing; 98°C/10 s denaturing; ∼60°C (depending on the specific primers: see Supplementary Table S1)/15-s annealing; 72°C/30 s extension and 72°C/5 min re-extension for 35 cycles. The PCR products were subcloned into vector pcDNA3.1(+) and sequenced to ensure that the sequence was correct.

### IP1 accumulation assay

Inositol monophosphate (IP1) accumulation assay measures the concentration of IP1, which is hydrolyzed from the second messenger, inositol triphosphate (IP3). IP3 is generated by Gαq pathway when a G-protein coupled receptor (GPCR) expressed in CHO-K1 cells is activated by an appropriate ligand. To express the *Aplysia* putative receptors transiently in CHO-K1, the cDNA was cloned into the mammalian expression vector pcDNA3.1(+). CHO-K1 cells (Procell, CL-0062) were cultured in F-12K medium (Gibco, 21,127–022) with 10% fetal bovine serum (Genial, G11-70500) at 37°C in 5% CO_2_. Transfection experiments were performed when the cells were grown to 70%–90% confluence. In preliminary experiments, for each dish (60-mm diameter), 3 μg of the putative receptor plasmids [in pcDNA3.1(+)] and 3 μg of the promiscuous Gαq plasmids (also known as Gα16) (Bauknecht and Jekely, 2015; Sharma and Checco, 2021) [in pcDNA3.1(+)] were co-transfected in CHO-K1 cells, mixed with 400 μL of Opti-MEM (Gibco, 11,058,021), followed by the addition of 15 μL of Turbofect (Thermo Fisher Scientific, R0531). Note that the inclusion of Gα16 will ensure a response no matter what signaling pathway (endogenous or not) a putative receptor might couple to. For the Class-A GPCR 3, we could not obtain an IP1 response compared with the apVTR1 and apVTR2, suggesting that this receptor is not a receptor for apVT. Then, for apVTR1 and apVTR2, in each dish (60-mm diameter), 4 μg of plasmids [in pcDNA3.1(+)] was transfected in CHO-K1 cells, mixed with 400 μL of Opti-MEM (Gibco, 11,058,021), followed by the addition of 15 μL of Turbofect (Thermo Fisher Scientific, R0531). Under this condition, we could still obtain an IP1 response, suggesting that apVTR1 and apVTR2 are the receptors of apVT and can associate with the native Gαq in the CHO cells. Thus, for all subsequent IP1 accumulation assays, 4 μg of the apVTR1 or apVTR2 plasmids [in pcDNA3.1(+)] without the promiscuous Gαq plasmids was transfected.

The CHO cells with the reagents added above were mixed gently and incubated at room temperature for 15 min. The DNA/Turbofect mixture dropwise was then added to the dish, and the cells were incubated at 37°C in 5% CO_2_ overnight. The next day, the cells were trypsinized and reseeded in 384-well tissue culture-treated plates (Corning, 3,570) at a density of 20,000 cells/well in F-12K and 10% FBS and incubated at 37°C in 5% CO_2_ overnight. On the third day, the activation of the putative receptor was detected by monitoring IP1 accumulation using an IP1 detection kit (Cisbio, 62IPAPEB) in Tecan Spark. Except for using 0.5x reagent, all other procedures were performed by following the IP1 detection kit manufacturer’s instructions. Peptides are synthesized by Guoping Pharmaceutical (Supplementary Figure S1) and are aliquoted in 50 nmol EP tubes, and stored at −20°C until use.

## Results

### Identifying the precursor for *Aplysia* vasotocin and predicting peptides

**FIGURE 2:**
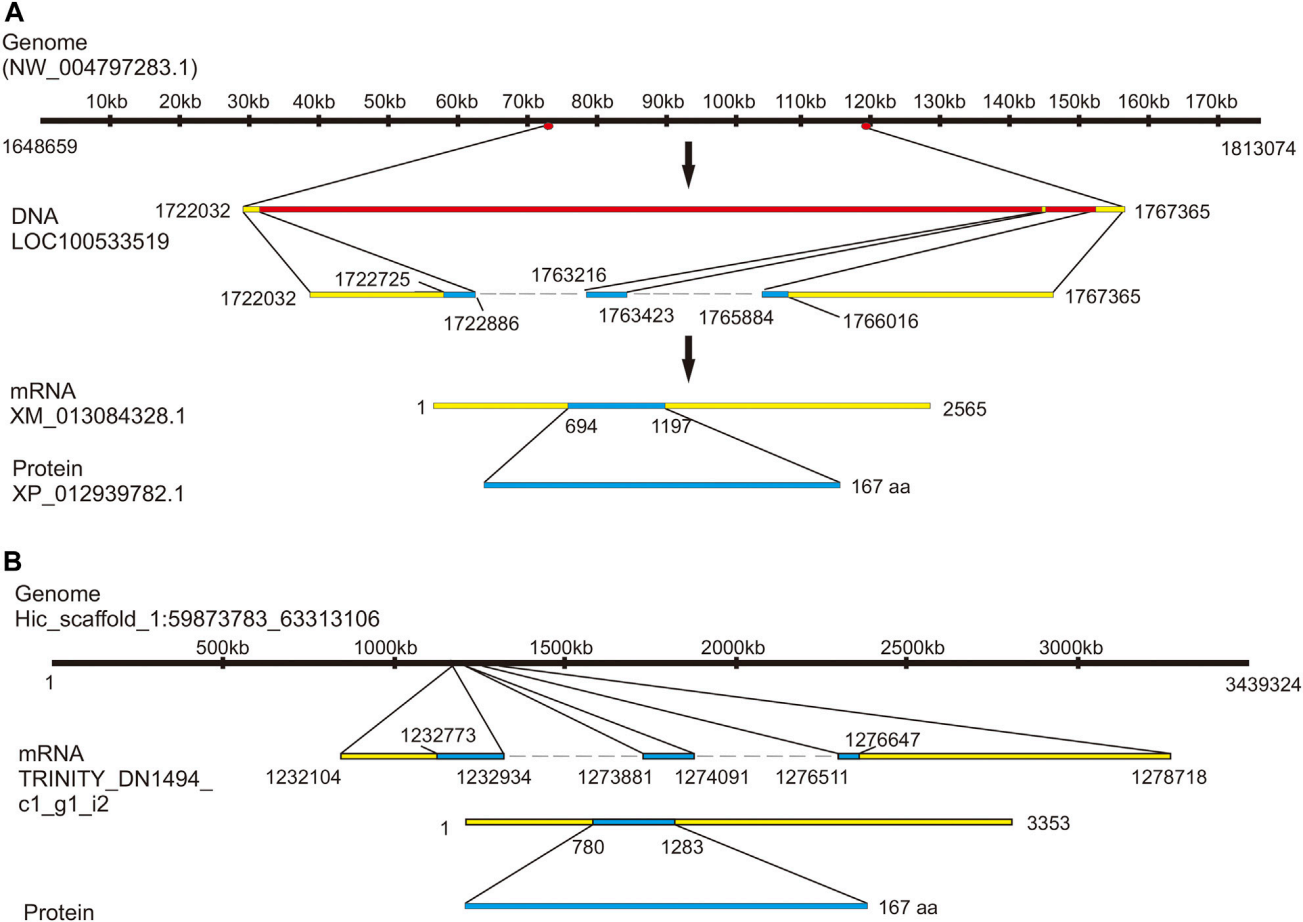
Gene expression mapping of the *Aplysia* vasotcin (apVT) precursor. **(A)** A genome sequence from NCBI (NW_004797283.1) expresses one gene: LOC100533519 (product: Lys-conopressin preprohormone as named in NCBI). The gene corresponds to a mRNA (XM_013084328.1, with two introns: one between 1722886 bp to 1763216 bp and the other between 1763423 bp to 1765884 bp), which produces a protein (XP_012939782.1): Lys-conopressin preprohormone isoform X1. **(B)** Genome Hic_scaffold_1:59873783_63313106 from the *Aplysia* gene nucleotide database (the AplysiaTools) expresses a similar mRNA as in **(A)** (XM_013084328.1) and the protein generated from this mRNA is the same as that in **(A)**, although the 3′and 5′untranslated regions are not identical.

To identify a putative precursor and receptors for *Aplysia* vasotocin (apVT), we began with a bioinformatic analysis. For the precursor, searching “*Aplysia* conopressin” in NCBI returned one entry: two predicted sequences (accession number: XM_013084328.1, which corresponds to a genome sequence: NW_004797283.1; accession number: XM_013084330, which corresponds to the same genome sequence: NW_004797283.1) and a Lys-conopressin precursor deposited in 2008 (mRNA accession number: FJ172359.1), which is likely based on an early large-scale sequencing project (Moroz et al., 2006). The CDS regions of the three sequences are similar: those of XM_013084328.1 and XM_013084330 are identical, which share 15 nucleotides more than that of FJ172359.1. Using the RNA sequence from NCBI (XM_013084328.1), we also found an mRNA sequence (TRINITY_DN1494_c1_g1_i2) in AplysiaTools (see Materials and Methods) (Figure 2B) with the same CDS region with XM_013084328.1 and XM_013084330. Based on this, we plotted gene expression with the sequence XM_013084328.1 (Figure 2A). Note that the mRNA sequence (XM_013084328.1) produces an identical protein as the TRINITY_DN1494_c1_g1_i2 does, but its noncoding regions are somewhat different from the AplysiaTools sequence.

**FIGURE 3:**
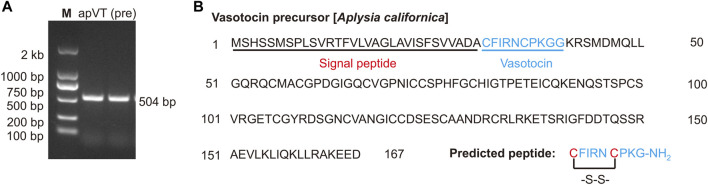
Cloning of the apVT precursor. **(A)** A PCR product for apVT precursor gene with a length of 504 bp. **(B)** The complete protein sequence of the apVT precursor gene illustrates the signal peptide and a predicted peptide in blue: vasotocin. The predicted mature peptide undergoes two post-translational modifications: the last Gly at the C-terminus is typically amidated and the two cysteines (shown in red) form a disulfide bond.

After using bioinformatics to find the potential apVT gene in *Aplysia*, it was important to identify the peptides that are generated by the precursor gene and then find receptors that might be responsive to the peptides. Here, we first designed primers (Supplementary Table S1) using the precursor sequence we found, performed PCR on cDNA of *Aplysia* CNS, and obtained an mRNA of 504 bp in length (Figure 3A, see Supplementary Figure S2A for the complete gels) which is identical to CDS of XM_013084328.1. The sequence we cloned was shown in Supplementary Figure S3. We aligned the precursor of apVT with the homolog precursors in several other species. The result was shown in Supplementary Figure S4 (the similarity of each sequence to apVT is provided in the figure legend). The data indicate that the precursor of apVT has high similarity with the homolog sequence in other species.

Next, we used NeuroPred (Southey et al., 2006) to predict possible peptides that might be generated from the apVT precursor (Figure 3B). The sequence of apVT is: CFIRNCPKGamide, identical to conopressin G. Similar to Vasopressin/Oxytocin in other species, apVT is made up of nine amino acids; the first and sixth of which are cysteines, which form a disulfide bond; the C terminus of apVT is amidated. We also compared apVT with vasopressin/oxytocin in other species (Figure 1A, see Supplementary Table S2 for information on these sequences) and made a frequency plot (Figure 1B) with Weblogo. Given that Vasopressin/Oxytocin has a consistent number of amino acids and posttranslational modifications in different species, we hypothesized that the amino acid sequence and posttranslational modifications of apVT might have some importance, e.g., in receptor activation (see Figures 7–10).

### Identifying putative receptors for *Aplysia* vasotocin

**FIGURE 4:**
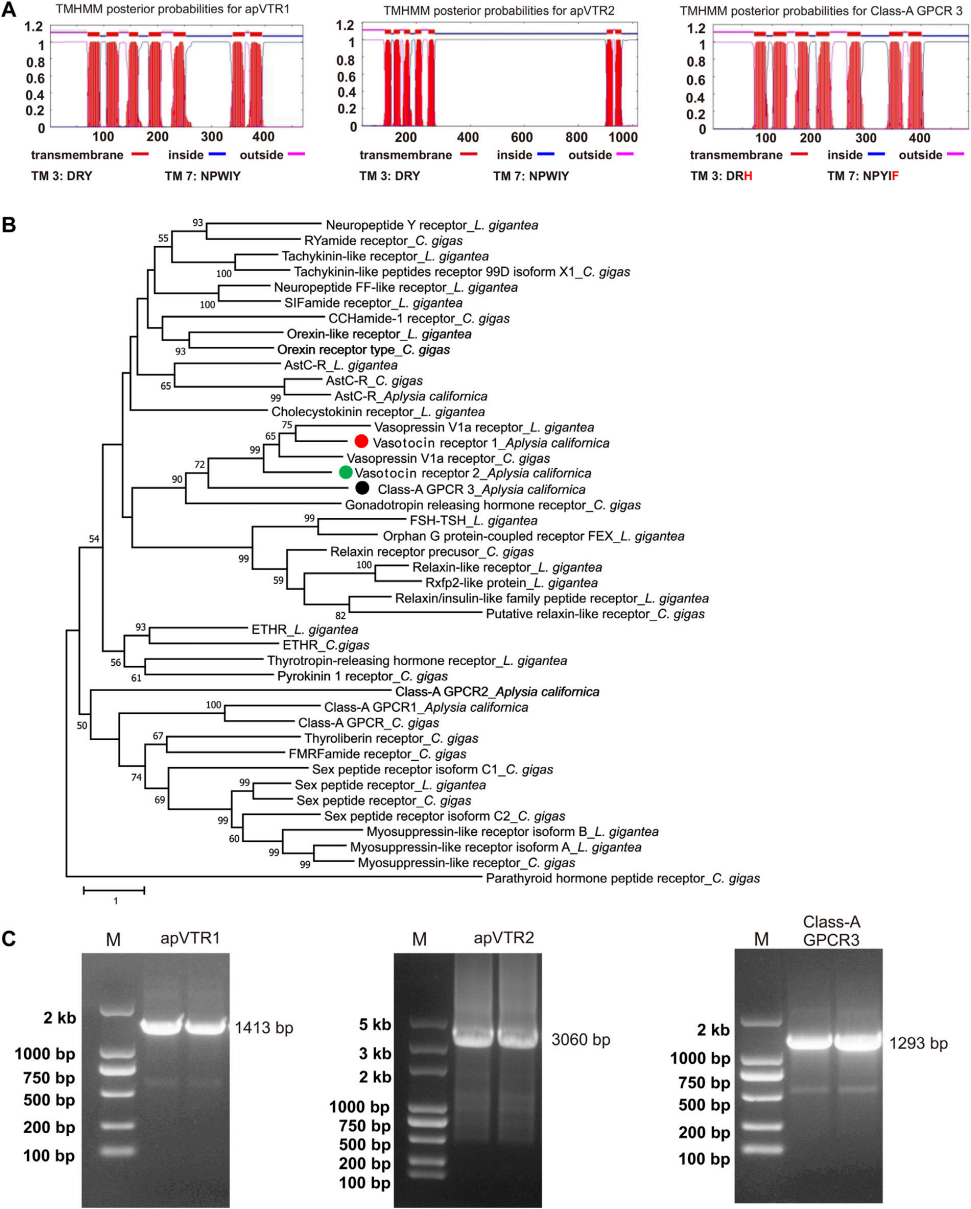
Cloning and bioinformatics of *Aplysia* putative vasotocin receptors (apVTRs). **(A)** Prediction of 7TM of putative receptors (left to right): apVTR1, apVTR2, and Class-A GPCR 3 using TMHMM. Conserved motifs in transmembrane domain 3 (TM3: D/ERY) and TM7 (NPXXY) are shown below. Residues different from conserved motifs are shown in red. **(B)**. A phylogenetic tree of three *Aplysia* putative receptors (apVTR1, apVTR2, Class-A GPCR 3) with a number of molluscan sequences from Jiang et al., 2022 (see the Results and Supplementary Table S3) using MEGA X (see the bioinformatic section of the Methods for more details). A Class-B G protein-coupled receptor: Parathyroid hormone peptide receptor_*C.gigas*, was used as an outgroup. The tree suggests that the three sequences could potentially be vasotocin receptors as they are clustered together with other putative vasotocin/vasopressin receptors. The tree is drawn to scale, with branch lengths measured in the number of substitutions per site. Numbers at the nodes are bootstrap values as a percentage. Only bootstrap values greater than 50 are shown. **(C)** The PCR products of three putative vasotocin receptors (left to right): vasotocin receptor 1 (apVTR1) with a length of 1,413 bp, vasotocin receptor 2 (apVTR2) with a length of 3,060 bp, Class-A GPCR 3 with a length of 1,293 bp.

To identify putative receptors, we searched “Aplysia conopressin receptor” or “Aplysia vasotocin receptor” in NCBI, but this search did not return any sequences. Because of the various naming nomenclatures for vasopressin/oxytocin-like peptides in different species, we then tried to search “Aplysia vasopressin receptor” in NCBI, which did return one sequence (XM_005111551). Then, we searched “Aplysia isotocin receptor” in NCBI, which also returned one sequence (XM_013088972.2). In addition, we used the *Lymnaea stagnalis* conopressin receptor (LSU27464) (van Kesteren et al., 1995b) to blast in the NCBI, which returned yet another possible sequence (XM_005096258). In total, we obtained three sequences. We used these three sequences to blast in the AplysiaTools and found that the third sequence, XM_005096258, appeared to be incomplete compared to a similar sequence (TRINITY_DN90163_c0_g1_i3) in the AplysiaTools. Next, we used NCBI Conserved Domain Search and TMHMM server 2.0 to predict whether these three sequences (XM_005111551, XM_013088972.2, TRINITY_DN90163_c0_g1_i3) are GPCRs. The three sequences are predicted to have 7 transmembrane domains (Figure 4A), which presumably are complete GPCR sequences. In addition to the third sequence, the other two sequences are also present in AplysiaTools databases and have identical CDS regions.

**FIGURE 5:**
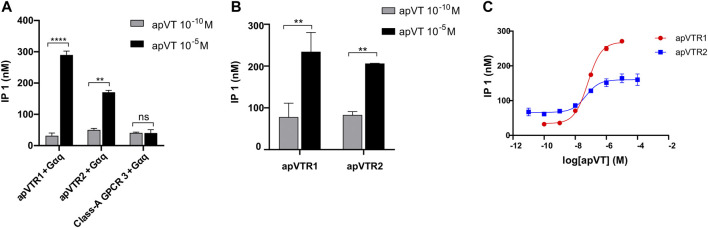
Activation of putative receptors by apVT determined using IP1 accumulation assay. **(A)** Screening of potential activation of apVT on three putative receptors using two apVT concentrations with co-transfection with the promiscuous Gαq: 10^−10^ M and 10^−5^ M. At 10^−10^ M, a peptide activated a receptor minimally, if at all, so it is used as a control. The data indicate that apVTR1 and apVTR2 are receptors for apVT, whereas Class-A GPCR 3 is not. **(B)** Activation of apVTR1 and apVTR2 by apVT using two concentrations without co-transfection with the promiscuous Gαq: 10^−10^ M and 10^−5^ M. Paired *t*-test, ns, not significant; **, *p* < 0.01; ****, *p* < 0.0001; error bar: SEM. **(C)** Representative examples of dose-response curves of the activation of apVTR1 and apVTR2 by the *Aplysia* vasotocin.

To determine whether the three putative GPCRs might be related to apVT receptors, we blasted each sequence in NCBI in four species where more protein sequences have been studied, i.e., *Caenorhabditis elegans*, *D. melanogaster*, *Danio rerio* and *Mus musculus* (Supplementary Table S3). For the protein with accession number: XP_012944426.1 (mRNA: XM_013088972.2), named isotocin receptor in NCBI, a number of sequences named vasopressin receptors or oxytocin receptors with low E-values (<2E-27) came up at these searches in several invertebrate and vertebrate species, suggesting that this protein might be related to apVT receptors. We therefore tentatively named it apVT receptor 1 (apVTR1). For the sequence (TRINITY_DN90163_c0_g1_i3) blasted from AplysiaTools, these searches also returned useful known proteins with low E-values (<7E-13) but low query coverage (<31%), this low query coverage may be due to the long third intracellular loop (ICL3) in the *Aplysia* sequence (Supplementary Table S3). Therefore, we named it as apVT receptor 2 (apVTR2). For the protein XP_005111608.1 (mRNA: XM_005111551.3), the searches returned useful known proteins (such as vasopressin V1a receptor [*M. musculus*]) with low E-values (<5E-13) and high query coverage (>65%). However, through later experiments (see Figure 5A), we determined that the sequence is not a receptor of apVT. Therefore, we used Pfam (http://pfam.xfam.org/search# tabview=tab1) to blast the protein and found that it is classified as Class A GPCRs (rhodopsin family). Thus, we tentatively named this protein (XP_005111608.1) Class-A GPCR 3 (Supplementary Table S3). To provide a better view of the results from Supplementary Table S3, we have included a simplified table as Supplementary Table S4. This Table shows that the apVTR1 and apVTR2 are more similar to vasopressin V1b receptor, whereas Class-A GPCR3 is more similar to vasopressin V1a receptor.

To obtain a phylogenetic relationship between the three proteins, we decided to construct a phylogenetic tree with a number of Class A GPCRs in molluscs *Lottia giagantea* and *Crassostrea gigas* from their Supplementary Table S5 of (Jiang et al., 2022). Then, we added the three *Aplysia* sequences (Supplementary Table S3) and re-ran the phylogenetic tree (Figure 4B). The tree showed that apVTR1 and apVTR2 are clustered together with *C. gigas* vasopressin receptor and *L. giagantea* vasopressin receptor, supporting the hypothesis that apVTR1 and apVTR2 might be apVT receptors. For comparison, although Class-A GPCR 3 is close to these sequences, it is not in the same cluster.

To determine if these sequences are true receptors for apVT, we chose to pursue the study by cloning the two putative apVT receptors and Class-A GPCR 3. We designed primers (Supplementary Table S1) using the three GPCR sequences, and successfully cloned mRNAs for apVTR1 (GenBank accession number: OQ586100), apVTR2 (GenBank accession number: OQ586101), and Class-A_GPCR3 (GenBank accession number: OQ586102) (Figure 4C, see Supplementary Figures S2B–D for the complete gels). The three sequences were shown in Supplementary Figure S5. apVTR1 and apVTR2 have conserved motifs in TM3 with DRY, and TM7 with NPXXY, whereas these motifs in Class-A GPCR 3 are less conserved (TM3: DRH, TM7: NPYIF). We then compared the three putative receptors using BioEdit (Supplementary Figure S6). The similarity between Class-A GPCR 3 and apVTR1 is 28.8%, the similarity between Class-A GPCR 3 and apVTR2 is 23.04%, and the similarity between apVTR1 and apVTR2 is 35.06%. The data indicate that the three sequences have high similarity. To search for other sequences that might be related to the apVT receptors, we used the cloned apVTR sequences to blast both the transcriptome and the genome of the AplysiaTools databases, but we did not find any additional related sequences.

### Activation of the putative receptors by apVT

To determine if these three putative GPCRs are the receptors of apVT, we cloned apVTR1, apVTR2, and Class-A_GPCR3 into pcDNA3.1 plasmids, and expressed them in CHO cells. We then used the IP1 accumulation assay that detects IP1 generated in the Gαq pathway (see Methods) to determine whether the predicted *Aplysia* peptides could activate the receptors. In preliminary experiments, we performed experiments by co-transfecting plasmids with a promiscuous Gαq protein (also known as Gα16) to test if all GPCRs can be activated by apVT. We initially screened IP1 responses of the apVT, at two concentrations (10^–10^ M and 10^–5^ M) on the three receptors: apVTR1, apVTR2, and Class-A_GPCR3 (Figure 5A). At 10^–10^ M, a peptide was typically not or minimally (if any) activating a receptor, so it is used as a control. apVTR1 and apVTR2 responded to apVT (Figure 5A), whereas Class-A_GPCR3 had no response to apVT. We also tested the effects of vasopressin/oxytocin-like peptides from other species (Cone snail (ConS), *Eisenia foetida* (Annetocin), *M. musculus* (Oxytocin and vasopressin) on the Class-A GPCR3 (Supplementary Figure S8), and none of the four peptides had any effects on Class-A GPCR3.

Next, for apVTR1 and apVTR2, we only transfected plasmids for a putative receptor in CHO cells without the promiscuous Gαq protein and obtained similar results (Figure 5B) compared to those when co-transfected with Gαq protein. Thus, for the rest of IP1 accumulation assay, we performed the experiments without co-transfection with Gαq protein. Taken together, we conclude that apVTR1 and apVTR2 are apVT receptors, whereas Class-A_GPCR3 is not.

Furthermore, for the apVTR1 and apVTR2 that had a significant response to apVT in the initial screening (Figure 5B), we used multiple concentrations of the apVT, ranging from 10^–12^ M to 10^–4^ M to determine the dose-response curves of peptide activation on the receptors (Figure 5C). The EC_50_ for the two receptors are similar: apVTR1 (EC_50_ = 70 nM), and apVTR2 (EC_50_ = 77 nM).

**FIGURE 6:**
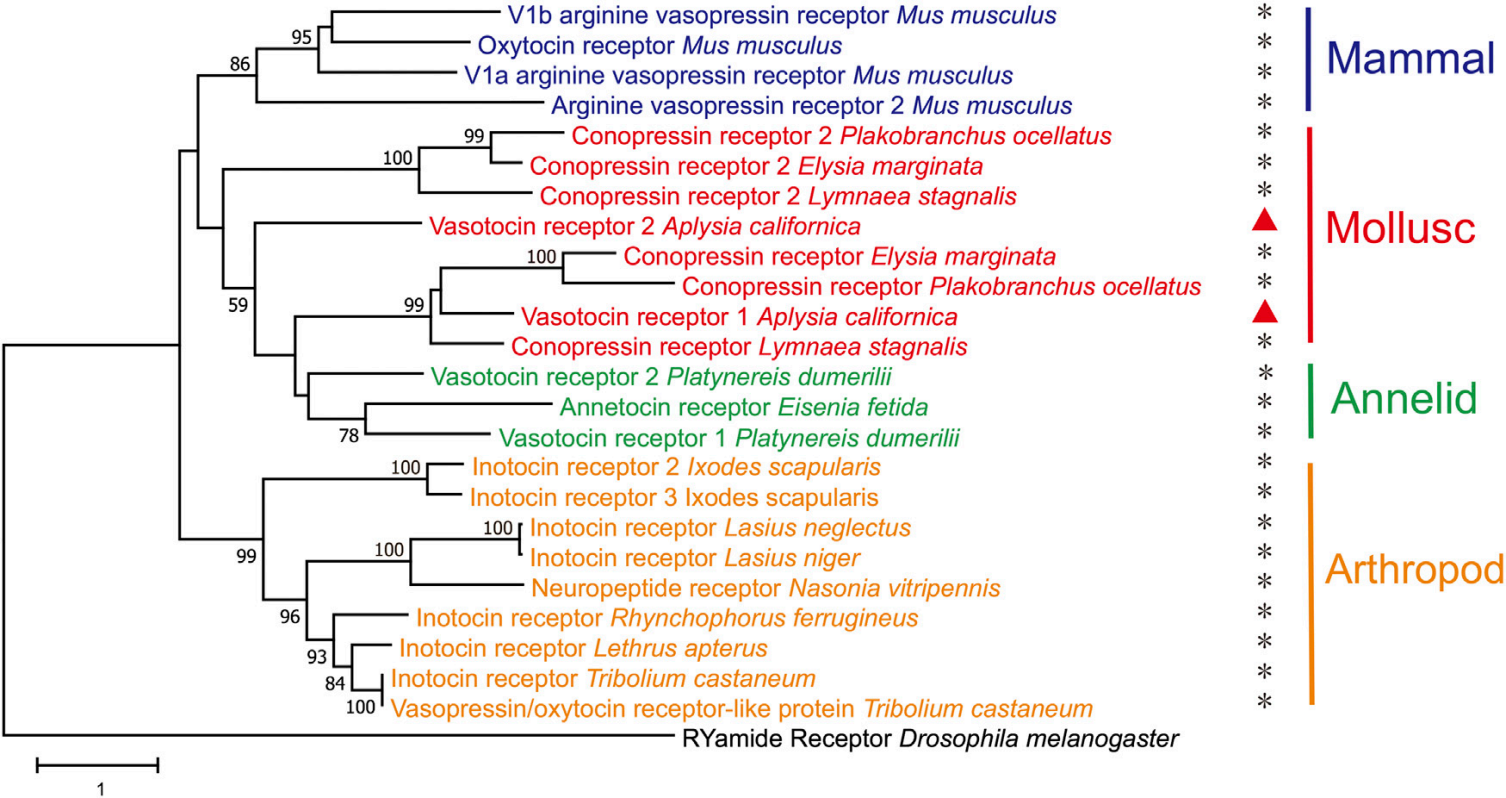
A phylogenetic tree of two *Aplysia* vasotocin receptors with verified vasopressin/oxytocin-like peptide receptors in both protostomes and deuterostomes. The tree was generated using MEGA X with 1,000 replicates (See the bioinformatic section in Methods for more details and Supplementary Table S4 for information on the sequences). “*” indicates that the receptor has been studied/verified. “RYamide receptor_*Drosophila melanogaster*” was used as an outgroup. The tree is drawn to scale, with branch lengths measured in the number of substitutions per site. Numbers at the nodes are bootstrap values as a percentage. Only bootstrap values greater than 50 are shown.

Finally, we generated a phylogenetic tree of the two newly-identified apVT receptors with vasopressin/oxytocin-Rs from selected species in arthropods, molluscs and mammals (Figure 6, see Supplementary Table S5 for information on these sequences. For multiple alignments of the two apVT receptors with vasopressin/oxytocin-Rs from selected species, see Supplementary Figure S7.). The tree suggested that apVTR1 and apVTR2 were closely related to conopression receptors in molluscs, e.g., *Lymnaea*. Notably, the tree also suggested that the vasopressin-like peptide receptors from annelids and molluscs are more closely related to mammalian receptors than the arthropod receptors.

### The roles of post-translational modifications of apVT on receptor activity

**FIGURE 7:**
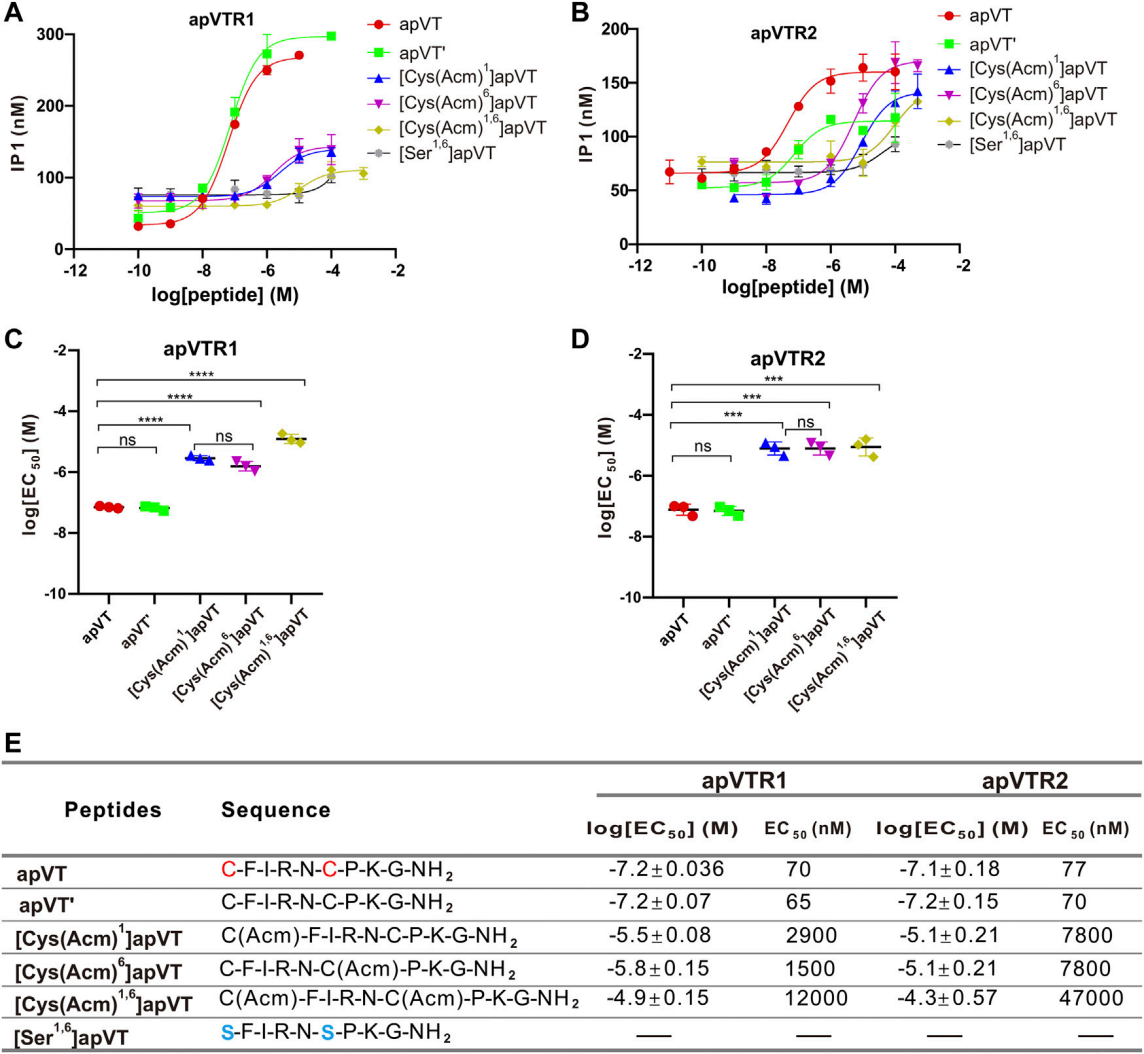
Activation of apVTRs by apVT analogs without the disulfide bond. **(A)** Activation of apVTR1 by apVT analogs without the disulfide bond: apVT’, [Cys (Acm)^1^]apVT, [Cys (Acm)^6^]apVT, [Cys (Acm)^1,6^]apVT and [Ser^1,6^]apVT; apVT was used as a control. **(B)** Activation of apVTR2 by apVT analogs without the disulfide bond: apVT’, [Cys (Acm)^1^]apVT, [Cys (Acm)^6^]apVT, [Cys (Acm)^1,6^]apVT and [Ser^1,6^]apVT; apVT was used as control. **(C)** Comparison of log[EC_50_] for apVTR1 activation by apVT and the five apVT analogs (*n* = 3 for each) shown in **(A)**. One-way ANOVA, F (4, 10) = 249.4, *p* < 0.0001. **(D)** Comparison of log[EC_50_] for apVTR2 activation by apVT and the five apVT analogs (*n* = 3 for each) shown in **(B)**. One-way ANOVA, F (4, 10) = 80.41, *p* < 0.0001. Bonferroni *post hoc* test: ns, not significant; ***, *p* < 0.001; ****, *p* < 0.0001. **(E)** Sequences of apVT and all apVT analogs tested and summary of the average log[EC_50_] and EC_50_ on apVTR1 and apVTR2.

To investigate the effects of the disulfide bond on the activity of apVT, we first synthesized the apVT analog without the disulfide bond: apVT'. However, the results showed that the effects of apVT' on apVTR1 (EC_50_ = 65 nM) and apVTR2 (EC_50_ = 70 nM) were not different from those of apVT (Figure 7), which might imply that the disulfide bond is not important for the activity of apVT. However, previous work has shown that vasopressin/oxytocin without the disulfide bond could spontaneously form a disulfide bond under physiological conditions (Roy et al., 2007). Therefore, we synthesized neuropeptide analogs that protect cysteines with acetamidomethyl (Acm) to prevent the spontaneous formation of the disulfide bond: [Cys(Acm)^1^]apVT (Acm protects only the first cysteine), [Cys(Acm)^6^]apVT (Acm protects the second cysteine) and [Cys(Acm)^1,6^]apVT (Acm protects both cysteines). In addition to the protection of cysteine residues, we also used serines to substitute the cysteines [(Ser^1,6^)apVT] (Labarrere et al., 2003). The results showed that the effect of apVT analogs without the disulfide bond on the two receptors was significantly reduced (Figure 7), indicating that the disulfide bond is important for the function of apVT.

**FIGURE 8:**
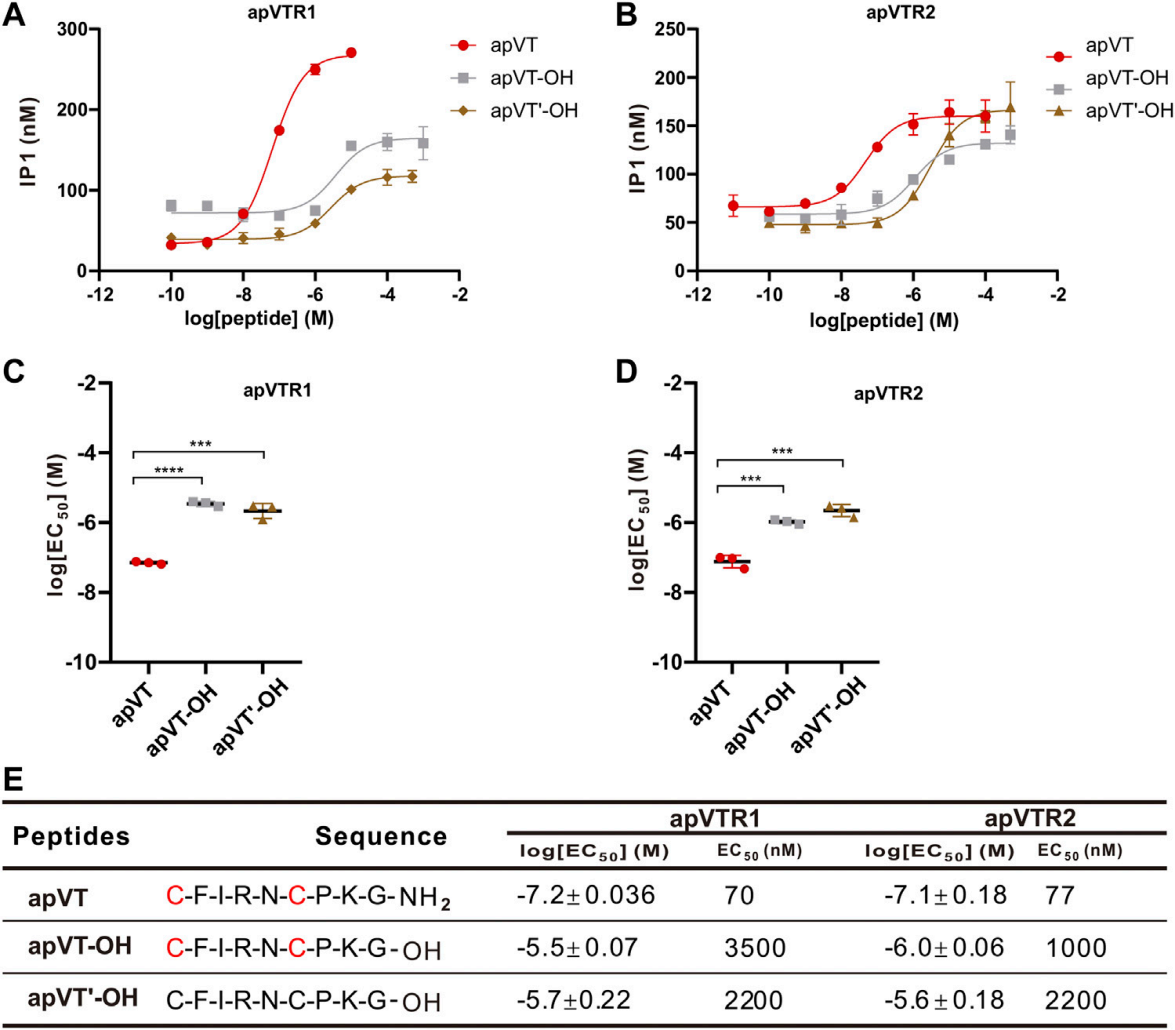
Activation of apVTRs by apVT analogs without the C-terminal amidation. **(A)** Representative examples of activation of apVTR1 by apVT analogs without the C-terminal amidation: apVT-OH and apVT’-OH; apVT was used as a control. **(B)** Activation of apVTR2 by apVT without the C-terminal amidation: apVT-OH and apVT'-OH; apVT was used as control. **(C)** Comparison of log[EC_50_] for apVTR1 by apVT and the two apVT analogs (n = 3 for each) shown in **(A)**. One-way ANOVA, F (2, 6) = 143.4, *p* < 0.0001. **(D)** Comparison of log[EC_50_] for apVTR2 by apVT and the two apVT analogs (*n* = 3 for each) shown in **(B)**. One-way ANOVA, F (2, 6) = 80.89, *p* < 0.0001. Bonferroni *post hoc* test: ***, *p* < 0.001; ****, *p* < 0.0001. **(E)** Sequences of apVT and all apVT analogs tested and summary of the average log[EC_50_] and EC_50_ on apVTR1 and apVTR2.

To investigate the effect of the C-terminal amidation on the activity of apVT, neuropeptide analogs without the C-terminal amidation were synthesized: apVT-OH (C terminus without amidation, but with the disulfide bond), apVT’-OH (C terminus un-amidated, and without the disulfide bond) (Figure 8). EC_50_ values of apVT-OH on apVTR1 and apVTR2 were 3,500 nM and 1,000 nM respectively, and EC_50_ values of apVT’-OH on apVTR1 and apVTR2 were 2,200 nM and 2,200 nM respectively (Figure 8E). These results showed that apVT-OH and apVT’-OH had significantly weaker effects on apVTR1 and apVTR2 than apVT, indicating the C-terminal amidation is important for the function of apVT.

### The roles of single residues of apVT on receptor activity

**FIGURE 9:**
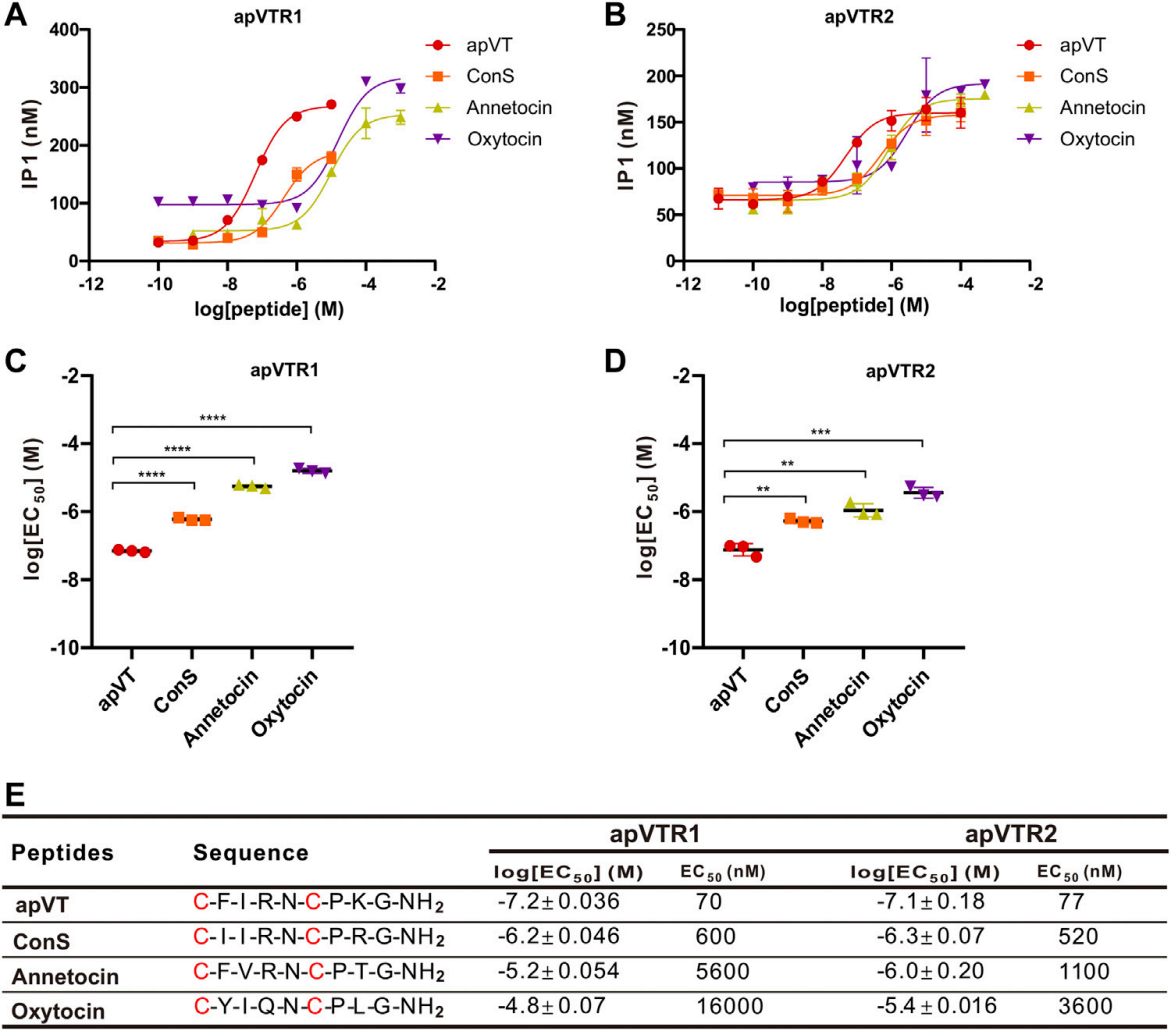
Activation of apVTRs by vasopressin/oxytocin-like peptides from other species. **(A)** Activation of apVTR1 by vasopressin/oxytocin-like peptides: ConS, annetocin, and oxytocin; apVT was used as a control. **(B)** Activation of apVTR2 by vasopressin/oxytocin-like peptide: ConS, annetocin, oxytocin; apVT was used as a control. **(C)** Comparison of log[EC_50_] for apVTR1 by the four peptides (*n* = 3 for each) shown in **(A)**. One-way ANOVA, F (3, 8) = 1,158, *p* < 0.0001. **(D)** Comparison of log[EC_50_] for apVTR2 by the four peptides (*n* = 3 for each) shown in **(B)**. One-way ANOVA, F (3, 8) = 58.27, *p* < 0.0001. Bonferroni *post hoc* test: **, *p* < 0.01; ***, *p* < 0.001; ****, *p* < 0.0001. **(E)** Sequences of apVT and all apVT analogs tested and summary of the average log[EC_50_] and EC_50_ on apVTR1 and apVTR2.

In addition to the C-terminal amidation and the disulfide bond, vasopressin/oxytocin-like peptides had a relatively consistent number of residues in different species, with some residues completely conserved, whereas other residues are less conserved (Figure 1). To determine if the vasopressin-like peptides in other species with some sequence difference to apVT have any activity on apVTR1 and apVTR2, we first synthesized vasopressin/oxytocin-like peptides from other species: ConS, annetocin, oxytocin, vasopressin. We found that ConS, annetocin, and oxytocin have various effects on apVTR1 and apVTR2 (Figure 9), whereas vasopressin had no effects on these two receptors (Supplementary Figure S9B). These results suggest that different residues in the ligands might play a role in receptor activation. However, because of the variations of amino acids in the number and position in the above-mentioned vasopressin/oxytocin-like peptides, a conclusion about the specific effects of individual residues of apVT on receptor activity cannot be drawn.

**FIGURE 10:**
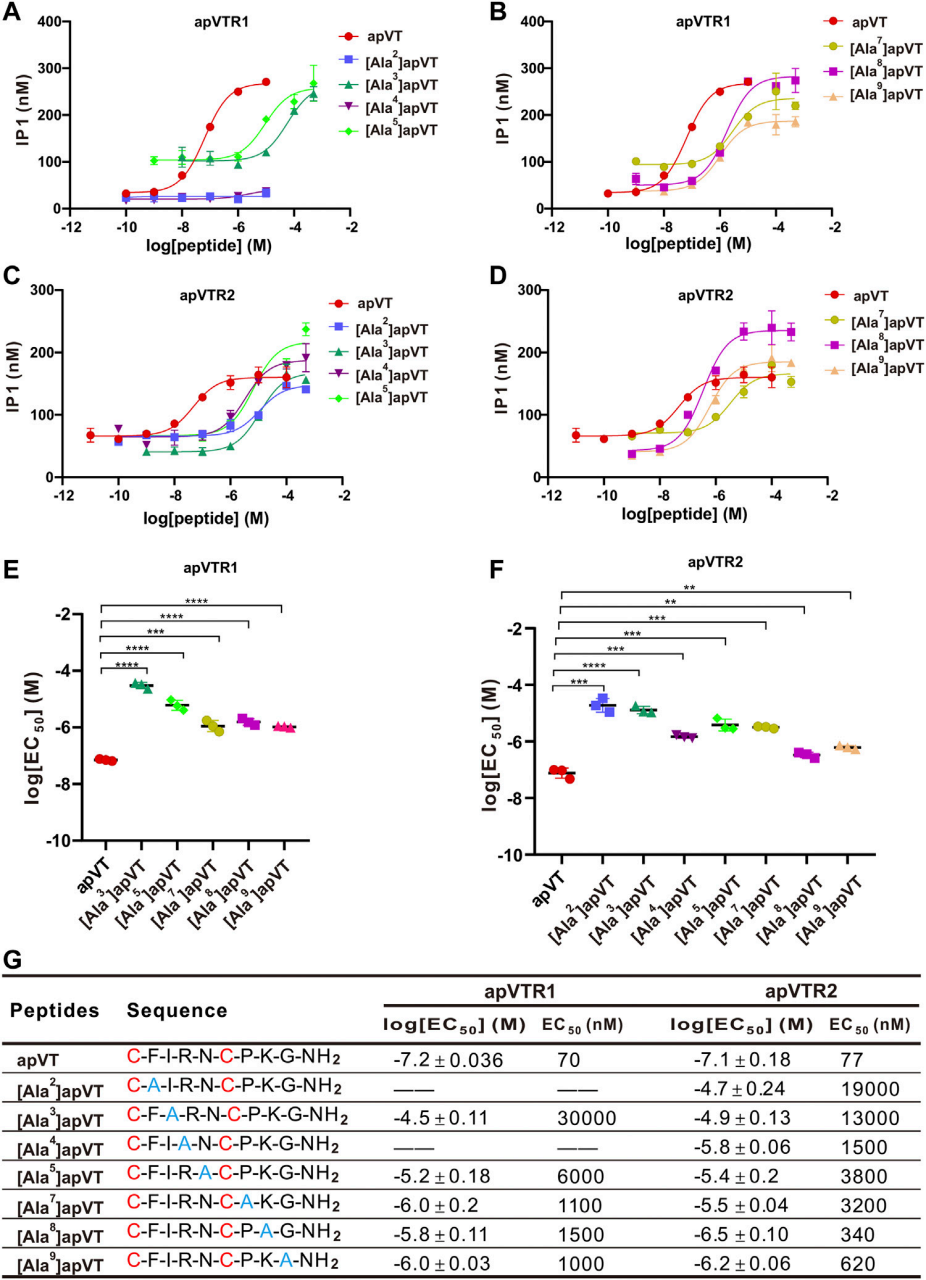
Activation of apVTRs by apVT analogs with alanine substitution. **(A, B)** Representative examples of dose-response curves of the activation of apVTR1 by the apVT analogs with alanine substitution: [Ala^2^]apVT, [Ala^3^]apVT, [Ala^4^]apVT, [Ala^5^]apVT, [Ala^7^]apVT, [Ala^8^]apVT, [Ala^9^]apVT; apVT was used as a control. **(C, D)** Dose-response curves of the activation of apVTR2 by apVT analogs with alanine substitution: [Ala^2^]apVT, [Ala^3^]apVT, [Ala^4^]apVT, [Ala^5^]apVT, [Ala^7^]apVT, [Ala^8^]apVT, [Ala^9^]apVT; apVT was used as a control. **(E)** Comparison of log[EC_50_] for apVTR1 by apVT and the five apVT analogs (*n* = 3 for each) shown in **(A, B)**. One-way ANOVA, F (5, 12) = 142, *p* < 0.0001. **(F)** Comparison of log[EC_50_] for apVTR2 by apVT and the seven apVT analogs (*n* = 3 for each) shown in **(C, D)**. One-way ANOVA, F (7, 16) = 93.92, *p* < 0.0001. Bonferroni *post hoc* test: **, *p* < 0.01; ***, *p* < 0.001; ****, *p* < 0.0001. **(G)** Sequences of apVT and all apVT analogs tested and summary of the average log[EC_50_] and EC_50_ on apVTR1 and apVTR2.

To determine the roles of specific residues in apVT in the activity on the receptors, we used alanine to replace the other residues except for the cysteines in apVT. Seven types of analogs were synthesized. We determined the dose-response curves of peptide analog activation on the receptors (Figure 10). These results showed that the changes of residues in different positions had various effects on receptor activity. Overall, the residues within the disulfide bond tended to have larger effects than residues outside the disulfide bond.

### Distribution of apVT precursor and apVTR1 in *Aplysia* tissues

We illustrated the expression of the apVT precursor and its receptors using a NCBI database from a broad spectrum of RNA-seq data (GSE79231) obtained from adult and developmental stages (Moroz et al., 2006; Gyori et al., 2021). For apVT precursor (XM_013084328.1), it is most highly expressed in the CNS. It is also expressed in digestive organs, esophagus, and hepatopancreas (Supplementary Figure S10A). For the receptors, the database only included information for apVTR1 (XM_013088972.2). apVTR1 appears to be highly expressed in mantle, and digestive organs. It is also present in the CNS (Supplementary Figure S10B). Currently, there were no data available for apVTR2 (the accession number in NCBI: XM_005096258) in the database (GSE79231).

## Discussion

Using bioinformatics, molecular biology, and cell-based assay, we have obtained the precursor for *Aplysia* vasotocin from the *Aplysia* CNS and provided the first evidence for the presence of two receptors. We also explored the roles of individual residues and PTMs in the activation of the two receptors.

### Identification of the *Aplysia* vasotocin signaling system

Previous work has suggested that *Aplysia* vasopressin-like peptide is present in the *Aplysia* CNS using an antibody against Lys-vasopressin and an antibody against arginine vasotocin (Moore et al., 1981; Martinez-Padron et al., 1992). The precursor we identified provides further evidence supporting this idea. Our initial database searches returned three similar sequences (Figure 2), but the predicted CDS sequence of one of them (NCBI: FJ172359.1) is shorter than the other two (NCBI: XM_013084328.1, and AplysiaTools: TRINITY_DN1494_c1_g1_i2) partly because at the N terminus, there are two start codons (ATG). We also used https://www.ncbi.nlm.nih.gov/orffinder/ to predict the CDS region of FJ172359.1 and found that the CDS region is actually longer, and is consistent with that of XM_013084328.1. Thus, we designed primers for the longer CDS sequence and successfully cloned this precursor from the *Aplysia* CNS tissues (mostly pedal ganglia). Taken together, our study supports that the CDS sequence is the longer version rather than the shorter one. Importantly, the peptide predicted from the precursor matched the Lys-cononpressin G as predicted before (Martinez-Padron et al., 1992). We used the *A. californica* RNA sequencing database (GSE79231) in NCBI to show that the precursor is present in the CNS, consistent with the previous work (Moore et al., 1981; Martinez-Padron et al., 1992). In addition, the data also showed that the precursor is most highly expressed in the CNS compared to peripheral tissues (Supplementary Figure S10A).

We found that there are at least two receptors for apVT. Our initial bioinformatic analysis suggests that there might be up to three receptors, which we have cloned. The IP1 accumulation assay experiments demonstrated that two of three receptors, apVTR1, and apVTR2, are true receptors for apVT, whereas the third one (Class-A GPCR3) is not. Notably, apVTR2 is distinct in that it has a long intracellular loop (636 aa) between the fifth and sixth transmembrane domains, compared to ∼70 aa for apVTR1. To our knowledge, this long intracellular loop appears to be also present in another mollusc *Theba pisana* (Stewart et al., 2016) among a number of vasopressin/oxytocin receptors in the species we examined. Based on the phylogenetic relationship (Figure 4B), the third putative receptor, Class-A GPCR3, appears to be somewhat related to apVTR1 and apVTR2, but it is not responsive to apVT. We also tested other *Aplysia* peptides with the disulfide bond, e.g., Urotensin II (Romanova et al., 2012), AstC (Jiang et al., 2022) on the Class-A GPCR3, but it is not responsive to any of these either (Supplementary Figure S9A). Thus, this receptor might be sensitive to some unknown *Aplysia* peptides, possibly with a disulfide bond. Regardless, our data indicate that there are at least two receptors for apVT, similar to *Lymnaea* (Van Kesteren et al., 1995a; van Kesteren et al., 1996). Interestingly, the vasopressin-like peptide receptors from the superphylum lophotrochozoa, which includes annelids and molluscs, are more closely related to mammalian receptors, than the arthropod receptors (Figure 6). Similar findings have been reported for other *Aplysia* proteins (Moroz et al., 2006; Jing et al., 2015). Notably, the *A. californica* RNA sequencing database (GSE79231) in NCBI showed that apVTR1 is present in the CNS, in addition to some peripheral tissues (Supplementary Figure S10B), although data on apVTR2 was unavailable.

In mammals, as a closely related neuropeptide, oxytocin (OT) is structurally similar to vasopressin (AVP), with differences in only two AA residues at positions 3 and 8 (Figure 1B). At position 8, oxytocin is the neutral amino acid Leu, whereas vasopressin is the basic amino acid Arg (Turner et al., 1951; Tuppy, 1953). In terms of the eighth amino acid, it is the basic amino acid Lys in apVT, which is similar to the eighth amino acid (Arg) of vasopressin in nature but differs from that (Leu) of oxytocin, which is neutral, implying that apVT may be more similar to vasopressin than to oxytocin. On the other hand, previous functional studies implicated invertebrate oxytocin/vasopressin-like neuropeptides in the regulation of reproduction (Oumi et al., 1996; Koene, 2010; Garrison et al., 2012), suggesting that invertebrate oxytocin/vasopressin-like peptide might be evolutionarily more similar to vertebrate oxytocin because oxytocin plays significant roles in reproduction (Carter et al., 2020). Moreover, our IP1 accumulation experiments showed that the mammalian oxytocin could have a weak activation effect on both of the two *Aplysia* receptors, whereas the mammalian vasopressin had no effects on the two receptors (Figure 9, and Supplementary Figure S9B). Thus, these results support that the apVT is more similar to mammalian oxytocin rather than to vasopressin, which seems to contradict the structural similarity. Perhaps, this could be partly explained in part by our alanine substitution experiments, where, when the eighth amino acid of apVT was replaced with the neutral alanine, the effects on the receptor activity were relatively weak compared with the alanine substitution of the other residues (Figure 10). Another possible explanation could be that the third residue of apVT is identical to the one in oxytocin, and the alanine substitution of this third residue caused much larger effects.

### Actions of PTMs and single residues of apVT on receptor activity

We have determined the roles of PTMs of apVT on the activity of the receptors (Figure 7). Initially, our experiments with apVT without the disulfide bond actually have similar EC_50_ values on both receptors compared to the one with the disulfide bond. However, this experiment did not necessarily show that the disulfide bond is not important for receptor activity because previous work has shown that oxytocin without the disulfide bond can spontaneously form a disulfide bond (Roy et al., 2007). Indeed, when we used acetamidomethyl (Acm) to protect either one or two cysteines to prevent cysteines from forming the disulfide bond, the EC_50_ values became significantly higher. When the cysteines were substituted by serines, the apVT analog has no obvious effect on the apVTR1 and apVTR2. Consistent with this result, previous work also showed that when the disulfide bond was replaced with other types of chemical rings in oxytocin, some of the peptide analogs can still be active on the oxytocin receptor depending on the bond length and torsion angle (Muttenthaler et al., 2010; Adachi et al., 2017). Taken together, our data support that the disulfide bond is important for receptor activity.

We also tested the roles of C-terminal amidation on receptor activity by removing it. The data showed that amidation appears to be important for the activity of both receptors (Figure 8). To our knowledge, this is the first evidence that C-terminal amidation might be important for receptor activity for vasopressin/oxytocin receptors. We expect that, if vasopressin or oxytocin in mammals were removed from its C-terminal amidation, similar effects could be observed on their receptors, although this remains to be tested formally.

It is interesting to note that despite the highly conserved sequences of vasopressin/oxytocin among different species, the activity of vasopressin/oxytocin from three different species (a mollusc, an annelid, and a mammal) varied significantly from apVT on both *Aplysia* receptors (Figure 9, Supplementary Figure 5B). Nevertheless, the data did suggest that, other than the disulfide bond and C-terminal amidation, individual residues might also play some roles in receptor activity. We formally tested the roles of individual residues on receptor activity by performing alanine substitution experiments. Many previous studies have shown that changes in evolutionarily conserved residues in peptide ligands have a significant impact on, and usually are necessary for, receptor activity (Guo et al., 2022). For oxytocin/vasopressin, except for cysteine residues, the residues at the position of 5, 7, and 9 are more evolutionarily conserved than the other residues. However, after replacing the three residues with alanine, EC_50_ values of these analogs were not necessarily higher compared with analogs with the replacement of the residues less conserved (Figure 10), which is somewhat unexpected. On the other hand, we found that changes in residues within the disulfide bond ring seem to have a greater impact on receptor activity than those residues outside the ring, similar to results obtained in previous work (Adachi et al., 2017; Kinoshita et al., 2021). Similar results using bioassay were also obtained for human urotensin II with a disulfide bond (Labarrere et al., 2003). Overall, our findings are in general consistent with findings on vasopressin/oxytocin in vertebrates.

Notably, the two *Aplysia* receptors appear to have different sensitivities to the alanine substitution of apVT. Specifically, we found that the effects of peptide analogs on the two receptors were significantly different when the second and fourth residues of apVT were substituted with alanine. Peptide analogs with the second and fourth residues substituted could not activate apVTR1 at all, but still had some effects on apVTR2, although the effects were weakened compared with apVT. It would be of interest to investigate why the two receptors had different sensitivity, perhaps by molecular modeling of the ligand and peptide analogs with the two receptors in the future.

From a drug development perspective in mammals, the importance of oxytocin/vasopressin, particularly oxytocin, in their actions as a drug has been discussed previously (Carter et al., 2020). For example, oxytocin can function as a stress-coping molecule, an anti-inflammatory, and an antioxidant reagent, with protective effects, especially in the face of adversity or trauma. Oxytocin influences the autonomic nervous system and the immune system. These properties of oxytocin may help explain the benefits of positive social experiences and have drawn attention to this molecule as a possible therapeutic in a host of disorders (Carter et al., 2020). In addition, the effects of cone snail venom peptides with sequences similar to oxytocin/vasopressin, i.e., conopressins, have been extensively studied (Lewis et al., 2012). These cone snail venom peptides are used for prey capture and/or defense for these animals (Ramiro et al., 2022). Indeed, many cone snail venoms, including conopressins, contain peptides with two or more cysteines, and act on membrane proteins, e.g., voltage- or ligand-gated ion channels (including GPCRs) (Lewis et al., 2012; Koch et al., 2022), or act as hormones (Robinson et al., 2017; Turner et al., 2020). Thus, our present results might help provide some insights into how to design better drugs for medicine (Walter et al., 1971; Vrachnis et al., 2011; Ichinose et al., 2019).

In summary, our study provides a relatively complete description of the *Aplysia* vasopressin signaling system by identifying the precursor and two receptors and exploring the roles of PTMs and individual residues in receptor activity. Future work could investigate how the peptide ligand might interact with the receptors and possibly explain how PTMs and individual residues might contribute to receptor activation through structural modeling (Guo et al., 2022). Additionally, the physiological actions of the *Aplysia* vasotocin signaling system need to be explored more extensively. Preliminary work has shown mRNA distributions of apVT and apVTR1 in *Aplysia* tissues (Supplementary Figure S10). Previous work has also shown that neurons with vasopressin-like immunoreactivity are present in the CNS (Martinez-Padron et al., 1992). It would be interesting to determine the neuronal distributions of the two receptors, and possibly provide clues on what kinds of behavioral networks the *Aplysia* vasopressin signaling system may act on. Given the diverse roles of vasopressin/oxytocin in mammalian neural functions, these studies in *Aplysia* in particular, and molluscs, in general, may also provide a better understanding of how the vasopressin signaling system has evolved.

## Data Availability

The original contributions presented in the study are included in the article/Supplementary Material, further inquiries can be directed to the corresponding authors.
