# Supplementary material for: Characterization of an Aplysia vasotocin signaling system and actions of posttranslational modifications and individual residues of the ligand on receptor activity
Source: Front Pharmacol. 2023 Mar 20;14:1132066. doi: 10.3389/fphar.2023.1132066 (PMC10067623; doi:10.3389/fphar.2023.1132066)
Supplement: Supplementary file 3 [file DataSheet1.PDF]

Contents

apVT……………………………………………………………………………....1

Vasopressin………………………………………………………………….....4

Oxytocin…………………………………………………………………….....7

Conopressin S………………………………………………………….....….10

Annetocin.………………………………………………………………..….13

apVT'........……………………………………………………………....….16

[Cys(Acm)^{1}]apVT..………………………………………………….....….19

[Cys(Acm)^{6}]apVT……………………………......………………….....….22

[Cys(Acm)^{1,6}]apVT……….....……………………………………….....….25

[Ser^{1,6}]apVT……….....……………………………………...…...........28

apVT-OH……………………………....…………………………………....31

apVT'-OH……………………………........…………………………….…..34

[Ala^{2}]apVT...………………………………………………........……………...37

[Ala^{3}]apVT…………………...……………………………………………….…..40

[Ala^{4}]apVT…………………………………………........……………………….43

[Ala^{5}]apVT……………………………………………………………………….46

[Ala^{7}]apVT……………………………………………………………………….49

[Ala^{8}]apVT……………………………………………………………………….52

[Ala^{9}]apVT……………………………………………………………………….55

apVT

安徽省国平药业有限公司

CERTIFICATE OF ANALYSIS

Order ID	GP120349 -1
Name	Vasotocin
Lot No.	GP120349 -1-1124
CAS	CFIRNCPKG -NH2
Dissolution condition	100%H2O
Length	9AA
Modification	形成二硫键
Molecular Weight (MW)	1034.26
Storage	-20℃

Test Items	Specifications	Results
MW by MS	1033.70	Conforms
Purity by HPLC	>95%	96.911%
Peptide Content	N/A	N/A
Moisture content	N/A	N/A
Acetic acid content	N/A	N/A
Appearance	White to off-white lyophilized powder	Conforms
Quantity	4mg	4.0mg

Certified by: LiuHui Date 12/07/2020

Quality Assurance Department

Note: this product is intended for research use only; not for diagnostic or human use.

Guoping Pharmaceutical Co., LTD

地址:合肥市经开区桃花工业园拓展区工投立恒工业广场 A2 西F1,电话:0551-62841987 传真:0551-62841765 www.guopingyaoye.com

apVT

SampleInformation

OrderID:GP120349-1
Name: Vasotocin
Sequence: C*FIRNC*PKG-NH2
Lot.No: GP120349-1-1124
PumpA: 0.1%TriĐluoroaceticin100%water
PumpB: 0.1%TriĐluoroaceticin100%acetonitrile
TotalFlow:1ml/min
Wavelength:220nm
Analyticalcolumntype:SHIMADZUInertsilODS-SP(4.6*250mm*5um)
Dissolutionmethod:100%H2O
Inj.Volme:10uL

Time	Module	Action	Value
0.01	Pumps	B.Conc	10
20.00	Pumps	B.Conc	50
30.00	Pumps	B.Conc	100
38.00	Pumps	B.Conc	100
40.00	Pumps	B.Conc	10
50.00	Controller	Stop	

Chromatogram

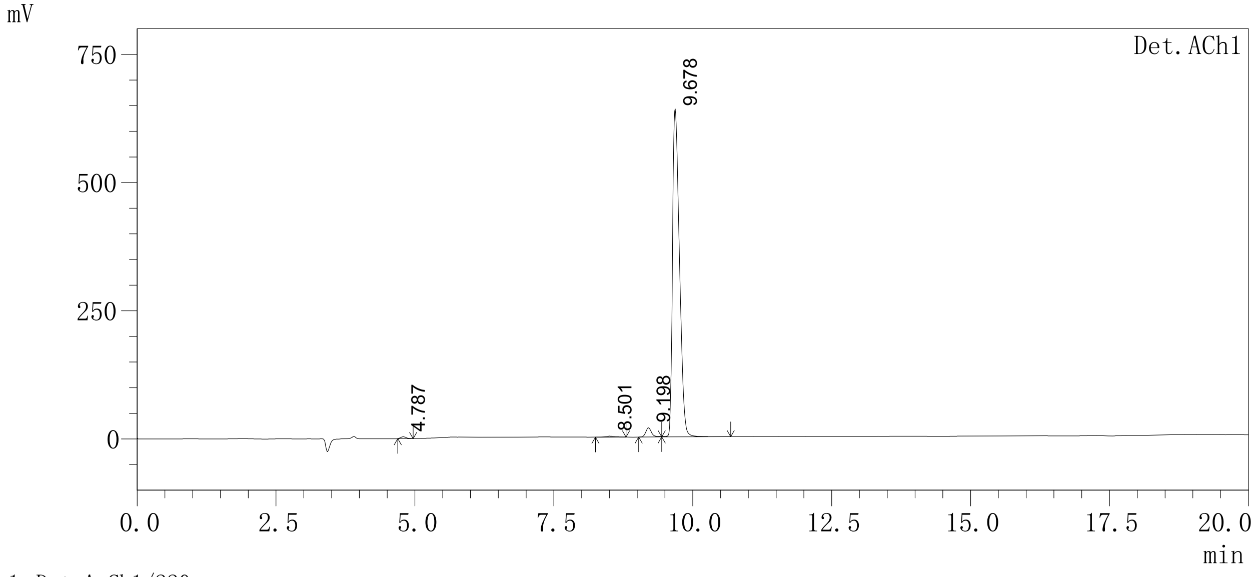

1 Det.A Ch1/220nm

PeakTable

DetectorACh1220nm

Peak#	Ret.Time	Area	Height	Area%	Height%
1	4.787	24059	3643	0.421	0.549
2	8.501	21414	1833	0.375	0.276
3	9.198	131158	17901	2.294	2.699
4	9.678	5540769	639928	96.911	96.476
Total		5717401	663306	100.000	100.000

apVT

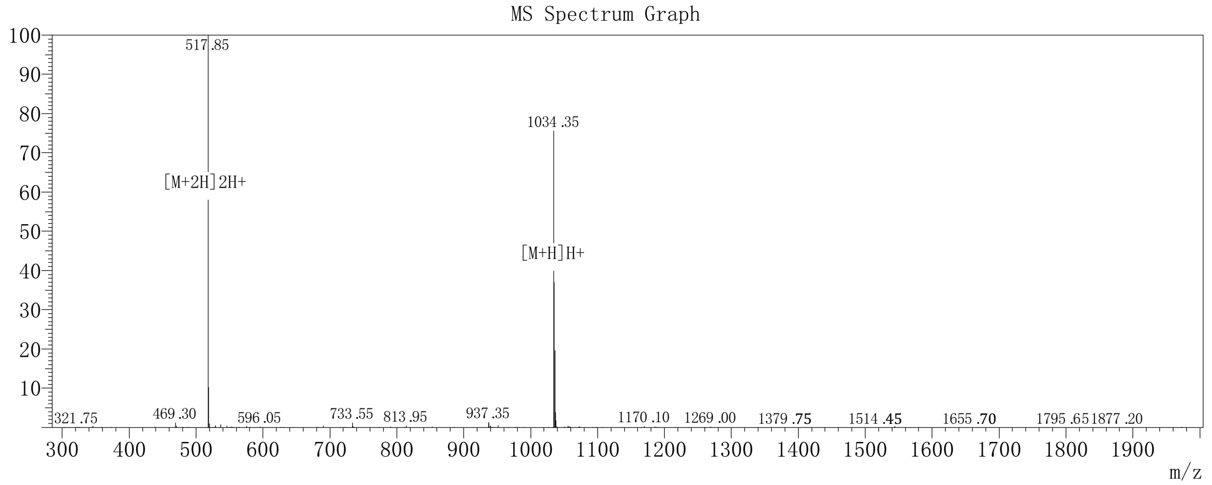

Sample Information

Dissolution method	:5%HAC+8%ACN+87%H2O	Interface	:ESI	Prerod Bias	:+1.5kv
Modified Date	:2020/12/05	Nebulizing Gas Flow	:1.50L/min	Detector	:-0.2kv
Injection Volume	:1ul	CDL Temp	:250C	T.Flow	:0.2ml/min
Heat Block Temp	:200	CDL Volt	:0v	B.conc	:50%H2O/50%MEOH

Order ID	:GP120349-1
Name	:Vasotocin
Sequence	:C*FIRNC*PKG-NH2
Lot.No	:GP120349-1-1124
Theoretical	:1034.26
Observed	:1033.70

Vasopressin

安徽省国平药业有限公司

CERTIFICATE OF ANALYSIS

Order ID	GP120500-3
Name	Vasopressin
Lot No.	GP120500-3-1015
Sequence	C-Y-F-Q-N-C-P-R-G*
Dissolution condition	100%H2O
Length	9AA
Modification	（第一个Cys和第六个Cys之间形成二硫键，最后一个Gly酰胺化）
Molecular Weight (MW)	1084.25
Storage	-20° C

Test Items	Specifications	Results
MW by MS	1084.20	Conforms
Purityby HPLC	>95%	96.227%
Peptide Content	N/A	N/A
Moisture content	N/A	N/A
Acetic acid content	N/A	N/A
Appearance	White to off-white lyophilized powder	Conforms
Quantity	5mg	1.0mg*5

Certified by: LiuHui　　Date 10/28/2021

Quality Assurance Department

Note: this product is intended for research use only; not for diagnostic or human use.

Guoping Pharmaceutical Co., LTD

地址:合肥市经开区桃花工业园拓展区工投立恒工业广场A2 西F1,电话:0551-62841987 传真:0551-62841765 www.guopingyaoye.com

Vasopressin

SampleInformation

OrderID:GP120500-3
Name:Vaso pressin
Sequence:C*-Y-F-Q -N-C*-P-R-G-NH2
Lot.No: GP120500-3-1015
PumpA: 0.1%TriÐluoroaceticin100%water
PumpB: 0.1%TriÐluoroaceticin100%acetonitrile
TotalFlow:1ml/min
Wavelength:220nm
Analyticalcolumntype:SHIMADZUInertsilODS-SP(4.6*250mm*5um)
Dissolutionmethod:100%H2O
Inj.Volme:12uL

TimeModule	Action	Value
0.01Pumps	B.Conc	10
20.00Pumps	B.Conc	50
23.00Pumps	B.Conc	100
38.00Pumps	B.Conc	100
40.00Pumps	B.Conc	10
50.00Controller	Stop	

Chromatogram

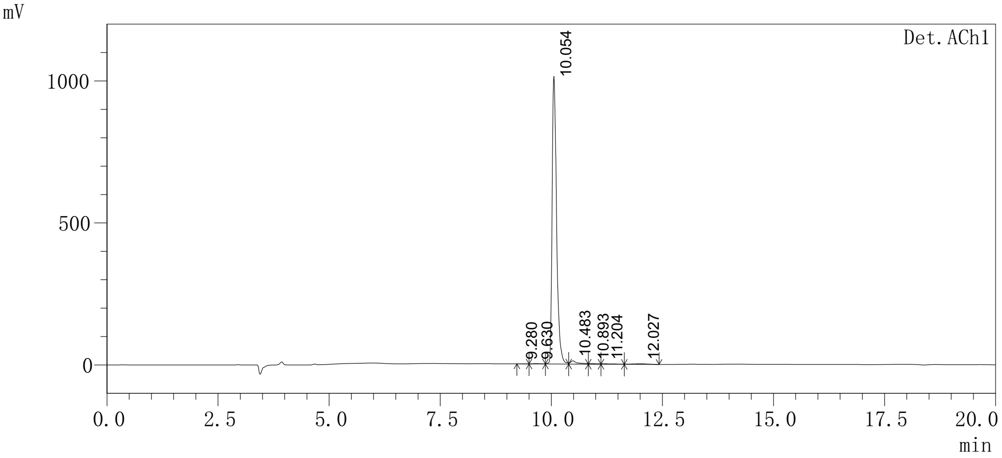

1 Det.A Ch1/220nm

PeakTable

DetectorACh1220nm

Peak#	Ret.Time	Area	Height	Area%	Height%
1	9.280	1275	64	0.017	0.006
2	9.630	18226	1410	0.249	0.137
3	10.054	7043339	1013066	96.227	98.187
4	10.483	130731	11564	1.786	1.121
5	10.893	27768	1924	0.379	0.186
6	11.204	36383	1410	0.497	0.137
7	12.027	61763	2330	0.844	0.226
Total		7319485	1031767	100.000	100.000

Vasopressin

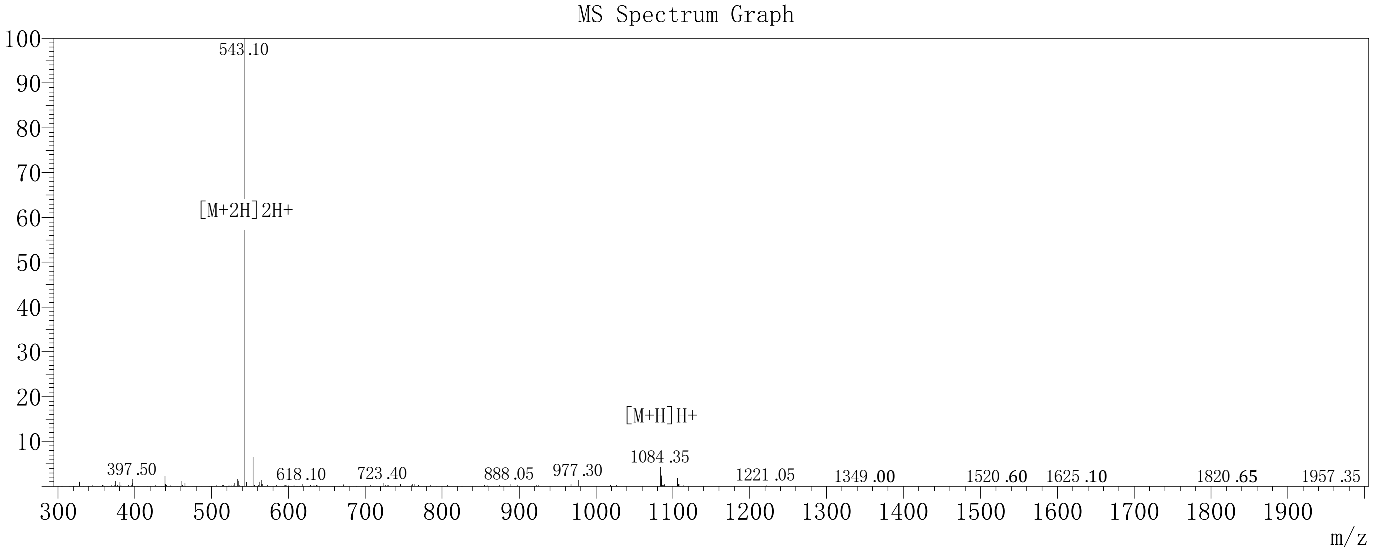

Sample Information

Dissolution method	:5%HAC+8%ACN+87%H2O	Interface	:ESI	Prerod Bias	:+1.5kv
Modified Date	:2021/10/27	Nebulizing Gas Flow	:1.50L/min	Detector	:-0.2kv
Injection Volume	:1ul	CDL Temp	:250C	T.Flow	:0.2ml/min
Heat Block Temp	:200	CDL Volt	:0v	B.conc	:50%H2O/50%MEOH

Order ID	:GP120500-3
Name	:Vasopressin
Sequence	:C*-Y-F-Q-N-C*-P-R-G-NH2
Lot.No	:GP120500-3-1015
Theoretical	:1084.25
Observed	:1084.20

Oxytocin

安徽省国平药业有限公司

CERTIFICATE OF ANALYSIS

Order ID	GP120500-4
Name	Oxytocin
Lot No.	GP120500-4-1015
Sequence	C-Y-I-Q-N-C-P-L-G*
Dissolution condition	100%H20
Length	9AA
Modification	（第一个Cys和第六个Cys之间形成二硫键，最后一个Gly酰胺化）
Molecular Weight (MW)	1007.21
Storage	-20° C

Test Items	Specifications	Results
MW by MS	1006.70	Conforms
Purityby HPLC	>95%	95.879%
Peptide Content	N/A	N/A
Moisture content	N/A	N/A
Acetic acid content	N/A	N/A
Appearance	White to off-white lyophilized powder	Conforms
Quantity	5mg	1.0mg*5

Certified by: LiuHui Date 10/28/2021

Quality Assurance Department

Note: this product is intended for research use only; not for diagnostic or human use.

Guoping Pharmaceutical Co., LTD

地址:合肥市经开区桃花工业园拓展区工投立恒工业广场A2 西F1,电话:0551-62841987 传真:0551-62841765 www.guopingyaoye.com

Oxytocin

SampleInformation

OrderID:GP120500-4
Name:Oxytocin
Sequence:C*-Y-I -Q-N-C*-P-L-G-NH2
Lot.No:GP120500-4-1015
PumpA:0.1%TriĐluoroaceticin100% water
PumpB:0.1%TriĐluoroaceticin100% acetonitrile
TotalFlow:1ml/min
Wavelength:220nm
AnalyticalcolumntypeSHIMADZUInertsilODS-SP(4.6*250mm*5um)
Dissolutionmethod:100%H2O
Inj.Volume:18uL

TimeModule	Action	Value
0.01Pumps	B.Conc	15
20.00Pumps	B.Conc	55
23.00Pumps	B.Conc	100
38.00Pumps	B.Conc	100
40.00Pumps	B.Conc	15
50.00Controller	Stop	

Chromatogram

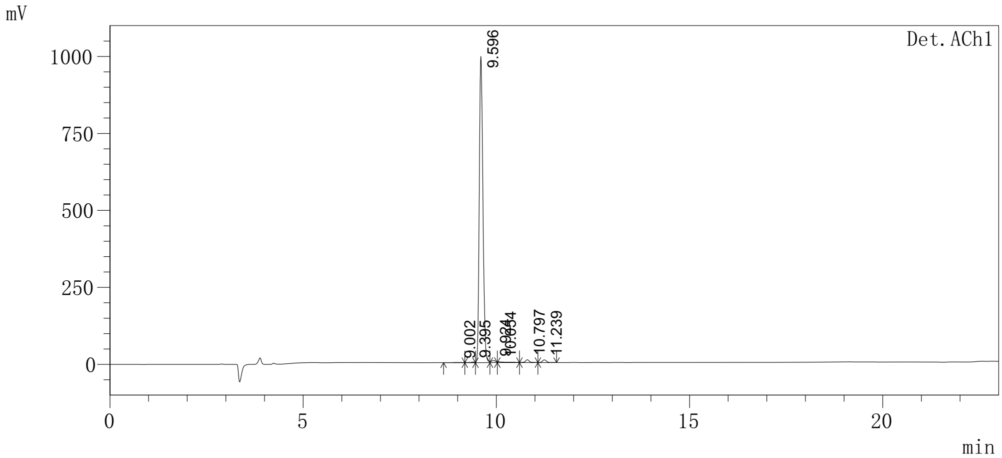

1 Det.A Ch1/220nm

PeakTable

DetectorACh1220nm

Peak#	Ret.Time	Area	Height	Area%	Height%
1	9.002	16795	787	0.249	0.077
2	9.395	17778	1820	0.263	0.178
3	9.596	6471884	994426	95.879	97.053
4	9.924	55485	7017	0.822	0.685
5	10.054	50847	2575	0.753	0.251
6	10.797	71464	9217	1.059	0.900
7	11.239	65772	8781	0.974	0.857
Total		6750026	1024623	100.000	100.000

Oxytocin

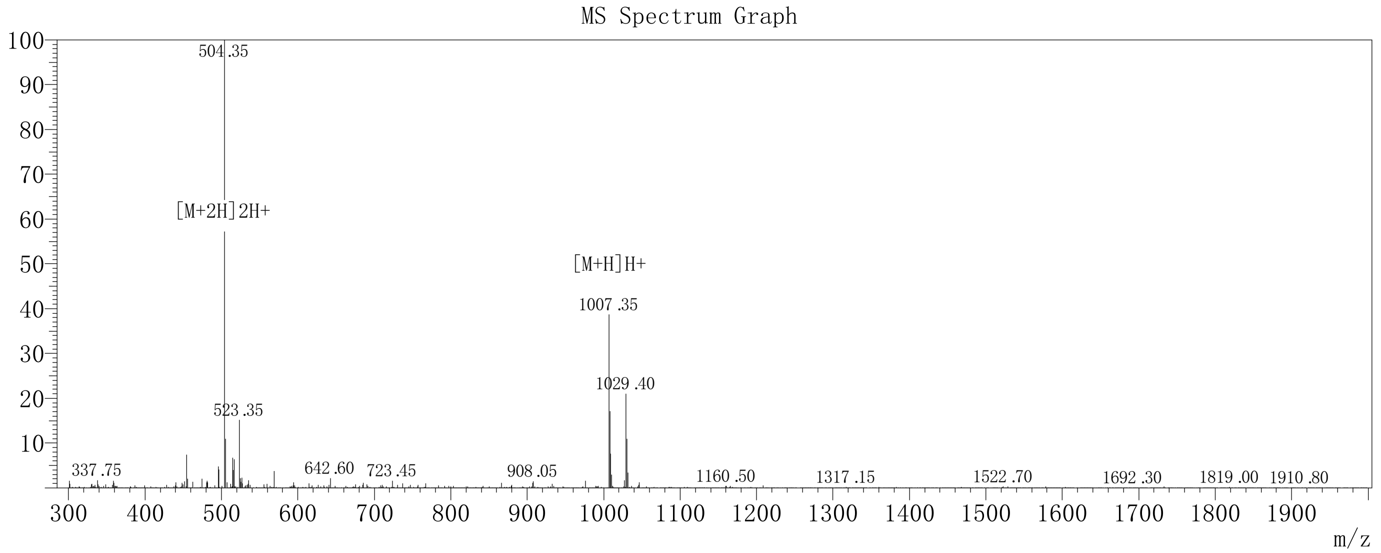

Sample Information

Dissolution method	:5%HAC+8%ACN+87%H2O	Interface	:ESI	Prerod Bias	:+1.5kv
Modified Date	:2021/10/27	Nebulizing Gas Flow	:1.50L/min	Detector	:-0.2kv
Injection Volume	:1ul	CDL Temp	:250C	T.Flow	:0.2ml/min
Heat Block Temp	:200	CDL Volt	:0v	B.conc	:50%H2O/50%MEOH

Order ID	:GP120500-4
Name	:Oxytocin
Sequence	:C*-Y-I-Q-N-C*-P-L-G-NH2
Lot.No	:GP120500-4-1015
Theoretical	:1007.21
Observed	:1006.70

Conopressin S

安徽省国平药业有限公司

CERTIFICATE OF ANALYSIS

Order ID	GP120349 -2
Name	ConopressinS
Lot No.	GP120349 -2-1124
CAS	CIIRNCPRG -NH2
Dissolutioncondition	100%H2O
Length	9AA
Modification	形成二硫键
Molecular Weight (MW)	1028.26
Storage	-20℃

Test Items	Specifications	Results
MW by MS	1027.90	Conforms
Purityby HPLC	>95%	97.370%
PeptideContent	N/A	N/A
Moisture content	N/A	N/A
Aceticacidcontent	N/A	N/A
Appearance	White tooff-whitelyophilizedpowder	Conforms
Quantity	4mg	4.0mg

Certifiedby:LiuHui Date 12/07/2020

QualityAssuranceDepartment

Note: thisproduct isintendedforresearchuse only;not fordiagnosticor human use.

Guoping PharmaceuticalCo.,LTD

地址:合肥市经开区桃花工业园拓展区工投立恒工业广场 A2 西F1,电话:0551-62841987 传真:0551-62841765 www.guopingyaoye.com

Conopressin S

SampleInformation

OrderID:GP120349-2
Name: ConopressinS
Sequence:C*IIRNC *PRG-NH2
Lot.No: GP120349-2-1124
PumpA: 0.1%TriĐluoroaceticin100%water
PumpB: 0.1%TriĐluoroaceticin100%acetonitrile
TotalFlow:1ml/min
Wavelength:220nm
Analyticalcolumntype:SHIMADZUInertsilODS-SP(4.6*250mm*5um)
Dissolutionmethod:100%H2O
Inj.Volme:12uL

Time	Module	Action	Value
0.01	Pumps	B.Conc	10
20.00	Pumps	B.Conc	50
30.00	Pumps	B.Conc	100
38.00	Pumps	B.Conc	100
40.00	Pumps	B.Conc	10
50.00	Controller	Stop	

Chromatogram

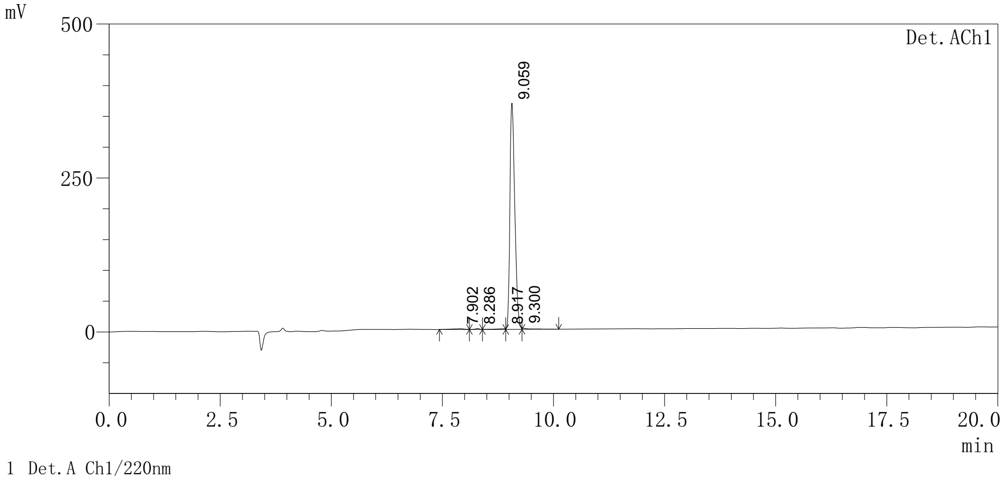

1 Det.A Ch1/220nm

PeakTable

DetectorACh1220nm

Peak#	Ret.Time	Area	Height	Area%	Height%
1	7.902	24143	1225	0.833	0.327
2	8.286	9587	1584	0.331	0.423
3	8.917	18154	1328	0.627	0.355
4	9.059	2821154	367427	97.370	98.202
5	9.300	24331	2591	0.840	0.693
Total		2897368	374154	100.000	100.000

Conopressin S

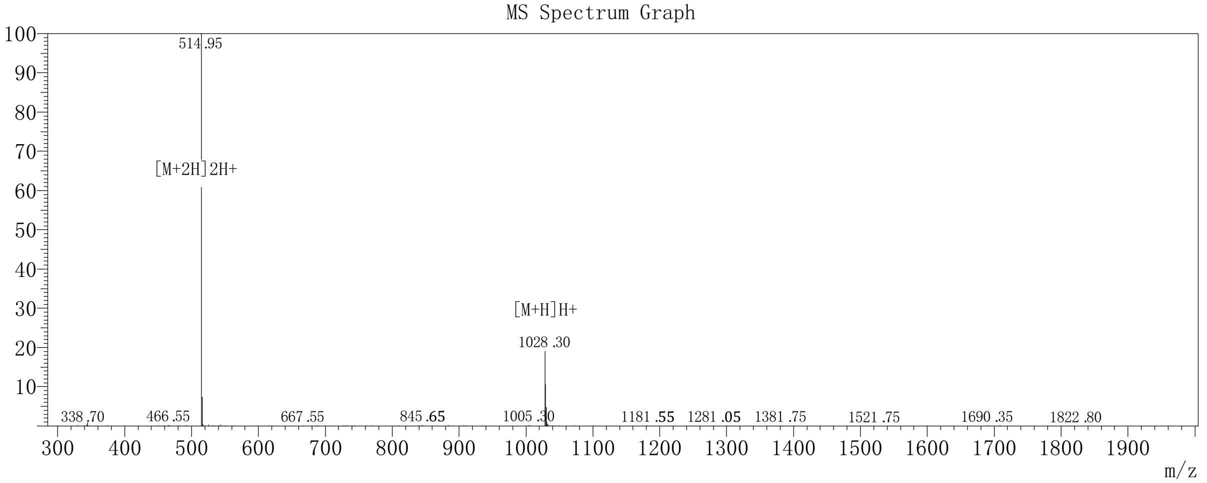

Sample Information

Dissolution method	:5%HAC+8%ACN+87%H2O	Interface	:ESI	Prerod Bias	:+1.5kv
Modified Date	:2020/12/05	Nebulizing Gas Flow	:1.50L/min	Detector	:-0.2kv
Injection Volume	:1ul	CDL Temp	:250C	T.Flow	:0.2ml/min
Heat Block Temp	:200	CDL Volt	:0v	B.conc	:50%H2O/50%MEOH

Order ID	:GP120349-2
Name	:Conopressin S
Sequence	:C*IIRNC*PRG-NH2
Lot.No	:GP120349-2-1124
Theoretical	:1028.26
Observed	:1027.90

Annetocin

安徽省国平药业有限公司

CERTIFICATE OF ANALYSIS

Order ID	GP120518-2
Name	Annetocin
Lot No.	GP120518-2-1109
Sequence	C-F-V-R-N-C-P-T-G*
Dissolution condition	100%H2O
Length	9AA
Modification	半胱氨酸之间形成二硫键，末端甘氨酸发生酰胺化
Molecular Weight (MW)	993.16
Storage	-20° C

Test Items	Specifications	Results
MW by MS	992.70	Conforms
Purityby HPLC	>95%	97.780%
Peptide Content	N/A	N/A
Moisture content	N/A	N/A
Acetic acid content	N/A	N/A
Appearance	White to off-white lyophilized powder	Conforms
Quantity	5mg	1.0mg*5

Certified by: LiuHui   Date 11/17/2021

Quality Assurance Department

Note: this product is intended for research use only; not for diagnostic or human use.

Guoping Pharmaceutical Co., LTD

地址:合肥市经开区桃花工业园拓展区工投立恒工业广场A2 西F1,电话:0551-62841987 传真:0551-62841765 www.guopingyaoye.com

Annetocin

SampleInformation

OrderID :GP120518-2
Name :Annetocin
Sequence :C*-F-VR-N-C*-PT-G-NH2
Lot.No :GP120518-2-1109
PumpA :0.1%TriĐluoroaceticin100% atwer
PumpB :0.1%TriĐluoroaceticin100% cetaonitrile
TotalFlow :1ml/min
Wavelength :220nm
AnalyticalcolumntypeSHIMADZUInertsilODS-SP(4.6*250mm*5um)
Dissolutionmethod:100%H2O
Inj.Volume :10uL

Time	Module	Action	Value
0.01	Pumps	B.Conc	10
20.00	Pumps	B.Conc	50
23.00	Pumps	B.Conc	100
38.00	Pumps	B.Conc	100
40.00	Pumps	B.Conc	10
50.00	Controller	Stop	

Chromatogram

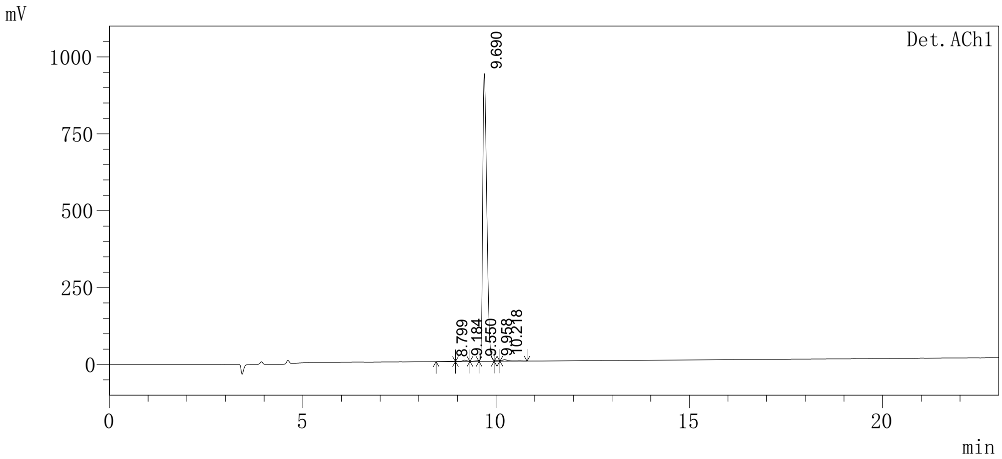

1 Det.A Ch1/220nm

PeakTable

DetectorACh1220nm

Peak#	Ret.Time	Area	Height	Area%	Height%
1	8.799	15728	1531	0.224	0.161
2	9.184	34860	4258	0.497	0.447
3	9.550	18528	2623	0.264	0.275
4	9.690	6862469	935969	97.780	98.261
5	9.958	22217	3249	0.317	0.341
6	10.218	64471	4906	0.919	0.515
Total		7018272	952537	100.000	100.000

Annetocin

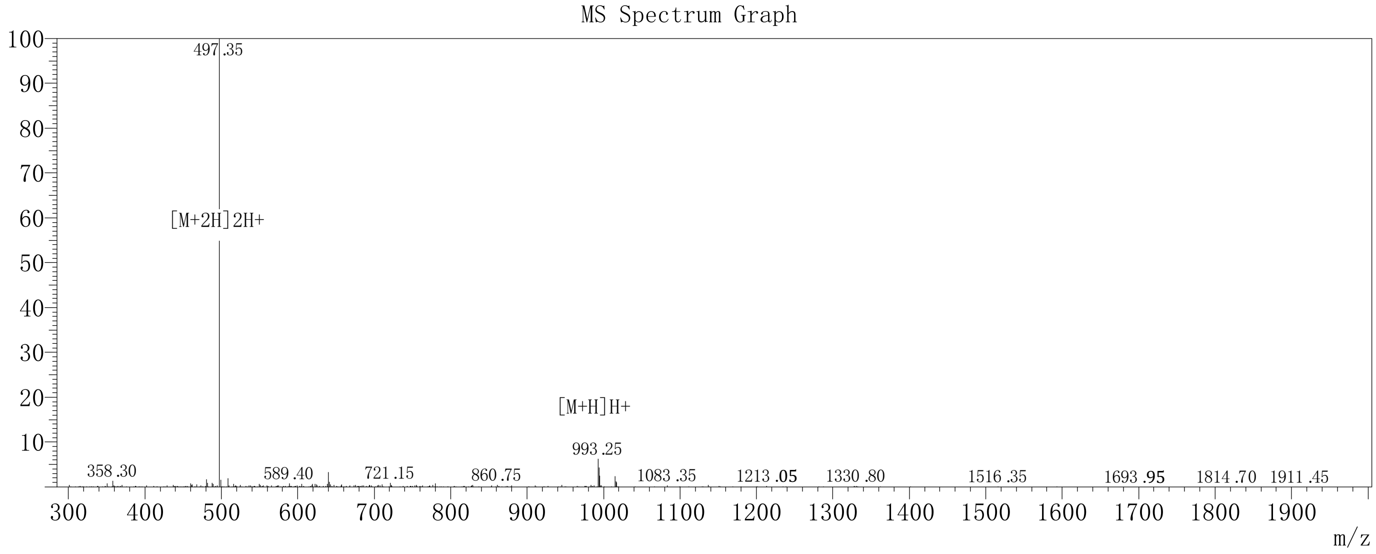

Sample Information

Dissolution method	:5%HAC+8%ACN+87%H2O	Interface	:ESI	Prerod Bias	:+1.5kv
Modified Date	:2021/11/16	Nebulizing Gas Flow	:1.50L/min	Detector	:-0.2kv
Injection Volume	:1ul	CDL Temp	:250C	T.Flow	:0.2ml/min
Heat Block Temp	:200	CDL Volt	:0v	B.conc	:50%H2O/50%MEOH

Order ID	:GP120518-2
Name	:Annetocin
Sequence	:C*-F-V-R-N-C*-P-T-G-NH2
Lot.No	:GP120518-2-11109
Theoretical	:993.16
Observed	:992.70

apVT

安徽省国平药业有限公司

CERTIFICATE OF ANALYSIS

Order ID	GP120500-1
Name	apVT'
Lot No.	GP120500-1-1015
Sequence	C-F-I-R-N-C-P-K-G*
Dissolution condition	100%H2O
Length	9AA
Modification	（最后一个Gly酰胺化）
Molecular Weight (MW)	1036.28
Storage	-20° C

Test Items	Specifications	Results
MW by MS	1036.10	Conforms
Purity by HPLC	>95%	97.537%
Peptide Content	N/A	N/A
Moisture content	N/A	N/A
Acetic acid content	N/A	N/A
Appearance	White to off-white lyophilized powder	Conforms
Quantity	5mg	1.0mg*5

Certified by: LiuHui Date 10/28/2021

Quality Assurance Department

Note: this product is intended for research use only; not for diagnostic or human use.

Guoping Pharmaceutical Co., LTD

地址:合肥市经开区桃花工业园拓展区工投立恒工业广场A2 西F1,电话:0551-62841987 传真:0551-62841765 www.guopingyaoye.com

apVT

SampleInformation

OrderID	:GP120500-1
Name	:apVT
Sequence	:C-F-I-R-N-C-P-K-G-NH2
Lot.No	:GP120500-1-1015
PumpA	:0.1%TriÐluoroaceticin100% water
PumpB	:0.1%TriÐluoroaceticin100% cetaonitrile
TotalFlow	:1ml/min
Wavelength	:220nm
Analyticalcolumntype	:SHIMADZUInertsilODS-SP(4.6*250mm*5um)
Dissolutionmethod	:100%H2O
Inj.Volume	:13uL

Time	Module	Action	Value
0.01	Pumps	B.Conc	5
20.00	Pumps	B.Conc	45
23.00	Pumps	B.Conc	100
38.00	Pumps	B.Conc	100
40.00	Pumps	B.Conc	5
50.00	Controller	Stop	

Chromatogram

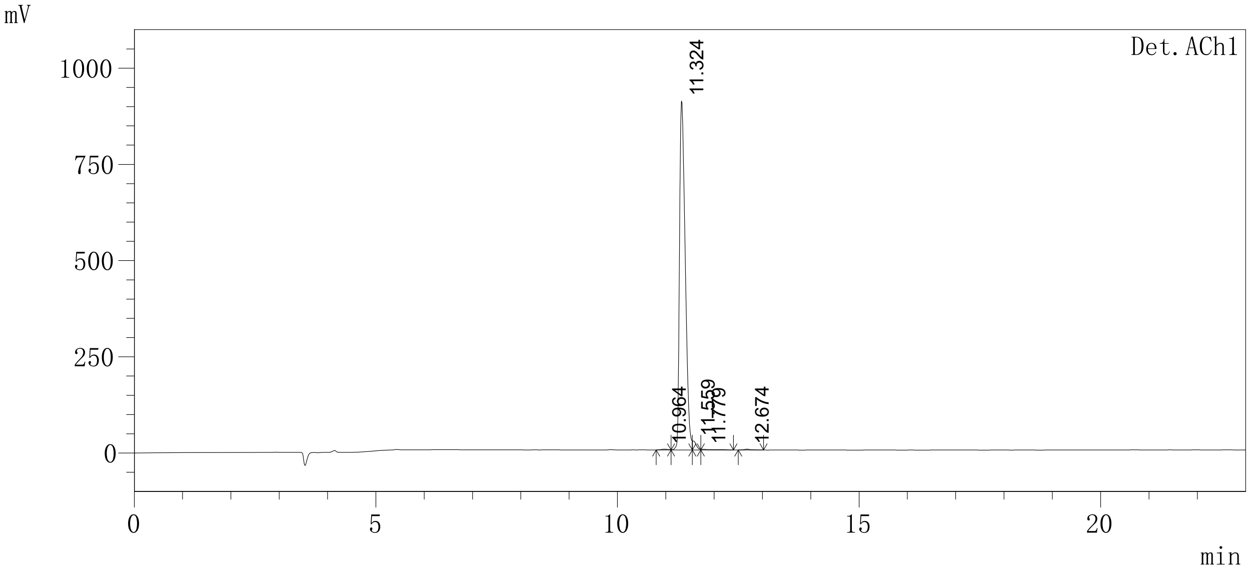

1 Det.A Ch1/220nm

PeakTable

DetectorACh1220nm

Peak#	Ret.Time	Area	Height	Area%	Height%
1	10.964	20417	1970	0.265	0.211
2	11.324	7504539	906491	97.537	96.880
3	11.559	122611	23385	1.594	2.499
4	11.779	29104	1653	0.378	0.177
5	12.674	17369	2189	0.226	0.234
Total		7694039	935689	100.000	100.000

apVT

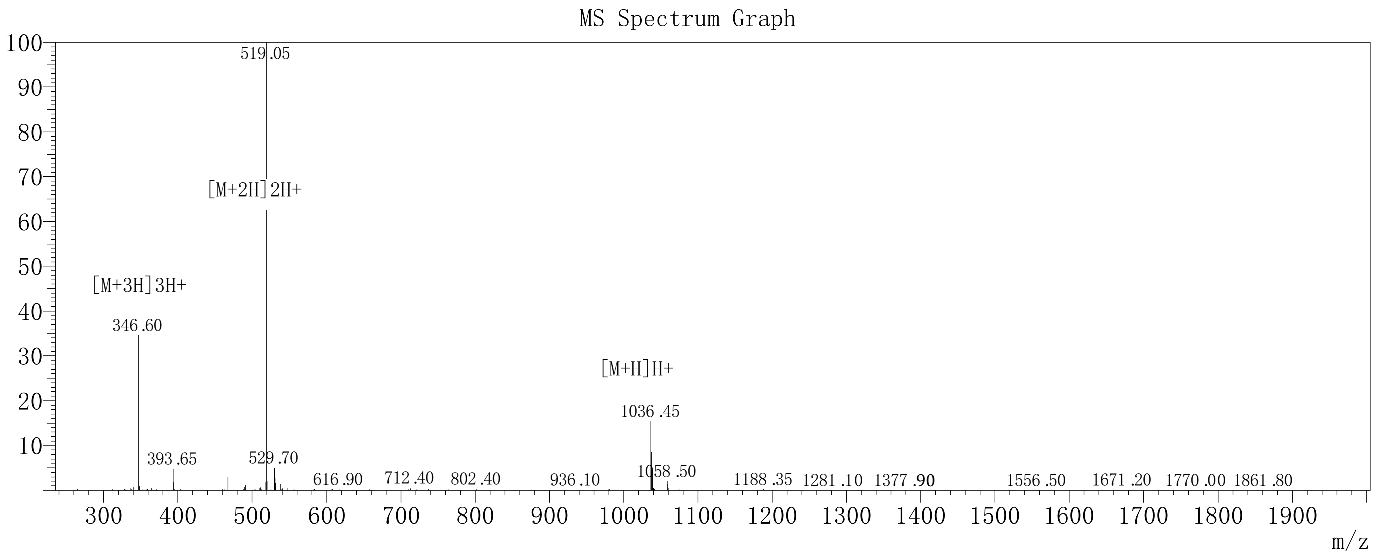

Sample Information

Dissolution method	:5%HAC+8%ACN+87%H2O	Interface	:ESI	Prerod Bias	:+1.5kv
Modified Date	:2021/10/26	Nebulizing Gas Flow	:1.50L/min	Detector	:-0.2kv
Injection Volume	:1ul	CDL Temp	:250C	T.Flow	:0.2ml/min
Heat Block Temp	:200	CDL Volt	:0v	B.conc	:50%H2O/50%MEOH

Order ID	:GP120500-1
Name	:apVT'
Sequence	:C-F-I-R-N-C-P-K-G*
Lot.No	:GP120500-1-1015
Theoretical	:1036.28
Observed	:1036.10

[Cys(Acm)[1]]apVT

安徽省国平药业有限公司

CERTIFICATE OF ANALYSIS

Order ID	GP120556-1
Name	[Cys(Acm)[1]]apVT
Lot No.	GP120556-1-0104
Sequence	C（Acm）-F-I-R-N-C-P-K-G*
Dissolution condition	100%H2O
Length	9AA
Modification	末端酰胺化
Molecular Weight (MW)	1107.28
Storage	-20°C

Test Items	Specifications	Results
MW by MS	1106.85	Conforms
Purityby HPLC	>95%	98.365%
Peptide Content	N/A	N/A
Moisture content	N/A	N/A
Acetic acid content	N/A	N/A
Appearance	White to off-white lyophilized powder	Conforms
Quantity	5mg	1.0mg*5

Certified by: LiuHui   Date 02/16/2022

Quality Assurance Department

Note: this product is intended for research use only; not for diagnostic or **hu**man use.

Guoping Pharmaceutical Co., LTD

地址:合肥市经开区桃花工业园拓展区工投立恒工业广场A2 西F1,电话:0551-62841987 传真:0551-62841765 www.guopingyaoye.com

[Cys(Acm)^{1}]apVT

SampleInformation

OrderID:GP120556-1
Name: [Cys(Acm)^{1}]apVT
Sequence:C（Acm）-F-I-R-N-C-P-K-G*-NH2
Lot.No: GP120556-1-0104
PumpA: 0.1%TriĐluoroaceticin100%water
PumpB: 0.1%TriĐluoroaceticin100%acetonitrile
TotalFlow:1ml/min
Wavelength:220nm
Analyticalcolumntype:SHIMADZUInertsilODS-SP(4.6*250mm*5um)
Dissolutionmethod:100%H2O
Inj.Volme:15uL

TimeModule	Action	Value
0.01Pumps	B.Conc	10
20.00Pumps	B.Conc	50
23.00Pumps	B.Conc	100
38.00Pumps	B.Conc	100
40.00Pumps	B.Conc	10
50.00Controller	Stop	

Chromatogram

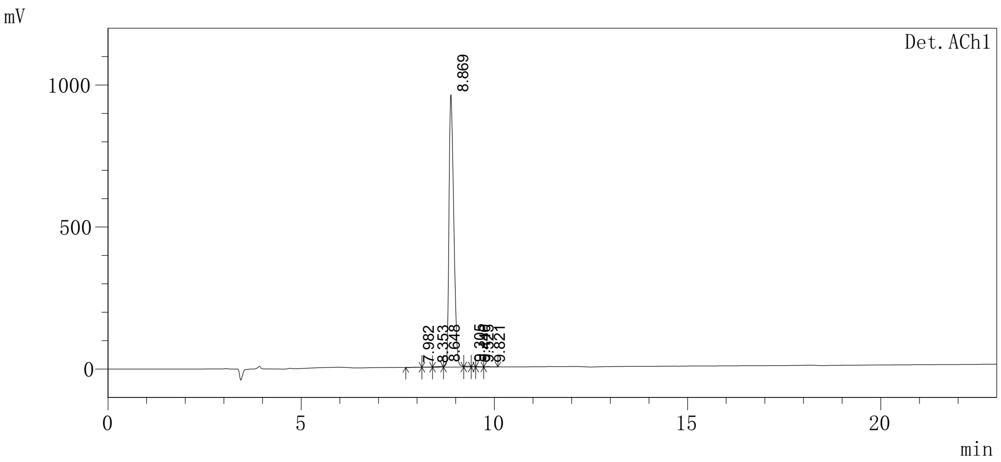

1 Det.A Ch1/220nm

PeakTable

DetectorACh1220nm

Peak#	Ret.Time	Area	Height	Area%	Height%
1	7.982	9072	790	0.109	0.081
2	8.353	11160	1011	0.134	0.104
3	8.648	38551	3299	0.463	0.339
4	8.869	8197889	959383	98.365	98.574
5	9.305	33861	3349	0.406	0.344
6	9.440	15072	2381	0.181	0.245
7	9.525	13939	1704	0.167	0.175
8	9.821	14651	1343	0.176	0.138
Total		8334194	973260	100.000	100.000

[Cys(Acm)[1]]apVT

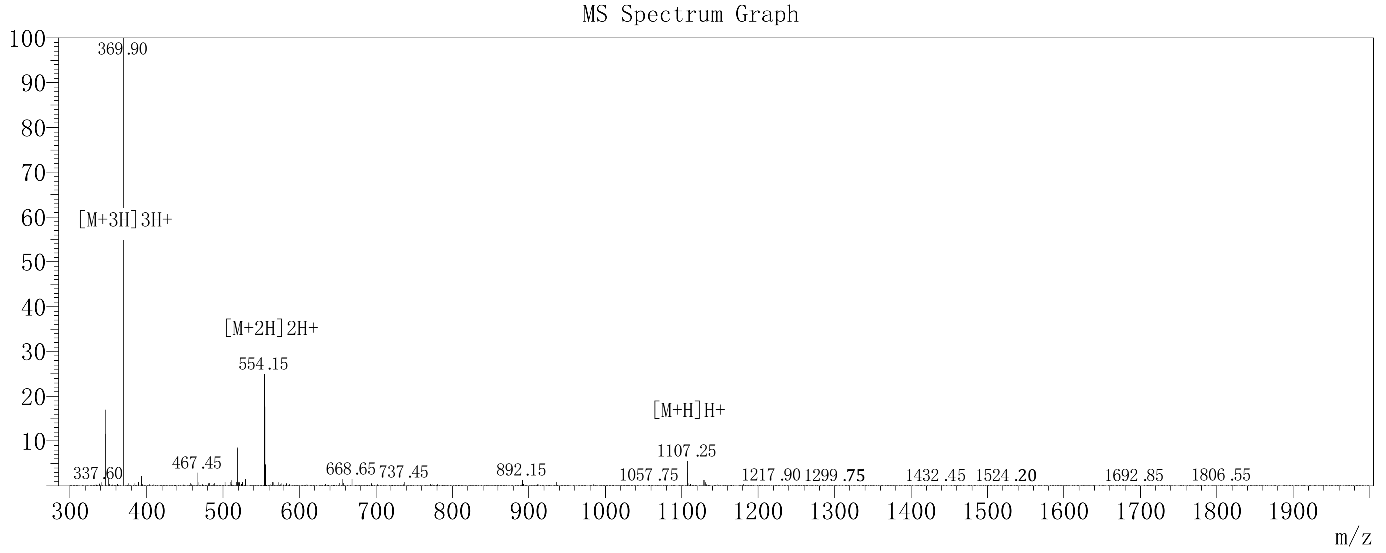

Sample Information

Dissolution method	:5%HAC+8%ACN+87%H2O	Interface	:ESI	Prerod Bias	:+1.5kv
Modified Date	:2022/01/19	Nebulizing Gas Flow	:1.50L/min	Detector	:-0.2kv
Injection Volume	:1ul	CDL Temp	:250C	T.Flow	:0.2ml/min
Heat Block Temp	:200	CDL Volt	:0v	B.conc	:50%H2O/50%MEOH

Order ID	:GP120556-1
Name	:[Cys(Acm)[1]]apVT
Sequence	:C (Acm) -F-I-R-N-C-P-K-G-NH2
Lot.No	:GP120556-1-0104
Theoretical	:1107.28
Observed	:1106.70

[Cys(Acm)^6]apVT

安徽省国平药业有限公司

CERTIFICATE OF ANALYSIS

Order ID	GP120556-2
Name	[Cys(Acm)^6]apVT
Lot No.	GP120556-2-0104
Sequence	C-F-I-R-N-C（Acm)-P-K-G*
Dissolution condition	100%H2O
Length	9AA
Modification	末端酰胺化
Molecular Weight (MW)	1107.28
Storage	-20° C

Test Items	Specifications	Results
MW by MS	1107.00	Conforms
Purityby HPLC	>95%	96.217%
Peptide Content	N/A	N/A
Moisture content	N/A	N/A
Acetic acid content	N/A	N/A
Appearance	White to off-white lyophilized powder	Conforms
Quantity	5mg	1.0mg*5

Certified by: LiuHui　　Date 02/16/2022

Quality Assurance Department

Note: this product is intended for research use only; not for diagnostic or human use.

Guoping Pharmaceutical Co., LTD

地址:合肥市经开区桃花工业园拓展区工投立恒工业广场A2 西F1,电话:0551-62841987 传真:0551-62841765 www.guopingyaoye.com

[Cys(Acm)^{6}]apVT

SampleInformation

OrderID:GP120556-2
Name: [Cys(Acm)^{6}]apVT
Sequence:C-F-I-R-N-C(Acm)-P-K-G*-NH2
Lot.No: GP120556-2-0104
PumpA: 0.1%TriÐluoroaceticin100%water
PumpB: 0.1%TriÐluoroaceticin100%acetonitrile
TotalFlow:1ml/min
Wavelength:220nm
Analyticalcolumntype:SHIMADZUInertsilODS-SP(4.6*250mm*5um)
Dissolutionmethod:100%H2O
Inj.Volme:14uL

Time	Module	Action	Value
0.01	Pumps	B.Conc	10
20.00	Pumps	B.Conc	50
23.00	Pumps	B.Conc	100
38.00	Pumps	B.Conc	100
40.00	Pumps	B.Conc	10
50.00	Controller	Stop	

Chromatogram

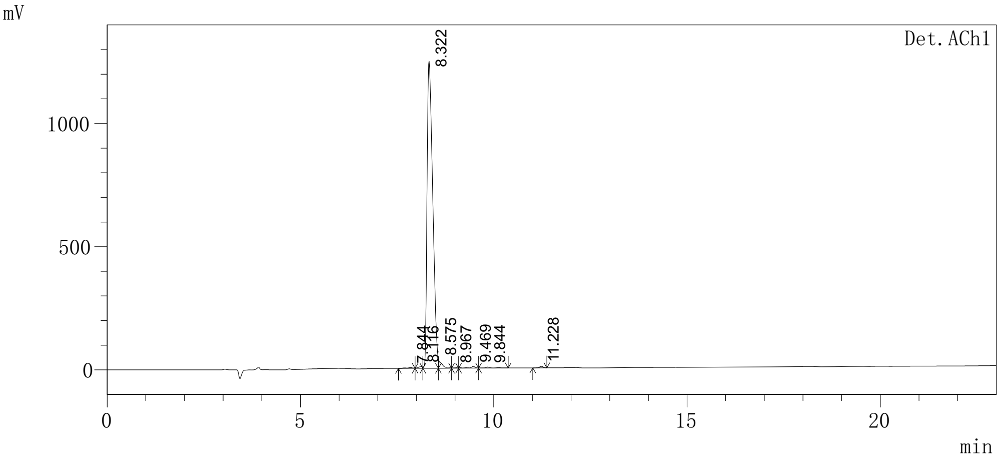

1 Det.A Ch1/220nm

PeakTable

DetectorACh1220nm

Peak#	Ret.Time	Area	Height	Area%	Height%
1	7.844	40072	3158	0.304	0.241
2	8.116	50432	6213	0.383	0.474
3	8.322	12668991	1247303	96.217	95.116
4	8.575	210911	34325	1.602	2.618
5	8.967	30243	3707	0.230	0.283
6	9.469	83718	6648	0.636	0.507
7	9.844	42444	3946	0.322	0.301
8	11.228	40270	6047	0.306	0.461
Total		13167081	1311348	100.000	100.000

[Cys(Acm)⁶]apVT

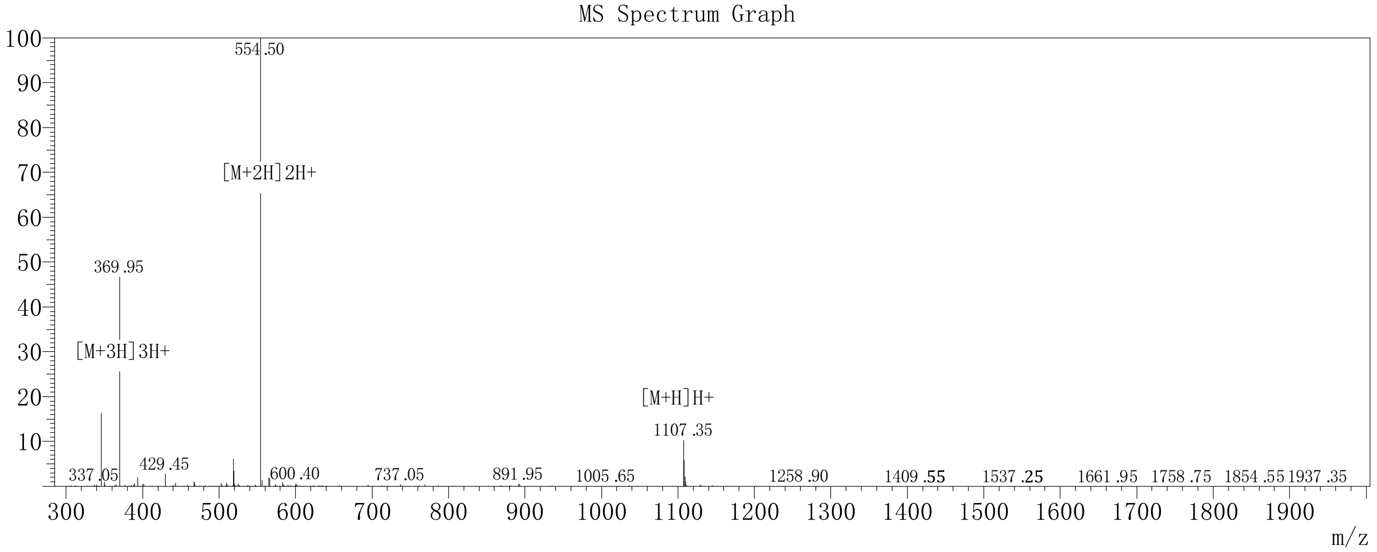

Sample Information

Dissolution method	:5%HAC+8%ACN+87%H20	Interface	:ESI	Prerod Bias	:+1.5kv
Modified Date	:2022/01/19	Nebulizing Gas Flow	:1.50L/min	Detector	:-0.2kv
Injection Volume	:1ul	CDL Temp	:250C	T.Flow	:0.2ml/min
Heat Block Temp	:200	CDL Volt	:0v	B.conc	:50%H2O/50%MEOH

Order ID	:GP120556-2
Name	:[Cys(Acm)⁶]apVT
Sequence	:C-F-I-R-N-C（Acm-P-K-G-NH2
Lot.No	:GP120556-2-0104
Theoretical	:1107.28
Observed	:1107.00

[Cys(Acm)^{1,6}]apVT

安徽省国平药业有限公司

CERTIFICATE OF ANALYSIS

Order ID	GP120518-3
Name	[Cys(Acm)^{1,6}]apVT
Lot No.	GP120518-3-1109
Sequence	C(Acm)-F-I-R-N-C(Acm)-P-K-G*
Dissolution condition	100%H2O
Length	9AA
Modification	末端甘氨酸发生酰胺化，把两个半胱氨酸的巯基保护起来
Molecular Weight (MW)	1178.28
Storage	-20° C

Test Items	Specifications	Results
MW by MS	1178.00	Conforms
Purityby HPLC	>95%	97.432%
Peptide Content	N/A	N/A
Moisture content	N/A	N/A
Acetic acid content	N/A	N/A
Appearance	White to off-white lyophilized powder	Conforms
Quantity	5mg	1.0mg*5

Certified by: LiuHui Date 11/17/2021

Quality Assurance Department

Note: this product is intended for research use only; not for diagnostic or human use.

Guoping Pharmaceutical Co., LTD

地址:合肥市经开区桃花工业园拓展区工投立恒工业广场A2 西F1,电话:0551-62841987 传真:0551-62841765 www.guopingyaoye.com

[Cys(Acm)[1,6]]apVT

SampleInformation

OrderID:GP120518-3
Name: [Cys(Acm)[1,6]]apVT
Sequence: C(Acm)-F-I-R-N-C(Acm)-K-G*
Lot.No: GP120518-3-1109
PumpA: 0.1%TriĐluoroaceticin100%water
PumpB: 0.1%TriĐluoroaceticin100%acetonitrile
TotalFlow:1ml/min
Wavelength:220nm
Analyticalcolumntype:SHIMADZUInertsilODS-SP(4.6*250mm*5um)
Dissolutionmethod:100%H2O
Inj.Volme:11uL

Time	Module	Action	Value
0.01	Pumps	B.Conc	10
20.00	Pumps	B.Conc	50
23.00	Pumps	B.Conc	100
38.00	Pumps	B.Conc	100
40.00	Pumps	B.Conc	10
50.00	Controller	Stop	

Chromatogram

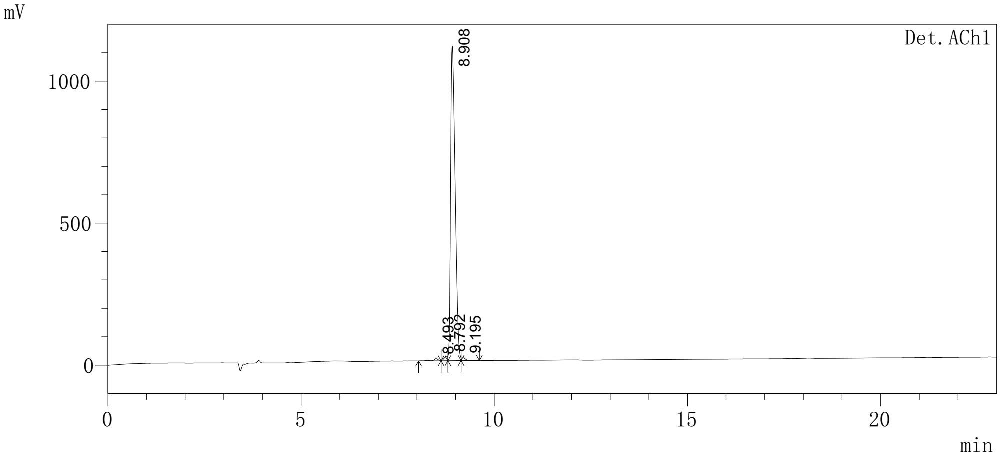

1 Det.A Ch1/220nm

PeakTable

DetectorACh1220nm

Peak#	Ret.Time	Area	Height	Area%	Height%
1	8.493	66380	7580	0.712	0.664
2	8.792	98235	15733	1.054	1.377
3	8.908	9077696	1108818	97.432	97.063
4	9.195	74617	10241	0.801	0.896
Total		9316928	1142372	100.000	100.000

[Cys(Acm)[1,6]]apVT

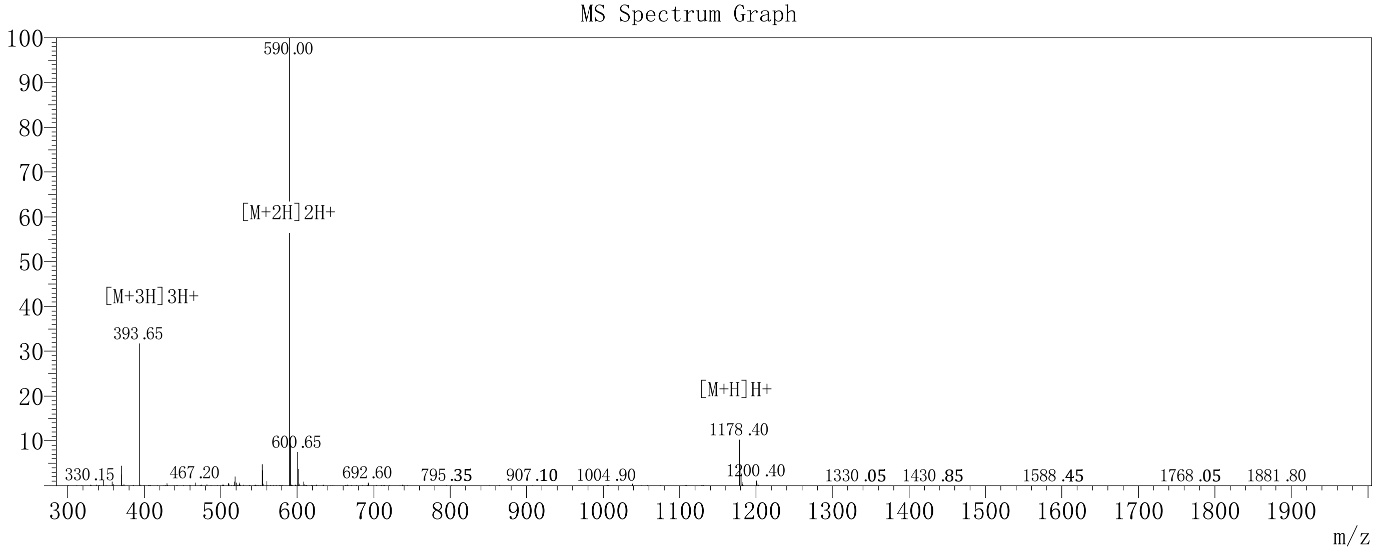

Sample Information

Dissolution method	:5%HAC+8%ACN+87%H2O	Interface	:ESI	Prerod Bias	:+1.5kv
Modified Date	:2021/11/16	Nebulizing Gas Flow	:1.50L/min	Detector	:-0.2kv
Injection Volume	:1ul	CDL Temp	:250C	T.Flow	:0.2ml/min
Heat Block Temp	:200	CDL Volt	:0v	B.conc	:50%H2O/50%MEOH

Order ID	:GP120518-3
Name	:[Cys(Acm)[1,6]]apVT
Sequence	:C(Acm)-F-I-R-N-C(Acm)-P-K-G-NH2
Lot.No	:GP120518-3-1109
Theoretical	:1178.28
Observed	:1178.00

[Ser^{1,6}]apVT

安徽省国平药业有限公司

CERTIFICATE OF ANALYSIS

**Order ID**	GP120556-3
**Name**	[Ser^{1,6}]apVT
**Lot No.**	GP120556-3-0104
**Sequence**	S-F-I-R-N-S-P-K-G*
**Dissolution condition**	100%H2O
**Length**	9AA
**Modification**	末端酰胺化
**Molecular Weight (MW)**	1004.14
**Storage**	20°C

**Test Items**	**Specifications**	**Results**
**MW by MS**	1004.00	Conforms
**Purity by HPLC**	>95%	97.185%
**Peptide Content**	N/A	N/A
**Moisture content**	N/A	N/A
**Acetic acid content**	N/A	N/A
**Appearance**	White to off-white lyophilized powder	Conforms
**Quantity**	5mg	1.0mg*5

Certified by: LiuHui Date 02/16/2022

Quality Assurance Department

Note: this product is intended for research use only; not for diagnostic or human use.

Guoping Pharmaceutical Co., LTD

地址:合肥市经开区桃花工业园拓展区工投立恒工业广场A2西F1,电话:0551-62841987 传真:0551-62841765 www.guopingyaoye.com

[Ser^{1,6}]apVT

Sample Information

Order ID :GP120556-3
Name :[Ser1,6]apVT
Sequence :S-F-I-R-N-S-P-K-G*-NH2
Lot.No :GP120556-3-0104
Pump A :0.1%Trifluoroacetic in 100% water
Pump B :0.1%Trifluoroacetic in 100% acetonitrile
Total Flow :1ml/min
Wavelength :220nm
Analytical column type :SHIMADZU Inertsil ODS-SP(4.6*250mm*5um)
Dissolution method :100%H2O
Inj. Volume :13 uL

Time	Module	Action	Value
0.01	Pumps	B.Conc	5
20.00	Pumps	B.Conc	45
23.00	Pumps	B.Conc	100
38.00	Pumps	B.Conc	100
40.00	Pumps	B.Conc	5
50.00	Controller	Stop	

Chromatogram

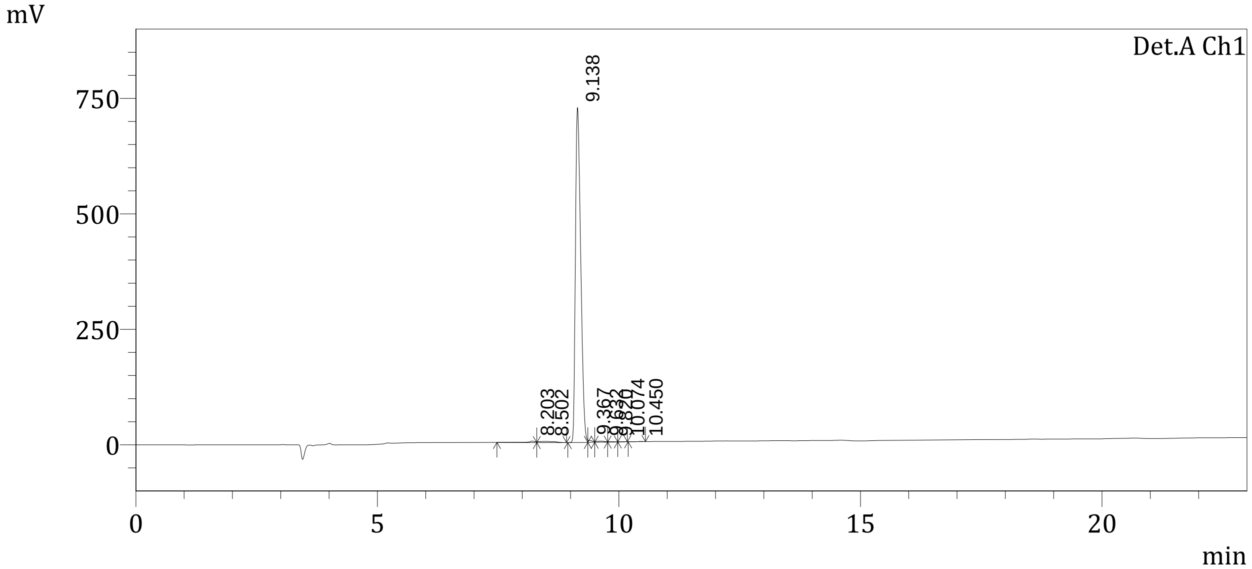

1 Det.A Ch1/220nm

PeakTable

Detector A Ch1 220nm

Peak#	Ret. Time	Area	Height	Area %	Height %
1	8.203	36596	3020	0.644	0.409
2	8.502	57795	2406	1.016	0.326
3	9.138	5525806	725179	97.185	98.137
4	9.367	25662	4348	0.451	0.588
5	9.632	27033	2336	0.475	0.316
6	9.820	5938	733	0.104	0.099
7	10.074	3041	485	0.053	0.066
8	10.450	4008	440	0.070	0.060
Total		5685879	738947	100.000	100.000

[Ser^{1,6}]apVT

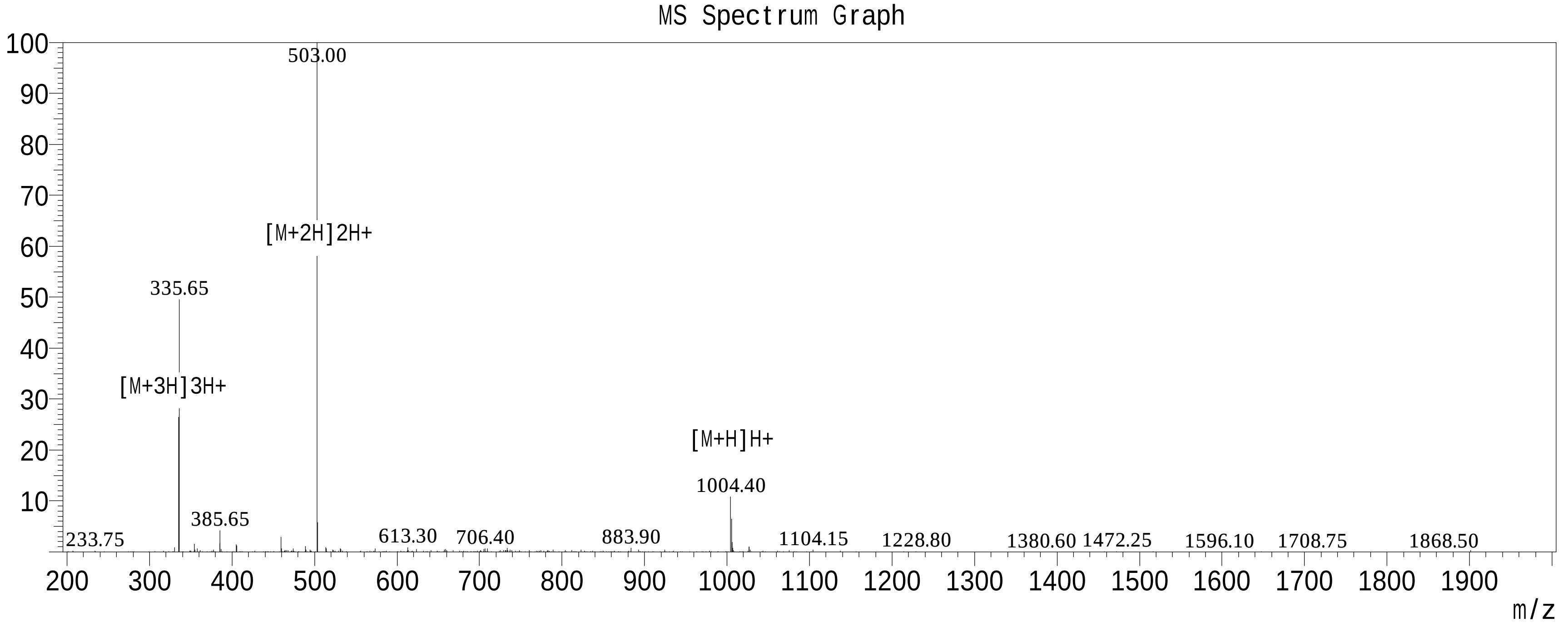

Sample Information

Dissolution method	:5%HAC+8%ACN+87%H2O	Interface	:ESI	Prerod Bias	:+1.5kv
Modified Date	:2022/01/19	Nebulizing Gas Flow	:1.50L/min	Detector	:-0.2kv
Injection Volume	:1ul	CDL Temp	:250C	T.Flow	:0.2ml/min
Heat Block Temp	:200	CDL Volt	:0v	B.conc	:50%H2O/50%MEOH

Order ID	:GP120556-3
Name	:[Ser1,6]apVT
Sequence	:S-F-I-R-N-S-P-K-G-NH2
Lot.No	:GP120556-3-0104
Theoretical	:1004.14
Observed	:1004.00

apVT

安徽省国平药业有限公司

CERTIFICATE OF ANALYSIS

Order ID	GP120500-2
Name	apVT-OH
Lot No.	GP120500-2-1015
Sequence	C-F-I-R-N-C-P-K-G
Dissolution condition	100%H2O
Length	9AA
Modification	（第一个Cys和第六个Cys之间形成二硫键）
MolecularWeight (MW)	1035.27
Storage	-20°C

Test Items	Specifications	Results
MW by MS	1035.00	Conforms
Purityby HPLC	>95%	95.046%
Peptide Content	N/A	N/A
Moisture content	N/A	N/A
Acetic acid content	N/A	N/A
Appearance	White to off-white lyophilized powder	Conforms
Quantity	5mg	1.0mg*5

Certified by: LiuHui　　Date 10/28/2021

Quality Assurance Department

Note: this product is intended for research use only; not for diagnostic or **hu**man use**.**

Guoping Pharmaceutical Co., LTD

地址:合肥市经开区桃花工业园拓展区工投立恒工业广场A2 西F1,电话:0551-62841987 传真:0551-62841765 www.guopingyaoye.com

apVT

SampleInformation

OrderID:GP120500-2
Name: apVT-OH
Sequence:C*-F-I-R-N-C*-P-K-G
Lot.No: GP120500-2-1015
PumpA: 0.1%TriÐluoroaceticin100%water
PumpB: 0.1%TriÐluoroaceticin100%acetonitrile
TotalFlow:1ml/min
Wavelength:220nm
Analyticalcolumntype:SHIMADZUInertsilODS-SP(4.6*250mm*5um)
Dissolutionmethod:100%H2O
Inj.Volme:15uL

Time	Module	Action	Value
0.01	Pumps	B.Conc	10
20.00	Pumps	B.Conc	50
23.00	Pumps	B.Conc	100
38.00	Pumps	B.Conc	100
40.00	Pumps	B.Conc	10
50.00	Controller	Stop	

Chromatogram

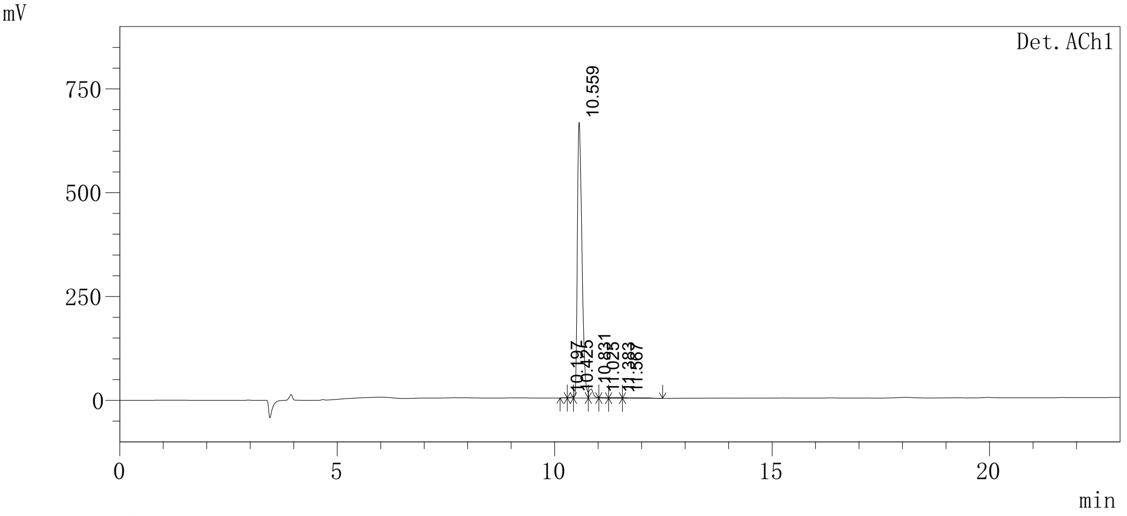

1 Det.A Ch1/220nm

PeakTable

DetectorACh1220nm

Peak#	Ret.Time	Area	Height	Area%	Height%
1	10.197	4581	672	0.089	0.097
2	10.425	16949	3495	0.329	0.504
3	10.559	4891803	663301	95.046	95.625
4	10.831	149943	22041	2.913	3.177
5	11.025	17910	1538	0.348	0.222
6	11.383	25388	1512	0.493	0.218
7	11.567	40180	1090	0.781	0.157
Total		5146754	693648	100.000	100.000

apVT

MS Spectrum Graph

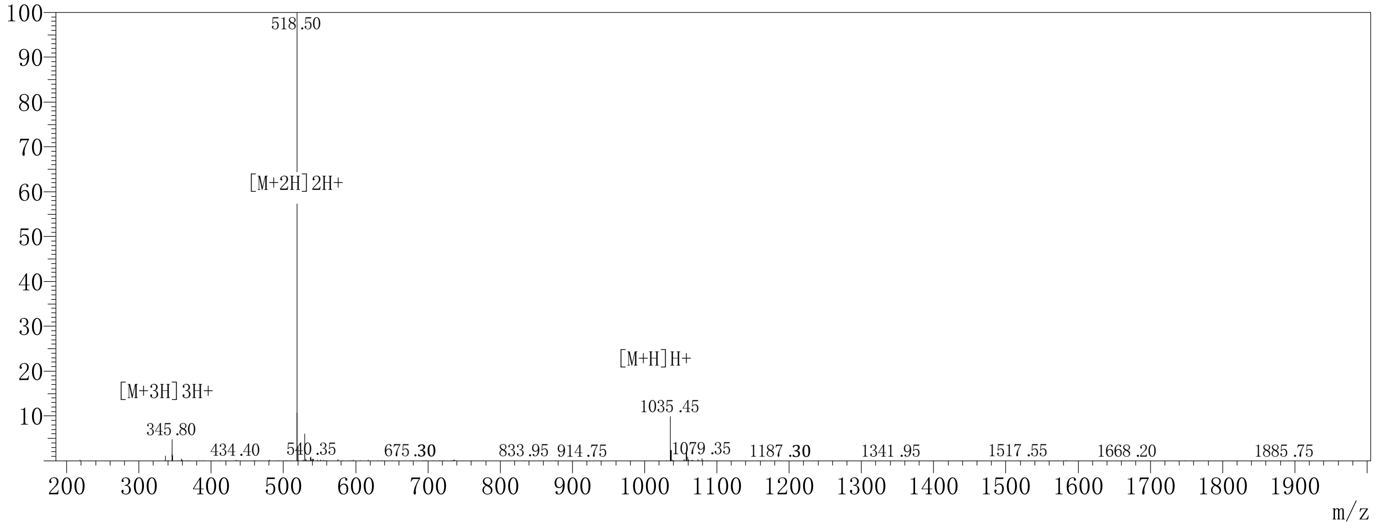

Sample Information

Dissolution method	:5%HAC+8%ACN+87%H2O	Interface	:ESI	Prerod Bias	:+1.5kv
Modified Date	:2021/10/26	Nebulizing Gas Flow	:1.50L/min	Detector	:-0.2kv
Injection Volume	:1ul	CDL Temp	:250C	T.Flow	:0.2ml/min
Heat Block Temp	:200	CDL Volt	:0v	B.conc	:50%H2O/50%MEOH

Order ID	:GP120500-2
Name	:apVT-OH
Sequence	:C*-F-I-R-N-C*-P-K-G
Lot.No	:GP120500-2-1015
Theoretical	:1035.27
Observed	:1035.00

apVT

安徽省国平药业有限公司

CERTIFICATE OF ANALYSIS

Order **ID**	GP120518-1
Name	apVT'-OH
Lot No**.**	GP120518-1-1109
Sequence	C-F-I-R-N-C-P-K-G
**Diss**olution condition	100%H2O
Length	9AA
Modification	半胱氨酸之间不形成二硫键，末端甘氨酸不发生酰胺化
Molecular Weight (MW)	1037.27
Stor**age**	-20° C

Tes**t Items**	Specifications	**Results**
MW by MS	1037.00	Conforms
Purityby HPLC	>95%	97.485%
Peptide Conte**n**t	N/A	N/A
Moisture conte**n**t	N/A	N/A
Acetic acid **content**	N/A	N/A
Appearance	White to off-white lyophilized powder	Conforms
Quan**tity**	5mg	1.0mg*5

Certified by: LiuHui　　　　Date 11/17/2021

Quality Assurance Department

Note: this product is intended for research use only; not for diagnostic or **hu**man use**.**

Guoping Pharmaceutical Co., LTD

地址:合肥市经开区桃花工业园拓展区工投立恒工业广场A2 西F1,电话:0551-62841987 传真:0551-62841765 www.guopingyaoye.com

apVT

SampleInformation

OrderID :GP120518-1
Name : apVT'-OH
Sequence :C-F-IR-N-C-P-K-G
Lot.No :GP120518-1-1109
PumpA :0.1%TriĐluoroaceticin100%water
PumpB :0.1%TriĐluoroaceticin100%acetonitrile
TotalFlow :1ml/min
Wavelength :220nm
Analyticalcolumntype:SHIMADZUInertsilODS-SP(4.6*250mm*5um)
Dissolutionmethod:100%H2O
Inj.Volme :8uL

Time	Module	Action	Value
0.01	Pumps	B.Conc	10
20.00	Pumps	B.Conc	50
23.00	Pumps	B.Conc	100
38.00	Pumps	B.Conc	100
40.00	Pumps	B.Conc	10
50.00	Controller	Stop	

Chromatogram

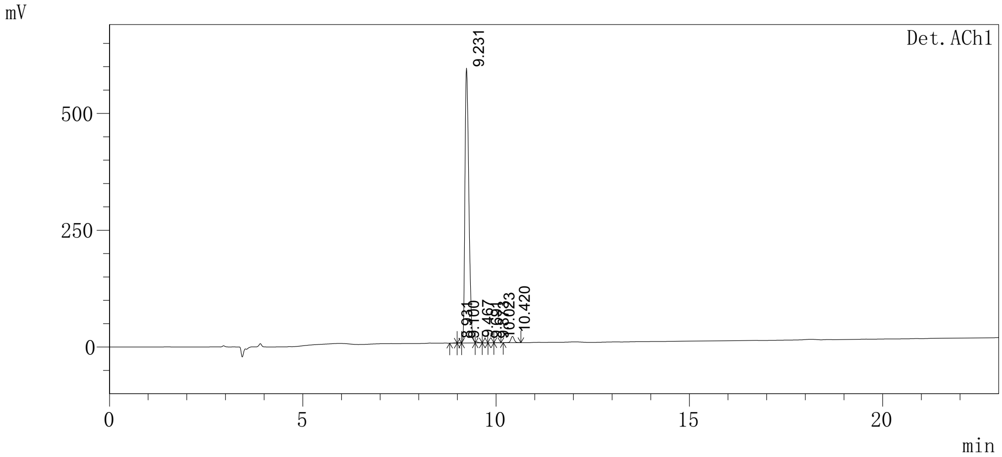

1 Det.A Ch1/220nm

PeakTable

DetectorACh1220nm

Peak#	Ret.Time	Area	Height	Area%	Height%
1	8.931	2743	421	0.064	0.069
2	9.100	6799	1257	0.160	0.207
3	9.231	4146879	588430	97.485	96.952
4	9.467	13416	2690	0.315	0.443
5	9.691	1772	316	0.042	0.052
6	9.873	759	148	0.018	0.024
7	10.023	1378	255	0.032	0.042
8	10.420	80102	13410	1.883	2.209
Total		4253848	606927	100.000	100.000

apVT

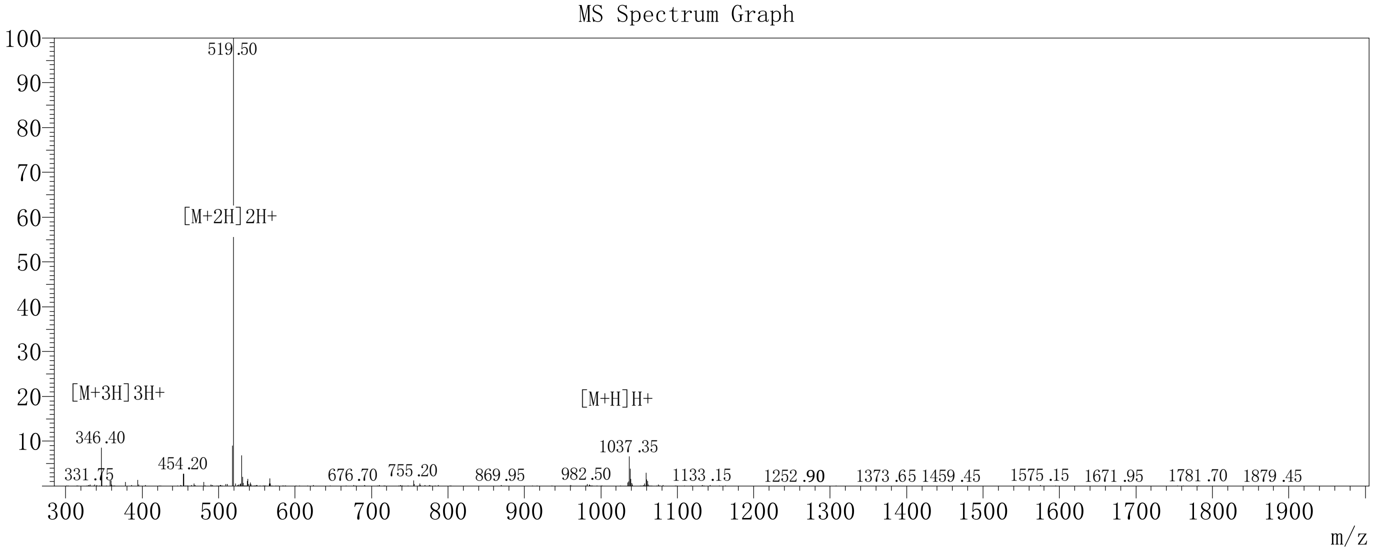

Sample Information

Dissolution method	:5%HAC+8%ACN+87%H2O	Interface	:ESI	Prerod Bias	:+1.5kv
Modified Date	:2021/11/15	Nebulizing Gas Flow	:1.50L/min	Detector	:-0.2kv
Injection Volume	:1ul	CDL Temp	:250C	T.Flow	:0.2ml/min
Heat Block Temp	:200	CDL Volt	:0v	B.conc	:50%H2O/50%MEOH

Order ID	:GP120518-1
Name	:apVT'-OH
Sequence	:C-F-I-R-N-C-P-K-G
Lot.No	:GP120518-1-1109
Theoretical	:1037.27
Observed	:1037.00

[A a]apVT

安徽省国平药业有限公司

CERTIFICATE OF ANALYSIS

Order ID	GP120556-4
Name	[Ala^2]apVT
Lot No.	GP120556-4-0104
Sequence	C-A-I-R-N-C-P-K-G*
Dissolution condition	100%H2O
Length	9AA
Modification	两个C形成二硫键，末端酰胺化
Molecular Weight (MW)	958.16
Storage	-20° C

Test Items	Specifications	Results
MW by MS	957.80	Conforms
Purityby HPLC	>95%	97.360%
Peptide Content	N/A	N/A
Moisture content	N/A	N/A
Acetic acid content	N/A	N/A
Appearance	White to off-white lyophilized powder	Conforms
Quantity	5mg	1.0mg*5

Certified by: LiuHui　　Date 02/16/2022

Quality Assurance Department

Note: this product is intended for research use only; not for diagnostic or human use.

Guoping Pharmaceutical Co., LTD

地址:合肥市经开区桃花工业园拓展区工投立恒工业广场A2 西F1,电话:0551-62841987 传真:0551-62841765 www.guopingyaoye.com

[A a]apVT

SampleInformation

OrderID:GP120556-4
Name: [Ala^{2}]apVT
Sequence:C*-A-I -R-N-C*-P-K-G-NH2
Lot.No:GP120556-4-0104
PumpA:0.1%TriĐluoroaceticin100% water
PumpB:0.1%TriĐluoroaceticin100% acetonitrile
TotalFlow:1ml/min
Wavelength:220nm
AnalyticalcolumntypeSHIMADZUInertsilODS-SP(4.6*250mm*5um)
Dissolutionmethod:100%H2O
Inj.Volume:16uL

TimeModule	Action	Value
0.01Pumps	B.Conc	10
20.00Pumps	B.Conc	50
23.00Pumps	B.Conc	100
38.00Pumps	B.Conc	100
40.00Pumps	B.Conc	10
50.00Controller	Stop	

Chromatogram

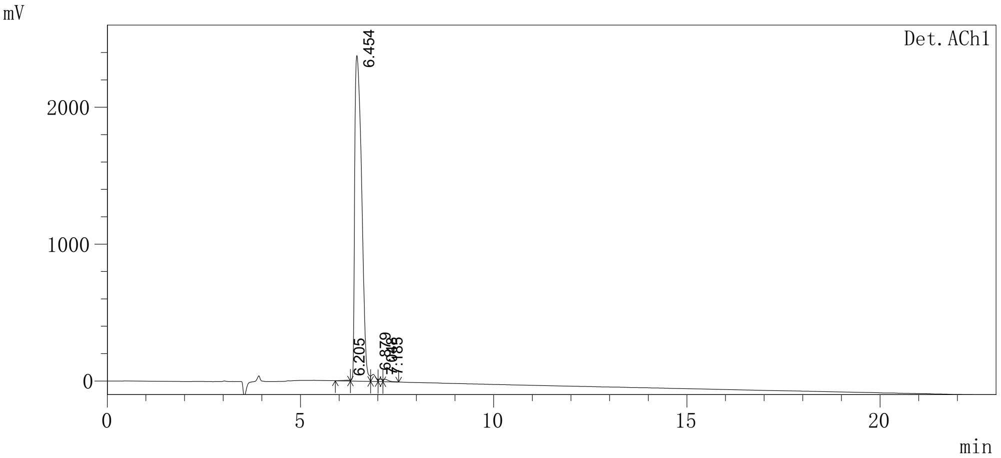

1 Det.A Ch1/220nm

PeakTable

DetectorACh1220nm

Peak#	Ret.Time	Area	Height	Area%	Height%
1	6.205	82667	5683	0.272	0.230
2	6.454	29562396	2379869	97.360	96.197
3	6.879	420640	51678	1.385	2.089
4	7.048	126553	18646	0.417	0.754
5	7.185	171627	18078	0.565	0.731
Total		30363884	2473954	100.000	100.000

[A a]apVT

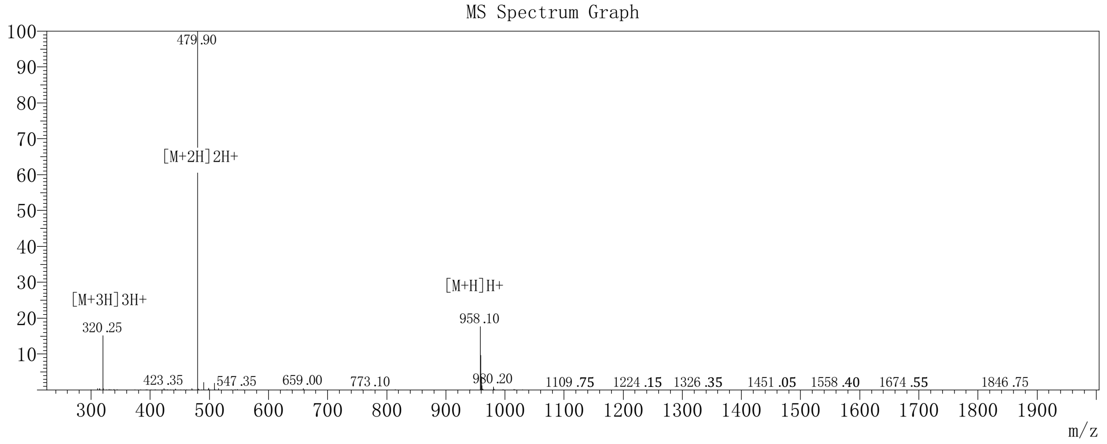

Sample Information

Dissolution method	:5%HAC+8%ACN+87%H2O	Interface	:ESI	Prerod Bias	:+1.5kv
Modified Date	:2022/01/21	Nebulizing Gas Flow	:1.50L/min	Detector	:-0.2kv
Injection Volume	:1ul	CDL Temp	:250C	T.Flow	:0.2ml/min
Heat Block Temp	:200	CDL Volt	:0v	B.conc	:50%H2O/50%MEOH

Order ID	:GP120556-4
Name	:[Ala^{2}]apVT
Sequence	:C*-A-I-R-N-C*-P-K-G-NH2
Lot.No	:GP120556-4-0104
Theoretical	:958.16
Observed	:957.80

[A a]apVT

安徽省国平药业有限公司

CERTIFICATE OF ANALYSIS

Order ID	GP120556-5
Name	[Ala^3]apVT
Lot No.	GP120556-5-0104
Sequence	C-F-A-R-N-C-P-K-G*
Dissolution condition	100%H2O
Length	9AA
Modification	两个C形成二硫键，末端酰胺化
Molecular Weight (MW)	992.17
Storage	-20° C

Test Items	Specifications	Results
MW by MS	991.90	Conforms
Purityby HPLC	>95%	99.364%
Peptide Content	N/A	N/A
Moisture content	N/A	N/A
Acetic acid content	N/A	N/A
Appearance	White to off-white lyophilized powder	Conforms
Quantity	5mg	1.0mg*5

Certified by: LiuHui Date 02/16/2022

Quality Assurance Department

Note: this product is intended for research use only; not for diagnostic or **hu**man use.

Guoping Pharmaceutical Co., LTD

地址:合肥市经开区桃花工业园拓展区工投立恒工业广场A2 西F1,电话:0551-62841987 传真:0551-62841765 www.guopingyaoye.com

[A a]apVT

SampleInformation

OrderID:GP120556-5
Name: [Ala[3]]apVT
Sequence:C*-F-A -R-N-C*-P-K-G-NH2
Lot.No:GP120556-5-0104
PumpA:0.1%TriĐluoroaceticin100% water
PumpB:0.1%TriĐluoroaceticin100% acetonitrile
TotalFlow:1ml/min
Wavelength:220nm
Analyticalcolumntype:SHIMADZUInertsilODS-SP(4.6*250mm*5um)
Dissolutionmethod:100%H2O
Inj.Volume:13uL

TimeModule	Action	Value
0.01Pumps	B.Conc	10
20.00Pumps	B.Conc	50
23.00Pumps	B.Conc	100
38.00Pumps	B.Conc	100
40.00Pumps	B.Conc	10
50.00Controller	Stop	

Chromatogram

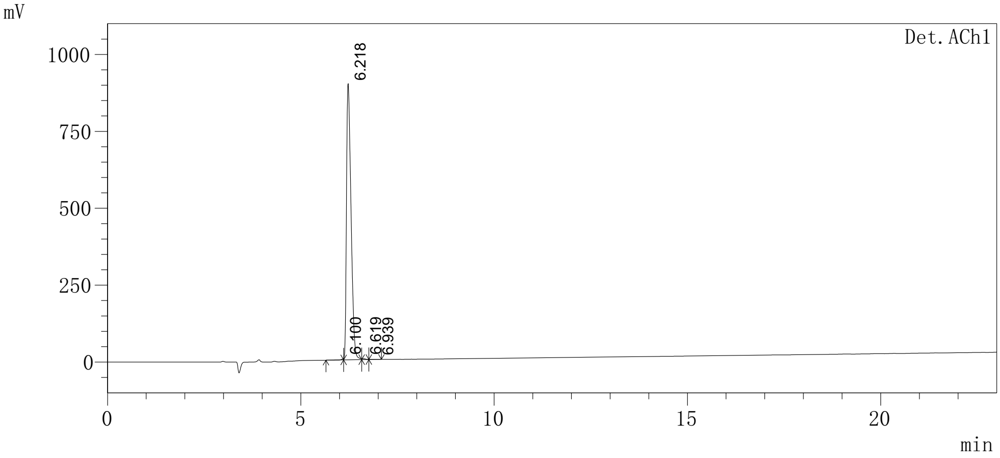

1 Det.A Ch1/220nm

PeakTable

DetectorACh1220nm

Peak#	Ret.Time	Area	Height	Area%	Height%
1	6.100	15853	4017	0.215	0.444
2	6.218	7335827	897721	99.364	99.137
3	6.619	20117	2986	0.272	0.330
4	6.939	10984	808	0.149	0.089
Total		7382780	905532	100.000	100.000

[A a]apVT

MS Spectrum Graph

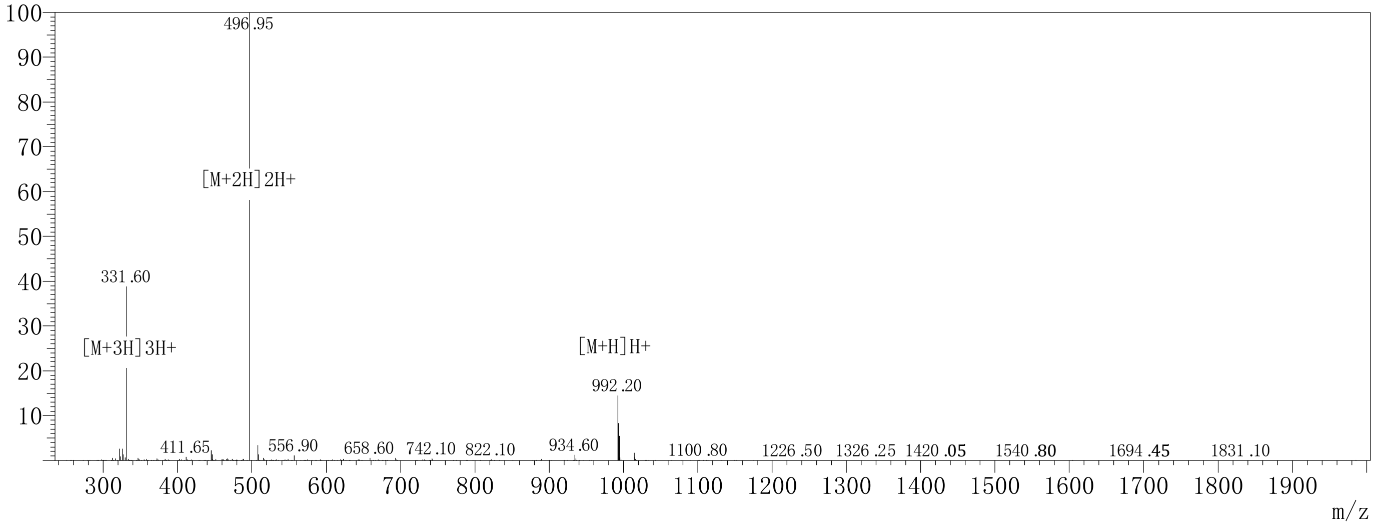

Sample Information

Dissolution method	:5%HAC+8%ACN+87%H2O	Interface	:ESI	Prerod Bias	:+1.5kv
Modified Date	:2022/01/21	Nebulizing Gas Flow	:1.50L/min	Detector	:-0.2kv
Injection Volume	:1ul	CDL Temp	:250C	T.Flow	:0.2ml/min
Heat Block Temp	:200	CDL Volt	:0v	B.conc	:50%H2O/50%MEOH

Order ID	:GP120556-5
Name	:[Ala^3]apVT
Sequence	:C*-F-A-R-N-C*-P-K-G-NH2
Lot.No	:GP120556-5-0104
Theoretical	:992.17
Observed	:991.90

[A a]apVT

安徽省国平药业有限公司

CERTIFICATE OF ANALYSIS

Order ID	GP120556-6
Name	[Ala^{4}]apVT
Lot No.	GP120556-6-0104
Sequence	C-F-I-A-N-C-P-K-G*
Dissolution condition	100%H2O
Length	9AA
Modification	两个C形成二硫键，末端酰胺化
Molecular Weight (MW)	949.15
Storage	-20° C

Test Items	Specifications	Results
MW by MS	948.90	Conforms
Purity by HPLC	>95%	95.425%
Peptide Content	N/A	N/A
Moisture content	N/A	N/A
Acetic acid content	N/A	N/A
Appearance	White to off-white lyophilized powder	Conforms
Quantity	5mg	1.0mg*5

Certified by: LiuHui Date 02/16/2022

Quality Assurance Department

Note: this product is intended for research use only; not for diagnostic or human use.

Guoping Pharmaceutical Co., LTD

地址:合肥市经开区桃花工业园拓展区工投立恒工业广场A2 西F1,电话:0551-62841987 传真:0551-62841765 www.guopingyaoye.com

[A a]apVT

SampleInformation

OrderID:GP120556-6
Name: [Ala[4]]apVT
Sequence:C*-F-I -A-N-C*-P-K-G-NH2
Lot.No:GP120556-6-0104
PumpA:0.1%TriĐluoroaceticin100% water
PumpB:0.1%TriĐluoroaceticin100% acetonitrile
TotalFlow:1ml/min
Wavelength:220nm
AnalyticalcolumntypeSHIMADZUInertsilODS-SP(4.6*250mm*5um)
Dissolutionmethod:100%H2O
Inj.Volume:14uL

TimeModule	Action	Value
0.01Pumps	B.Conc	10
20.00Pumps	B.Conc	50
23.00Pumps	B.Conc	100
38.00Pumps	B.Conc	100
40.00Pumps	B.Conc	10
50.00Controller	Stop	

Chromatogram

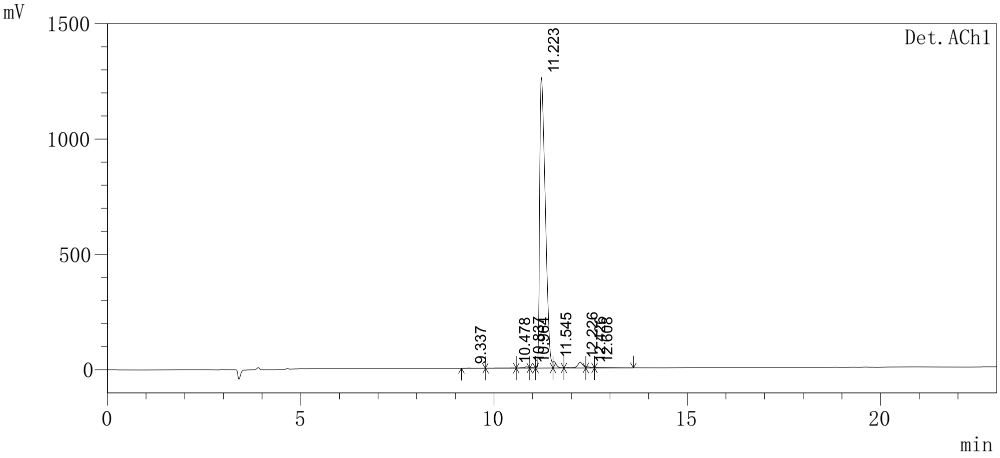

1 Det.A Ch1/220nm

PeakTable

DetectorACh1220nm

Peak#	Ret.Time	Area	Height	Area%	Height%
1	9.337	8737	933	0.066	0.070
2	10.478	13516	866	0.102	0.065
3	10.837	55729	5369	0.419	0.404
4	10.964	22959	3320	0.173	0.250
5	11.223	12684879	1259774	95.425	94.776
6	11.545	165495	27651	1.245	2.080
7	12.226	255829	24856	1.925	1.870
8	12.426	42579	4648	0.320	0.350
9	12.608	43349	1791	0.326	0.135
Total		13293071	1329208	100.000	100.000

[A a]apVT

MS Spectrum Graph

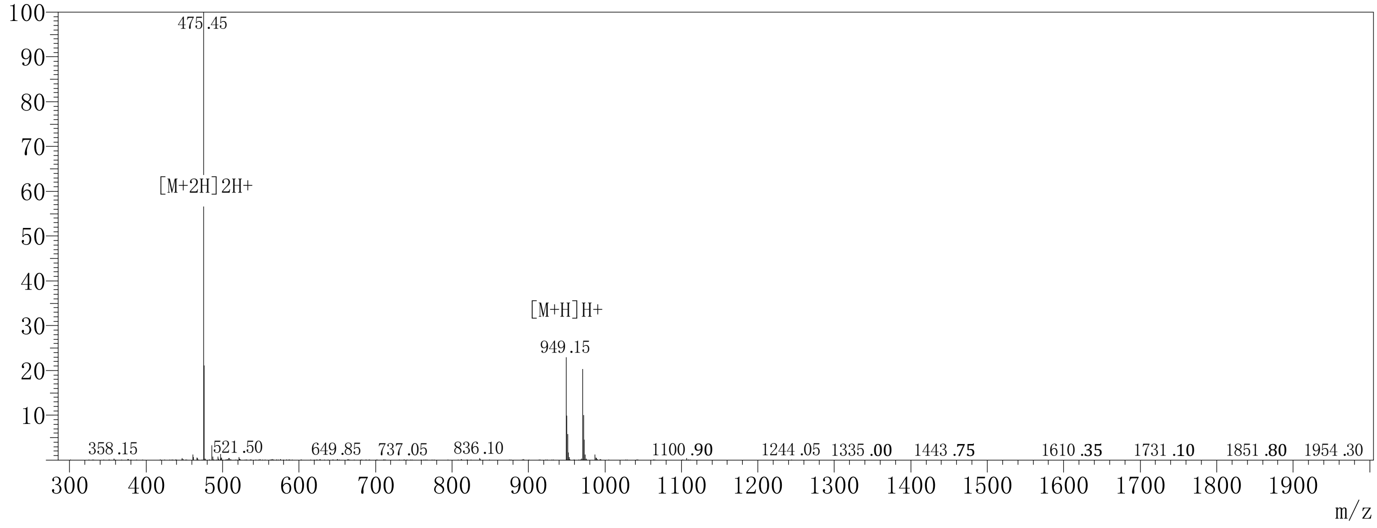

Sample Information

Dissolution method	:5%HAC+8%ACN+87%H2O	Interface	:ESI	Prerod Bias	:+1.5kv
Modified Date	:2022/01/20	Nebulizing Gas Flow	:1.50L/min	Detector	:-0.2kv
Injection Volume	:1ul	CDL Temp	:250C	T.Flow	:0.2ml/min
Heat Block Temp	:200	CDL Volt	:0v	B.conc	:50%H2O/50%MEOH

Order ID	:GP120556-6
Name	:[Ala^{4}]apVT
Sequence	:C*-F-I-A-N-C*-P-K-G-NH2
Lot.No	:GP120556-6-0104
Theoretical	:949.15
Observed	:948.90

[A a]apVT

安徽省国平药业有限公司

CERTIFICATE OF ANALYSIS

Order **ID**	GP120556-7
Name	[Ala[5]]apVT
Lot No.	GP120556-7-0104
Sequence	C-F-I-R-A-C-P-K-G*
**Diss**olution condition	100%H20
Length	9AA
Modification	两个C形成二硫键，末端酰胺化
MolecularWeight (M**W)**	991.23
Stor**age**	-20° C

Test **Items**	Specifications	**Results**
MW by MS	990.90	Conforms
Purityby HPLC	>95%	97.021%
Peptide Conte**n**t	N/A	N/A
Moisture conte**n**t	N/A	N/A
Acetic acid **content**	N/A	N/A
Appearance	White to off-white lyophilized powder	Conforms
Quan**tity**	5mg	1.0mg*5

Certified by: LiuHui Date 02/16/2022

Quality Assurance Department

Note: this product is intended for research use only; not for diagnostic or **hu**man use.

Guoping Pharmaceutical Co., LTD

地址:合肥市经开区桃花工业园拓展区工投立恒工业广场A2 西F1,电话:0551-62841987 传真:0551-62841765 www.guopingyaoye.com

[A a]apVT

SampleInformation

OrderID:GP120556-7
Name: [Ala^5]apVT
Sequence:C*-F-I -R-A-C*-P-K-G-NH2
Lot.No:GP120556-7-0104
PumpA:0.1%TriĐluoroaceticin100% water
PumpB:0.1%TriĐluoroaceticin100% acetonitrile
TotalFlow:1ml/min
Wavelength:220nm
AnalyticalcolumntypeSHIMADZUInertsilODS-SP(4.6*250mm*5um)
Dissolutionmethod:100%H2O
Inj.Volume:8uL

TimeModule	Action	Value
0.01Pumps	B.Conc	10
20.00Pumps	B.Conc	50
23.00Pumps	B.Conc	100
38.00Pumps	B.Conc	100
40.00Pumps	B.Conc	10
50.00Controller	Stop	

Chromatogram

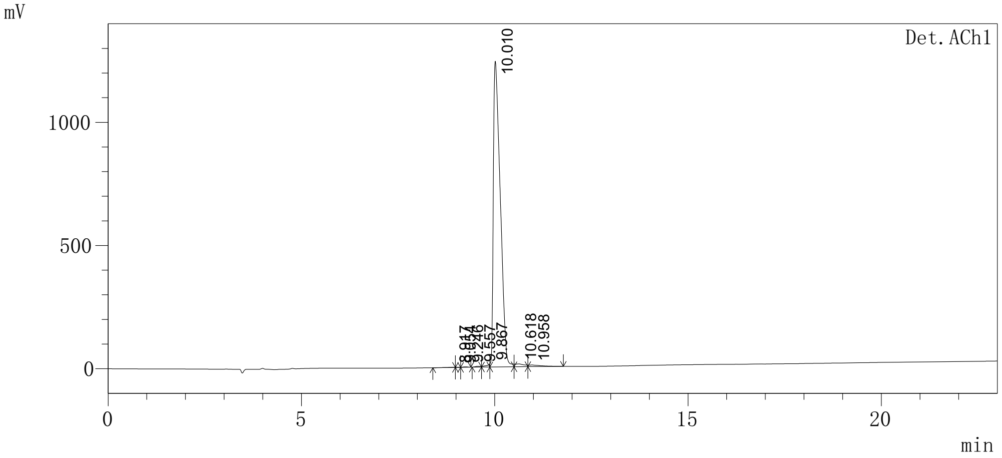

1 Det.A Ch1/220nm

PeakTable

DetectorACh1220nm

Peak#	Ret.Time	Area	Height	Area%	Height%
1	8.917	9263	784	0.058	0.061
2	9.054	3142	482	0.020	0.038
3	9.246	14703	1729	0.092	0.135
4	9.557	29675	3112	0.186	0.244
5	9.867	78132	11614	0.491	0.910
6	10.010	15445007	1240715	97.021	97.200
7	10.618	193753	11487	1.217	0.900
8	10.958	145629	6527	0.915	0.511
Total		15919305	1276450	100.000	100.000

[A a]apVT

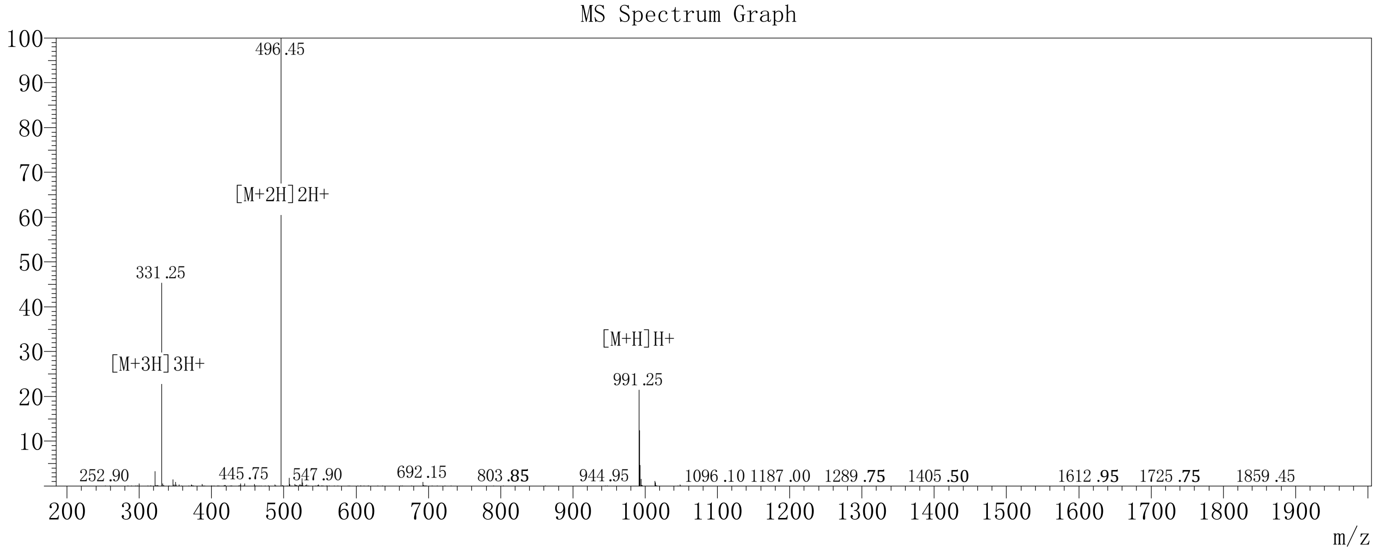

Sample Information

Dissolution method	:5%HAC+8%ACN+87%H2O	Interface	:ESI	Prerod Bias	:+1.5kv
Modified Date	:2022/01/22	Nebulizing Gas Flow	:1.50L/min	Detector	:-0.2kv
Injection Volume	:1ul	CDL Temp	:250C	T.Flow	:0.2ml/min
Heat Block Temp	:200	CDL Volt	:0v	B.conc	:50%H2O/50%MEOH

Order ID	:GP120556-7
Name	:[Ala^{5}]apVT
Sequence	:C*-F-I-R-A-C*-P-K-G-NH2
Lot.No	:GP120556-7-0104
Theoretical	:991.23
Observed	:990.90

[A a]apVT

安徽省国平药业有限公司

CERTIFICATE OF ANALYSIS

Order ID	GP120556-8
Name	[Ala[7]]apVT
Lot No.	GP120556-8-0104
Sequence	C-F-I-R-N-C-A-K-G*
**Diss**olution condition	100%H20
Length	9AA
Modification	两个C形成二硫键，末端酰胺化
Molecular Weight (M**W)**	1008.22
Stor**age**	-20° C

Tes**t Items**	Specifications	**Results**
MW by MS	1007.90	Conforms
Purityby HPLC	>95%	95.948%
Peptide Conte**n**t	N/A	N/A
Moisture conte**n**t	N/A	N/A
Acetic acid **content**	N/A	N/A
Appearance	White to off-white lyophilized powder	Conforms
Quan**tity**	5mg	1.0mg*5

Certified by: LiuHui   Date 02/16/2022

Quality Assurance Department

Note: this product is intended for research use only; not for diagnostic or **hu**man use.

Guoping Pharmaceutical Co., LTD

地址:合肥市经开区桃花工业园拓展区工投立恒工业广场A2 西F1,电话:0551-62841987 传真:0551-62841765 www.guopingyaoye.com

[A a]apVT

SampleInformation

OrderID:GP120556-8
Name: [Ala7]apVT
Sequence:C*-F-I-R-N-C*-A-K-G-NH2
Lot.No:GP120556-8-0104
PumpA:0.1%TriĐluoroaceticin100% water
PumpB:0.1%TriĐluoroaceticin100% acetonitrile
TotalFlow:1ml/min
Wavelength:220nm
AnalyticalcolumntypeSHIMADZUInertsilODS-SP(4.6*250mm*5um)
Dissolutionmethod:100%H2O
Inj.Volume:14uL

TimeModule	Action	Value
0.01Pumps	B.Conc	10
20.00Pumps	B.Conc	50
23.00Pumps	B.Conc	100
38.00Pumps	B.Conc	100
40.00Pumps	B.Conc	10
50.00Controller	Stop	

Chromatogram

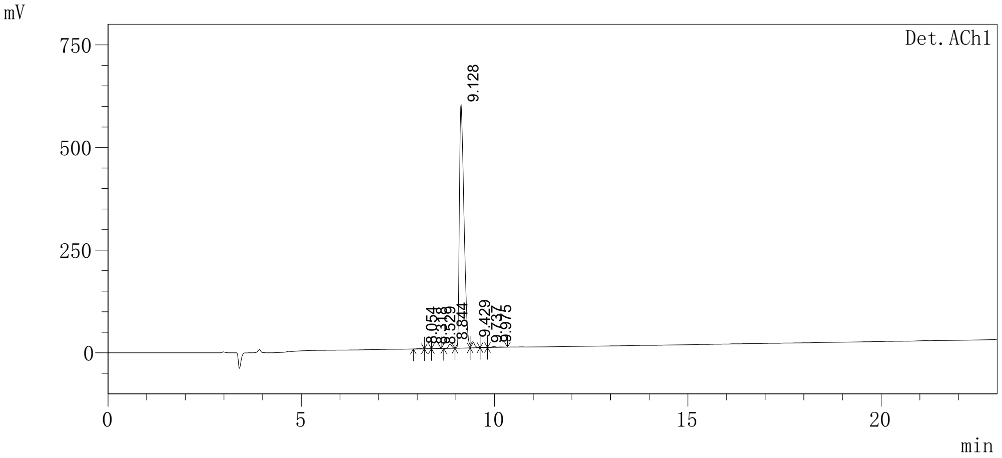

1 Det.A Ch1/220nm

PeakTable

DetectorACh1220nm

Peak#	Ret.Time	Area	Height	Area%	Height%
1	8.054	8312	1355	0.164	0.217
2	8.318	798	105	0.016	0.017
3	8.529	1583	311	0.031	0.050
4	8.844	60934	10372	1.200	1.664
5	9.128	4870388	593115	95.948	95.161
6	9.429	100077	15014	1.972	2.409
7	9.737	8819	936	0.174	0.150
8	9.975	25137	2066	0.495	0.331
Total		5076048	623275	100.000	100.000

[A a]apVT

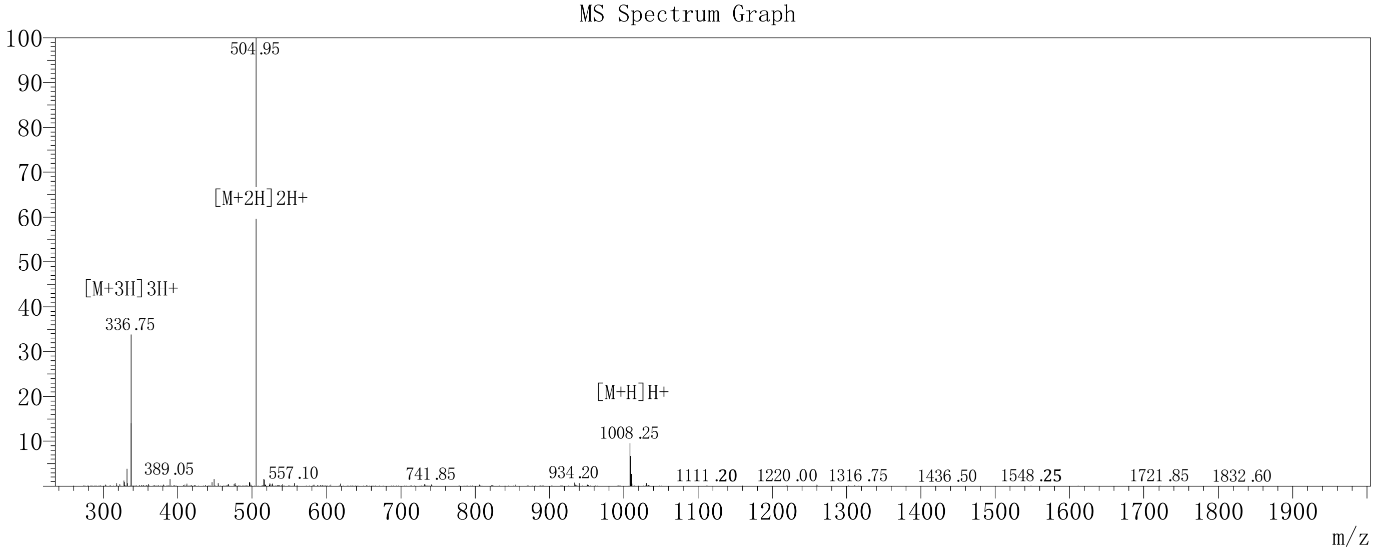

Sample Information

Dissolution method	:5%HAC+8%ACN+87%H20	Interface	:ESI	Prerod Bias	:+1.5kv
Modified Date	:2022/01/21	Nebulizing Gas Flow	:1.50L/min	Detector	:-0.2kv
Injection Volume	:1ul	CDL Temp	:250C	T.Flow	:0.2ml/min
Heat Block Temp	:200	CDL Volt	:0v	B.conc	:50%H20/50%MEOH

Order ID :GP120556-8
Name :[Ala^{7}]apVT
Sequence :C*-F-I-R-N-C*-A-K-G-NH2
Lot.No :GP120556-8-0104
Theoretical :1008.22
Observed :1007.90

[A a]apVT

安徽省国平药业有限公司

CERTIFICATE OF ANALYSIS

Order ID	GP120556-9
Name	[Ala[8]]apVT
Lot No.	GP120556-9-0104
Sequence	C-F-I-R-N-C-P-A-G*
Dissolution condition	100%H2O
Length	9AA
Modification	两个C形成二硫键，末端酰胺化
Molecular Weight (MW)	977.16
Storage	-20° C

Test Items	Specifications	Results
MW by MS	976.60	Conforms
Purityby HPLC	>95%	95.507%
Peptide Content	N/A	N/A
Moisture content	N/A	N/A
Acetic acid content	N/A	N/A
Appearance	White to off-white lyophilized powder	Conforms
Quantity	5mg	1.0mg*5

Certified by: LiuHui Date 02/16/2022

Quality Assurance Department

Note: this product is intended for research use only; not for diagnostic or human use.

Guoping Pharmaceutical Co., LTD

地址:合肥市经开区桃花工业园拓展区工投立恒工业广场A2 西F1,电话:0551-62841987 传真:0551-62841765 www.guopingyaoye.com

[A a]apVT

SampleInformation

OrderID:GP120556-9
Name: [Ala^{8}]apVT
Sequence:C*-F-I -R-N-C*-P-A-G-NH2
Lot.No:GP120556-9-0104
PumpA:0.1%TriĐluoroaceticin100% water
PumpB:0.1%TriĐluoroaceticin100% acetonitrile
TotalFlow:1ml/min
Wavelength:220nm
Analyticalcolumntype:SHIMADZUInertsilODS-SP(4.6*250mm*5um)
Dissolutionmethod:100%H2O
Inj.Volume:16uL

TimeModule	Action	Value
0.01Pumps	B.Conc	10
20.00Pumps	B.Conc	50
23.00Pumps	B.Conc	100
38.00Pumps	B.Conc	100
40.00Pumps	B.Conc	10
50.00Controller	Stop	

Chromatogram

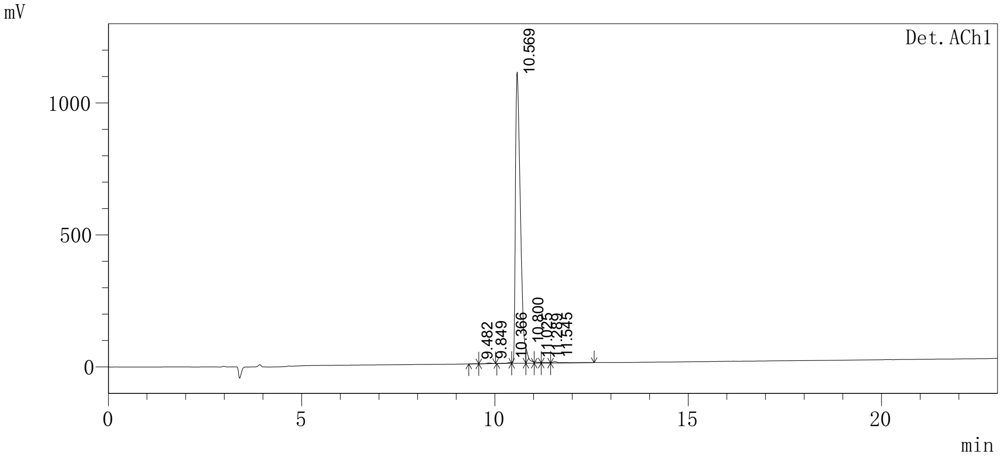

1 Det.A Ch1/220nm

PeakTable

DetectorACh1220nm

Peak#	Ret.Time	Area	Height	Area%	Height%
1	9.482	8393	1363	0.083	0.115
2	9.849	21384	2775	0.210	0.235
3	10.366	26695	3184	0.263	0.269
4	10.569	9706554	1103223	95.507	93.321
5	10.800	239161	59392	2.353	5.024
6	11.025	35689	4405	0.351	0.373
7	11.289	30688	2588	0.302	0.219
8	11.545	94601	5248	0.931	0.444
Total		10163164	1182179	100.000	100.000

[A a]apVT

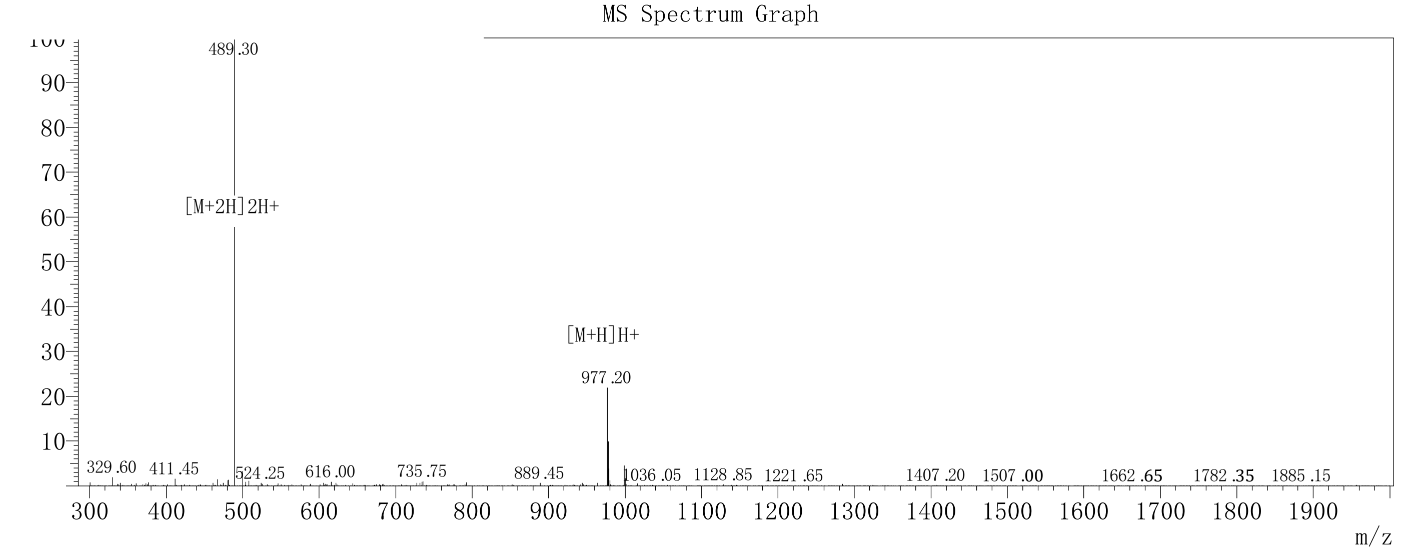

Sample Information

Dissolution method	:5%HAC+8%ACN+87%H2O	Interface	:ESI	Prerod Bias	:+1.5kv
Modified Date	:2022/01/21	Nebulizing Gas Flow	:1.50L/min	Detector	:-0.2kv
Injection Volume	:1ul	CDL Temp	:250C	T.Flow	:0.2ml/min
Heat Block Temp	:200	CDL Volt	:0v	B.conc	:50%H2O/50%MEOH

Order ID :GP120556-9
Name :[Ala^{8}]apVT
Sequence :C*-F-I-R-N-C*-P-A-G-NH2
Lot.No :GP120556-9-0104
Theoretical :977.16
Observed :976.60

[A a]apVT

安徽省国平药业有限公司

CERTIFICATE OF ANALYSIS

Order ID	GP120557
Name	[Ala9]apVT
Lot No.	GP120557-0106
Sequence	C-F-I-R-N-C-P-K-A*
Dissolution condition	100%H2O
Length	9AA
Modification	两个C形成二硫键，末端酰胺化
Molecular Weight (MW)	1048.30
Storage	-20° C

Test Items	Specifications	Results
MW by MS	1048.10	Conforms
Purityby HPLC	>95%	96.276%
Peptide Content	N/A	N/A
Moisture content	N/A	N/A
Acetic acid content	N/A	N/A
Appearance	White to off-white lyophilized powder	Conforms
Quantity	5mg	1.0mg*5

Certified by: LiuHui Date 02/16/2022

Quality Assurance Department

Note: this product is intended for research use only; not for diagnostic or **hu**man use.

Guoping Pharmaceutical Co., LTD

地址:合肥市经开区桃花工业园拓展区工投立恒工业广场A2 西F1,电话:0551-62841987 传真:0551-62841765 www.guopingyaoye.com

[A a]apVT

SampleInformation

OrderID:GP120557
Name: [Ala^9]apVT
Sequence:C*-F-I -R-N-C*-P-K-A-NH2
Lot.No:GP120557-0106
PumpA:0.1%TriĐluoroaceticin100% water
PumpB:0.1%TriĐluoroaceticin100% acetonitrile
TotalFlow:1ml/min
Wavelength:220nm
AnalyticalcolumntypeSHIMADZUInertsilODS-SP(4.6*250mm*5um)
Dissolutionmethod:100%H2O
Inj.Volume:14uL

TimeModule	Action	Value
0.01Pumps	B.Conc	15
20.00Pumps	B.Conc	55
23.00Pumps	B.Conc	100
38.00Pumps	B.Conc	100
40.00Pumps	B.Conc	15
50.00Controller	Stop	

Chromatogram

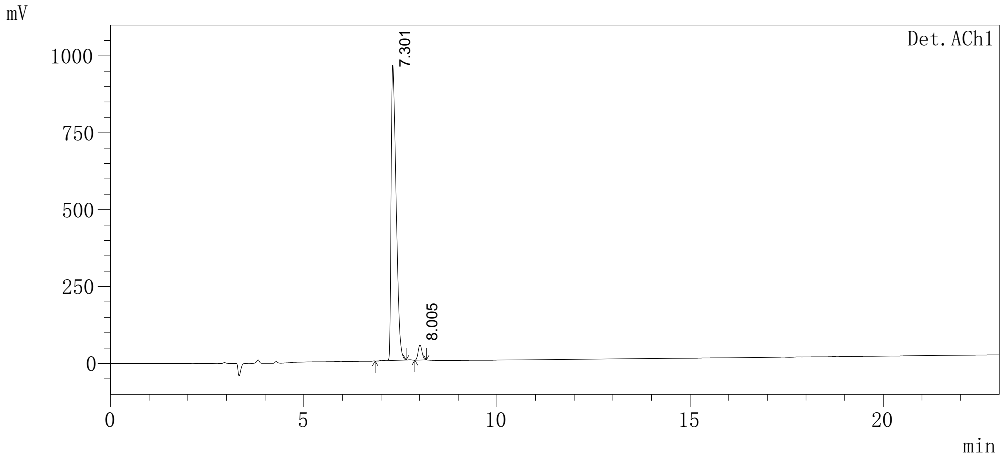

1 Det.A Ch1/220nm

PeakTable

DetectorACh1220nm

Peak#	Ret.Time	Area	Height	Area%	Height%
1	7.301	8643817	960855	96.276	95.129
2	8.005	334383	49204	3.724	4.871
Total		8978200	1010058	100.000	100.000

[A a]apVT

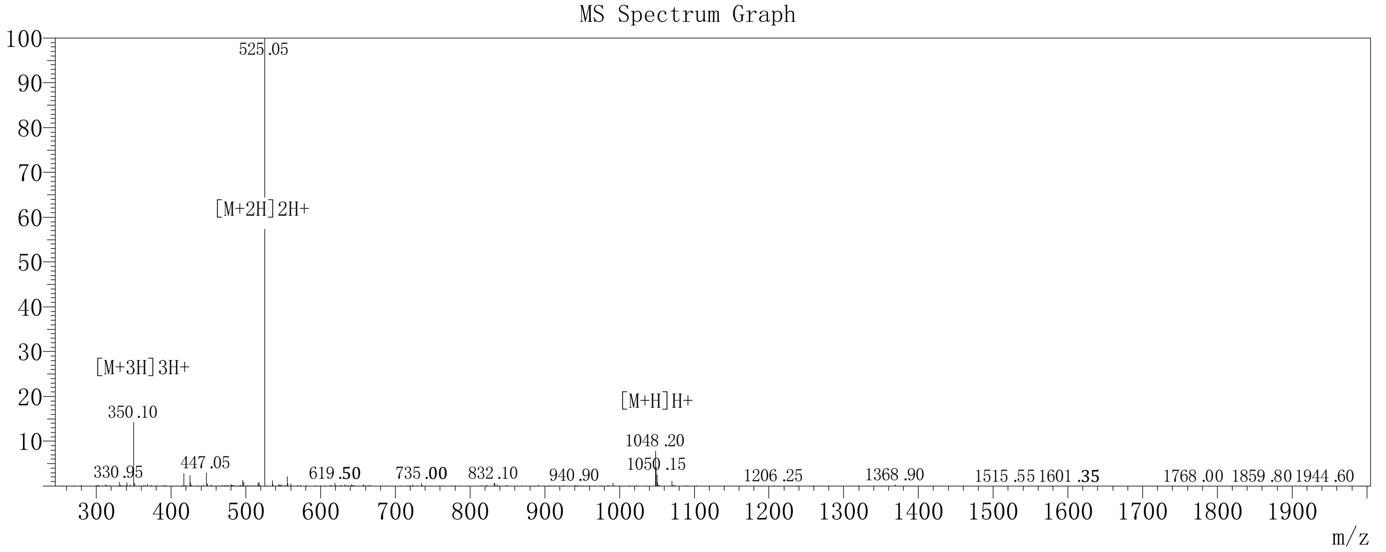

Sample Information

Dissolution method	:5%HAC+8%ACN+87%H20	Interface	:ESI	Prerod Bias	:+1.5kv
Modified Date	:2022/01/20	Nebulizing Gas Flow	:1.50L/min	Detector	:-0.2kv
Injection Volume	:1ul	CDL Temp	:250C	T.Flow	:0.2ml/min
Heat Block Temp	:200	CDL Volt	:0v	B.conc	:50%H2O/50%MEOH

Order ID	:GP120557
Name	:[Ala^9]apVT
Sequence	:C*-F-I-R-N-C*-P-K-A-NH2
Lot.No	:GP120557-0106
Theoretical	:1048.30
Observed	:1048.10
