# Supplementary material for: Characterization of an Aplysia vasotocin signaling system and actions of posttranslational modifications and individual residues of the ligand on receptor activity
Source: Front Pharmacol. 2023 Mar 20;14:1132066. doi: 10.3389/fphar.2023.1132066 (PMC10067623; doi:10.3389/fphar.2023.1132066)
Supplement: Supplementary file 6 [file DataSheet2.DOCX]

# *Supplementary Materials*

**Characterization of an *Aplysia* vasotocin signaling system and the functions of posttranslational modifications and individual residues of the ligand on receptor activity**

Ju-Ping Xu^1+^, Xue-Ying Ding^1+^, Shi-Qi Guo^1^, Hui-Ying Wang^1^, Wei-Jia Liu^1^, Hui-Min Jiang^1^, Ya-Dong Li^1^, Ping Fu^1^, Ping Chen^1^, Yu-Shuo Mei^1^, Guo Zhang^1*^, Hai-Bo Zhou^2*^, Jian Jing^1,3,4*^

* Corresponding authors;

E-mail: Jian Jing^1, 3, 4*^, [jingj01@live.com](mailto:jingj01@live.com); Hai-Bo Zhou^2*^, [haibozhou@nju.edu.cn](mailto:haibozhou@nju.edu.cn); Guo Zhang^1*^, [zhangguonanda@163.com](mailto:zhangguonanda@163.com)

There is a total of eleven supporting figures (**Supplementary Figures S1-S10**) and five supporting tables (**Supplementary Tables S1-S5**). **Supplementary Figures S2-S10** are included in this document, whereas **Supplementary Figure S1** is included as a separate PDF document with its legend provided below. There are five supporting tables, four of them are included as separate Excel files (**Supplementary Table S1-S3.**xlsx and **Supplementary Table S5**), whereas the legends for these tables are provided below; and one of them is included in this file as **Supplementary Table S4**.

**Supplementary Figure S1.** Peptide synthesis information from commercial companies.

**Supplementary Table S1.** List of primers used for PCR of the putative *Aplysia* Vasotocin precursor and receptors.

**Supplementary Table S2.** List of peptides from different species used to generate **Figure 1A**.

**Supplementary Table S3.** NCBI search, Pfam, and Clustal Omega table information of the protein sequences for **Figure 4C**.

**Supplementary Table S4.** The results from pblast with Class-A GPCR 3 for sequences from *Mus musculus*, *Danio,* or *C. elegans* in NCBI.

**Supplementary Table S5.** List of vasopressin/oxytocin-like peptide receptors from different species used to generate **Figure 6** (NCBI information and Clustal table).

1. **Supplementary Figures**

**Supplementary Figure S2**


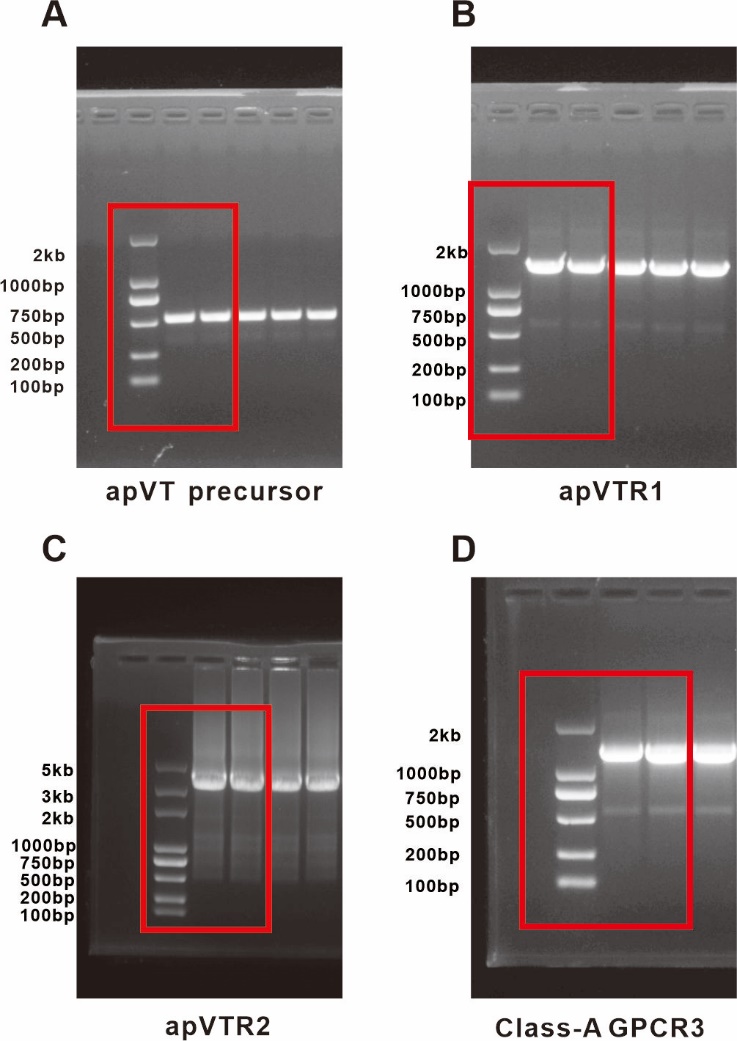


**Supplementary Figure S2.** The original agarose gels show where the gels in **Figure 3-4** come from. **(A)** The PCR products of vasotocin precursor. The gel marked in the red square is shown in **Figure 3A. (B)** The PCR products of vasotocin receptor 1. The gel marked in the red square is shown in **the left panel of Figure 4A**. **(C)** The PCR products of vasotocin receptor 2. The gel marked in the red square is shown in **the middle panel of Figure 4A**. **(D)** The PCR products of Class-A GPCR 3. The gel marked in the red square is shown in **the right panel of Figure 4A**.

**Supplementary Figure S3**


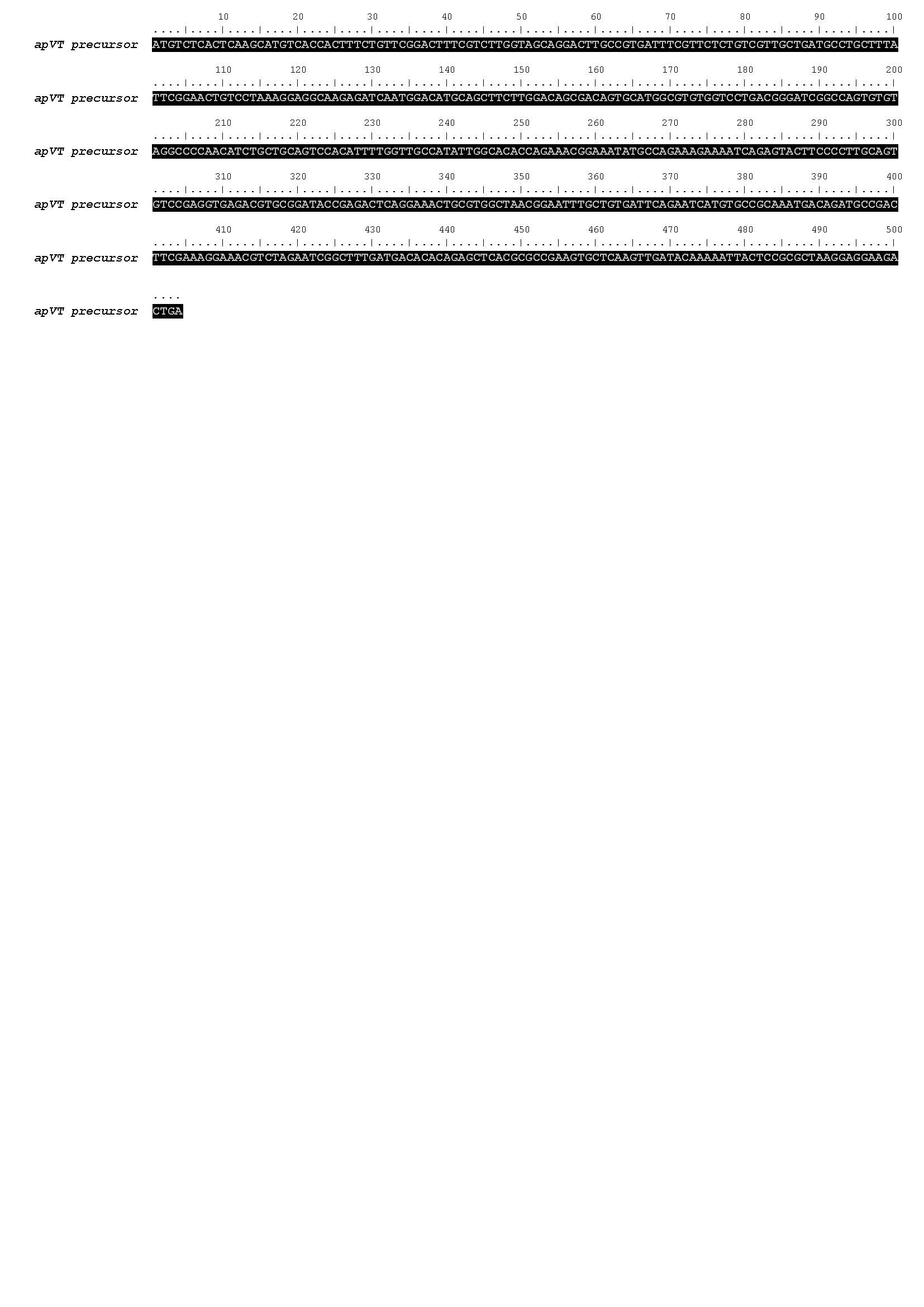


**Supplementary Figure S3.** The cloned cDNA sequence for the apVT precursor. The sequence is identical to XM_013084328.1.

**Supplementary Figure S4**


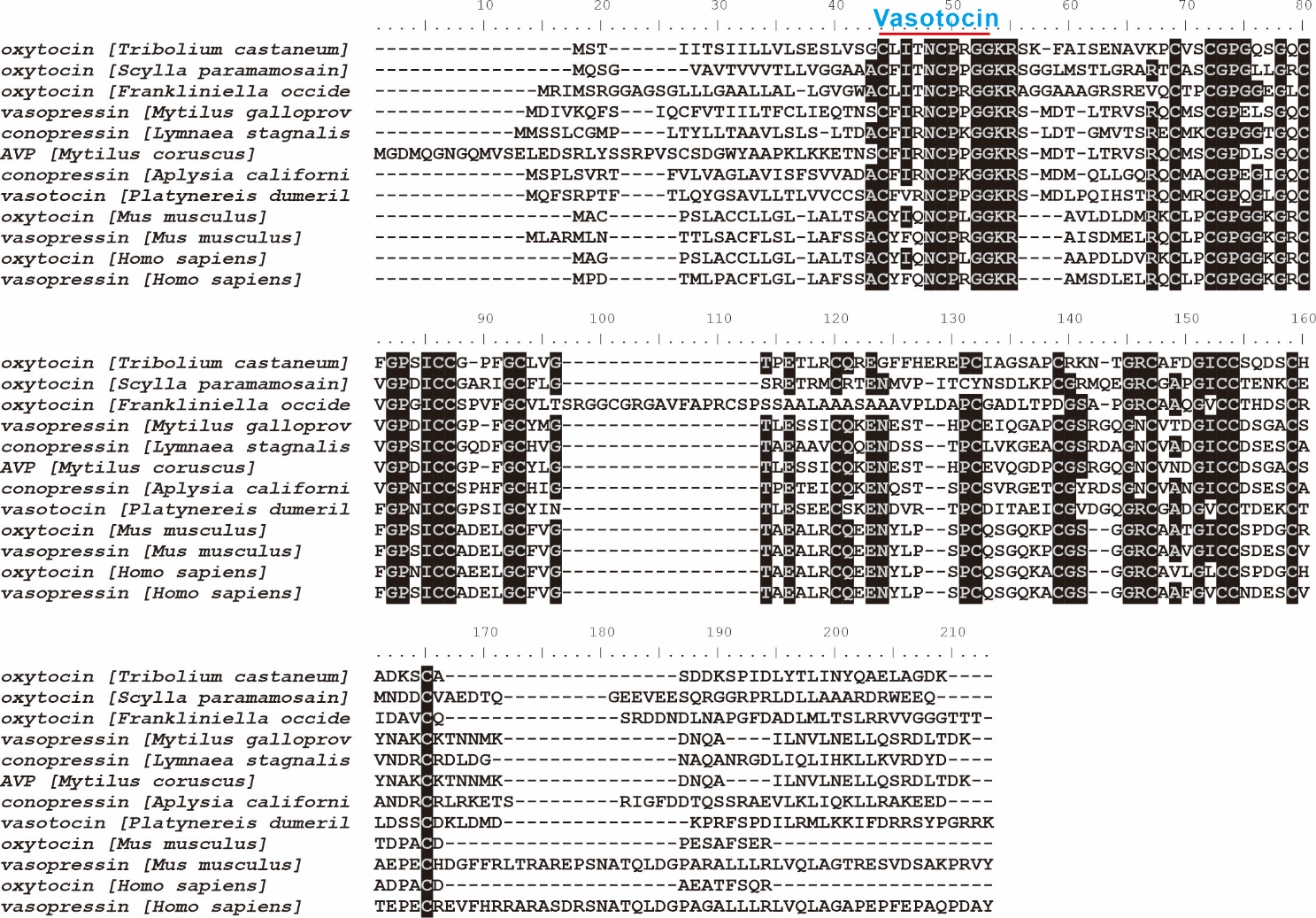


**Supplementary Figure S4.** The alignment between apVT precursor and related precursors in other species. List of species (the similarity to apVT is shown in parenthesis), Arthropods: *Tribolium_castaneum* (35.66%), *Scylla_paramamosain* (37.75%), *Frankliniella_occidentalis* (33.33%); Molluscs: *Mytilus_galloprovincialis* (47.65%), *Lymnaea_stagnalis* (56.49%), *Mytilus_coruscus* (48.34%); Annelids: *Platynereis_dumerilii* (42.86%); Mammals: *Mus_musculus* (oxytocin (39.2%), vasopressin (38.06%)), *Homo_sapiens* (oxytocin (39.2%), vasopressin (40.67%)).

**Supplementary Figure S5**

**A**


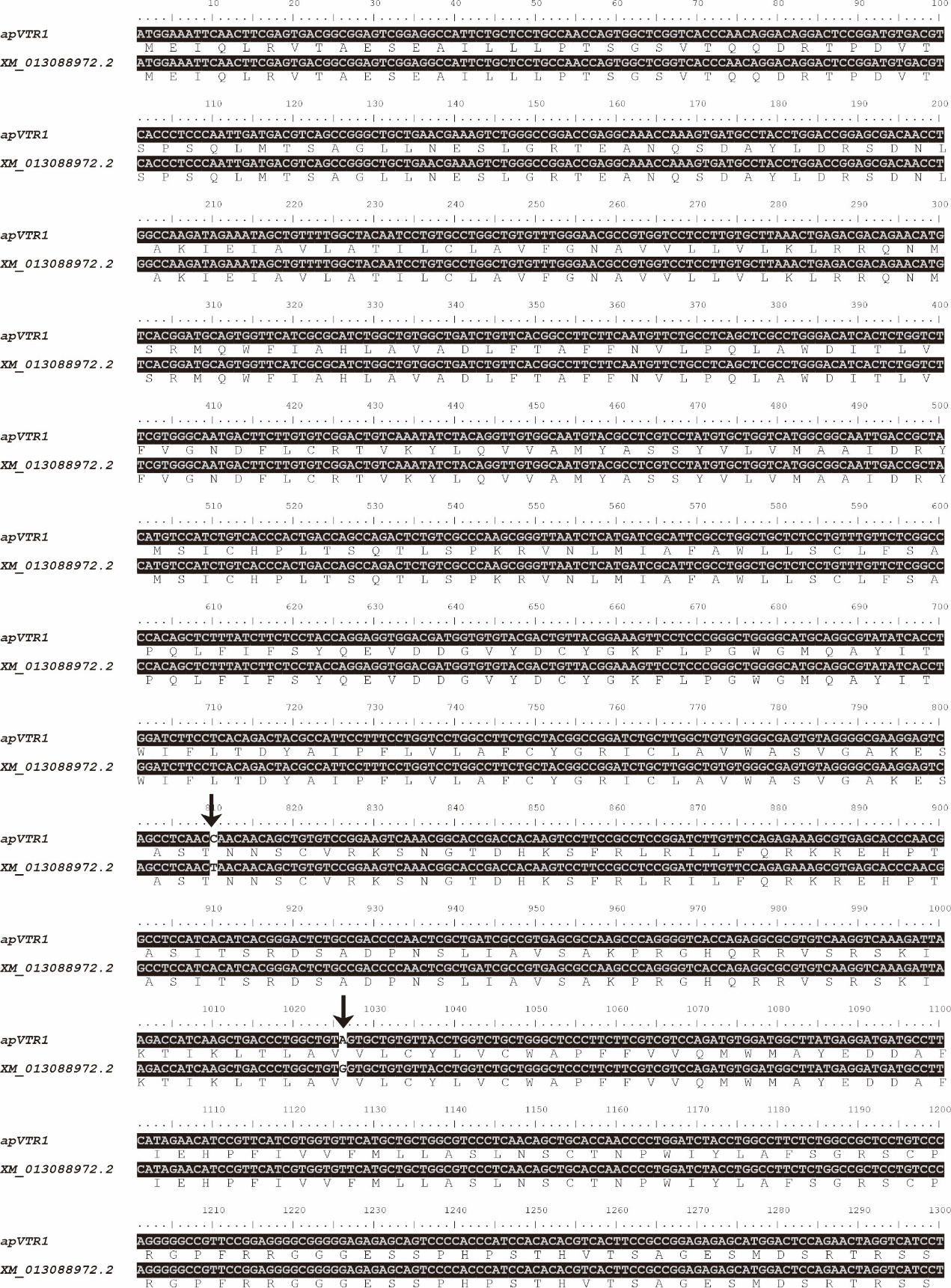


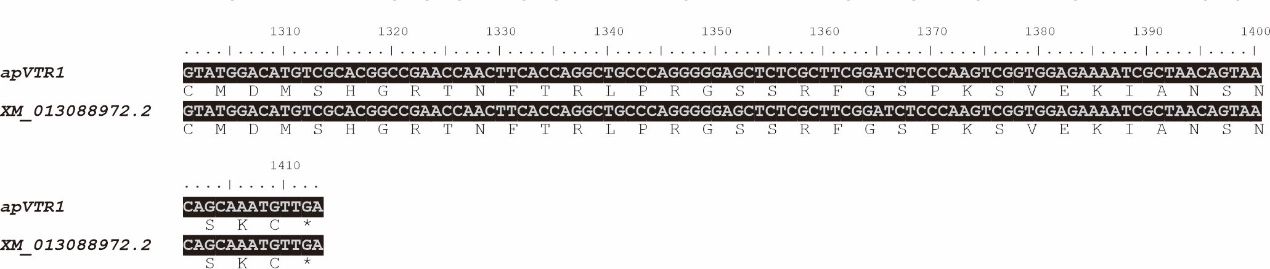


**B**
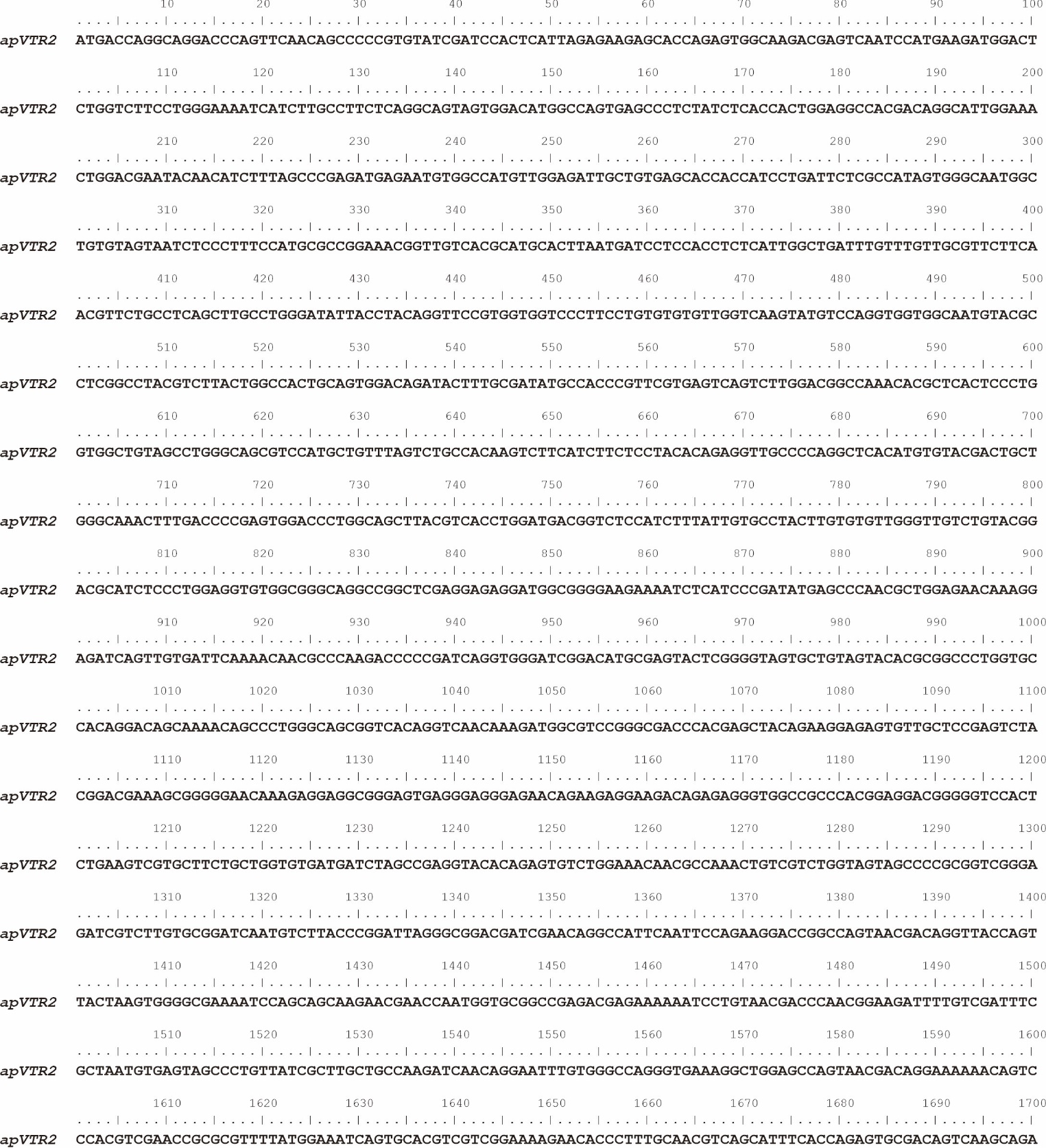


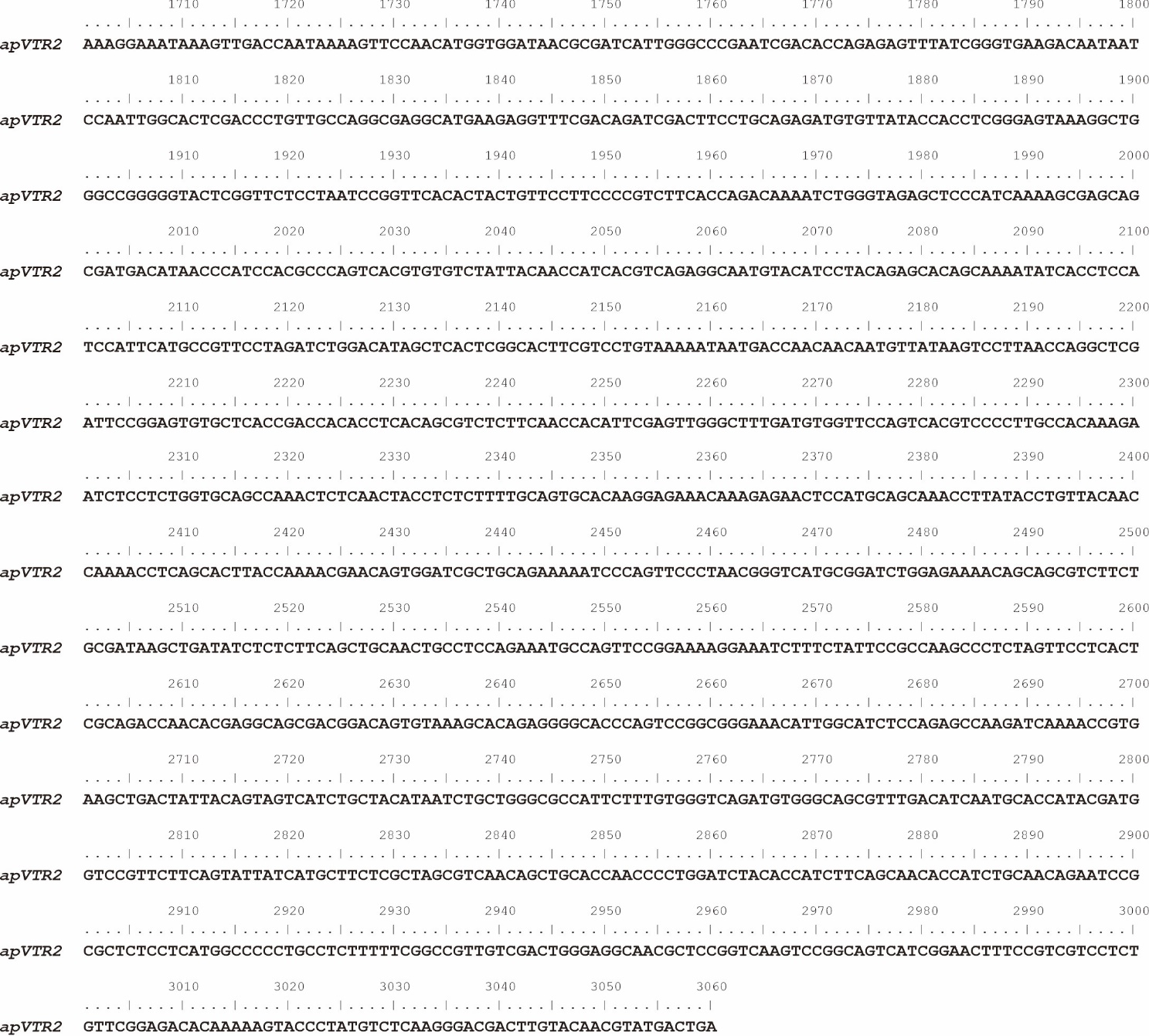


**C**


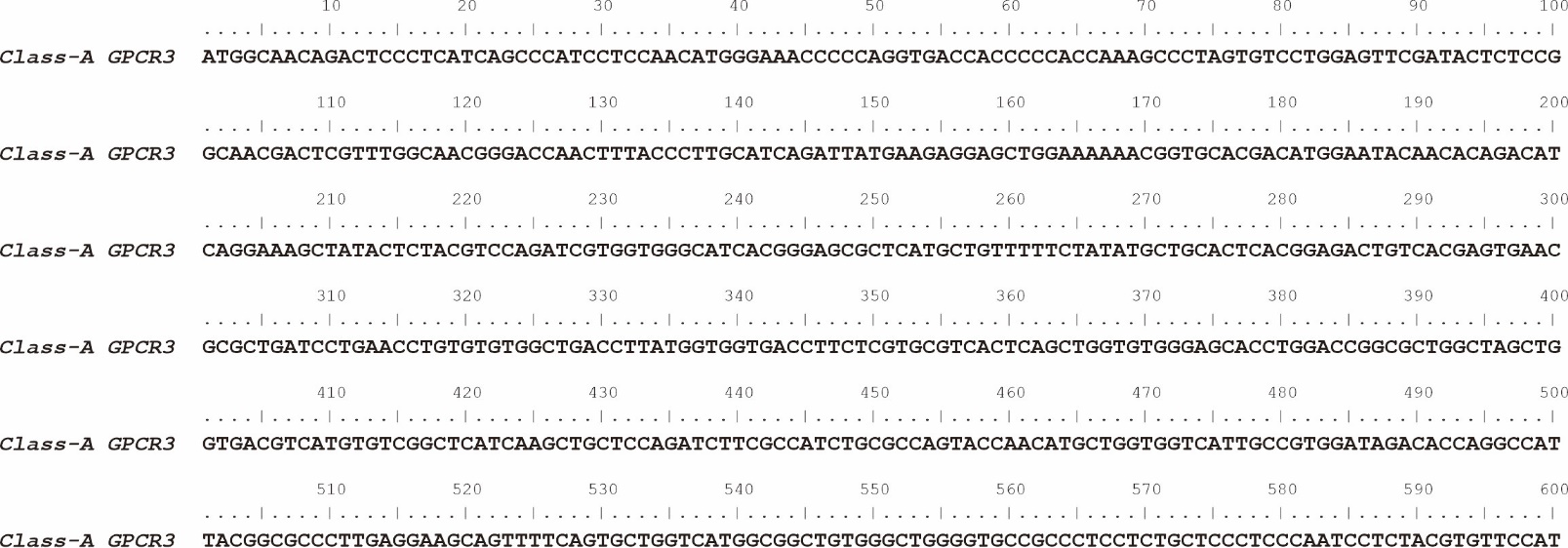


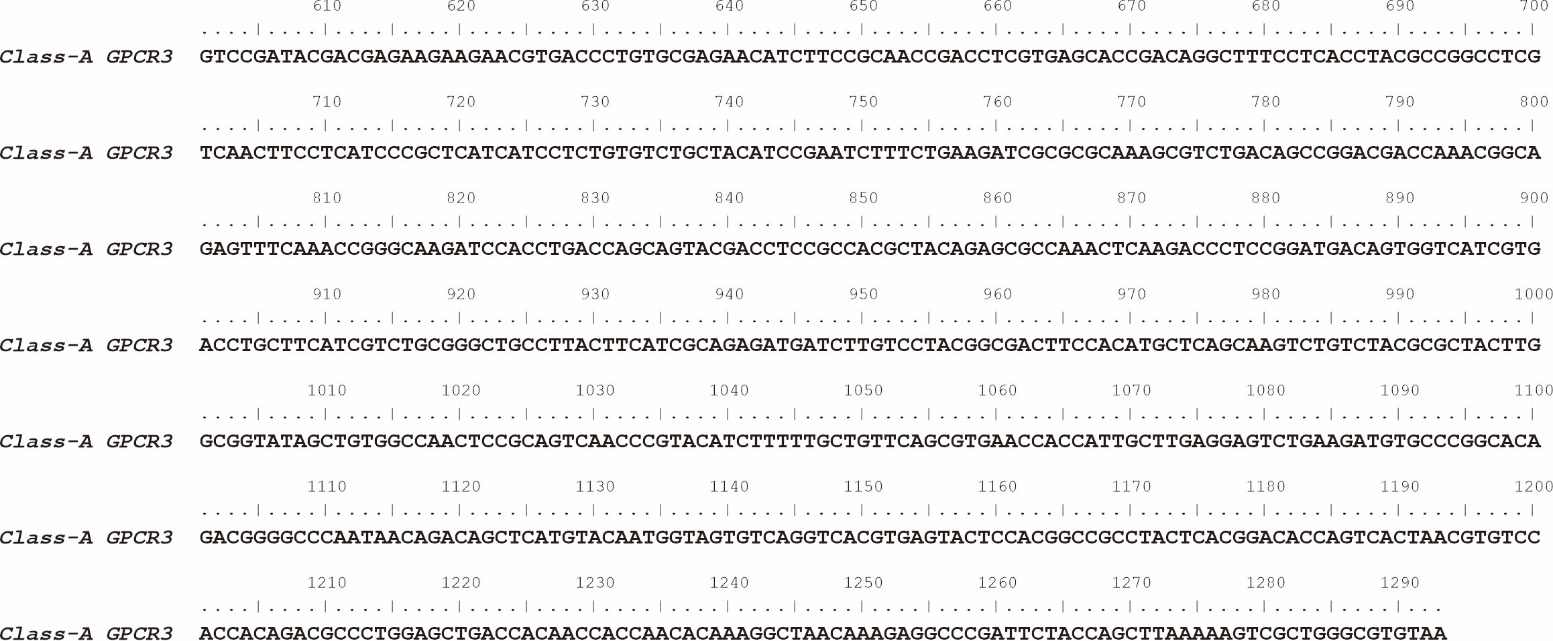


**Supplementary Figure S5.** The three putative receptor sequences we have cloned. **(A)** The alignment of our cloned sequence (apVTR1) and the corresponding NCBI sequence (XM_013088972.2) using BioEdit (ClustalW Multiple alignments - Graphic View). There are two single nucleotide polymorphisms (SNPs, arrows) but these SNPs don’t change the amino acid sequence. **(B)** The apVTR2 sequence we have cloned. **(C)** The Class-A GPCR3 sequence we have cloned.

**Supplementary Figure S6**


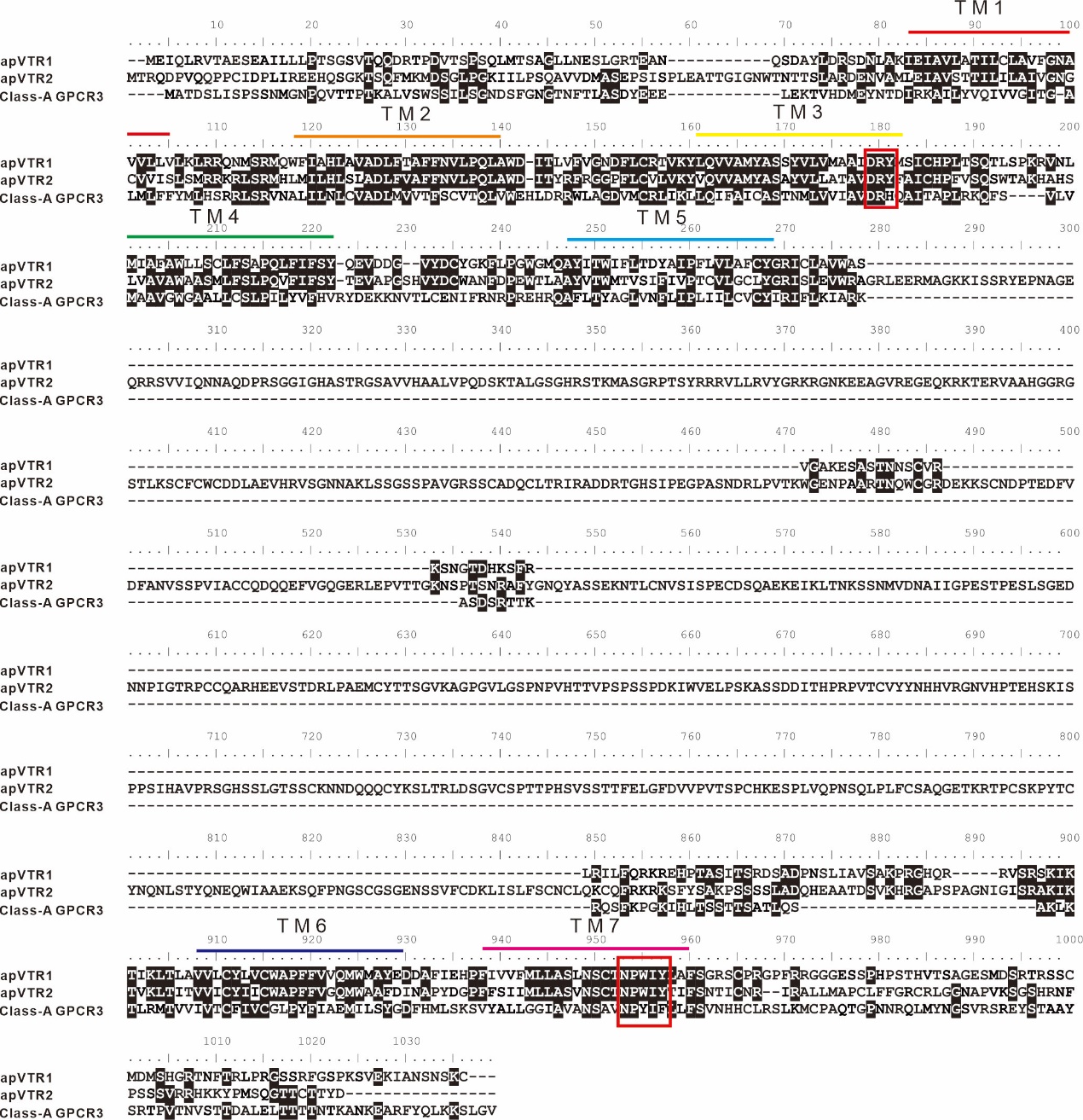


**Supplementary Figure S6.** Alignment of the three putative receptors: apVTR1, apVTR2, and Class-A GPCR3 using BioEdit (ClustalW Multiple alignments - Graphic View). It shows that the amino acids at the 7 TM domains are more conserved than amino acids at other locations. The conserved motifs in TM3 (D/ERY) and TM7 (NPXXY) are marked by red boxes.

**Supplementary Figure S7**


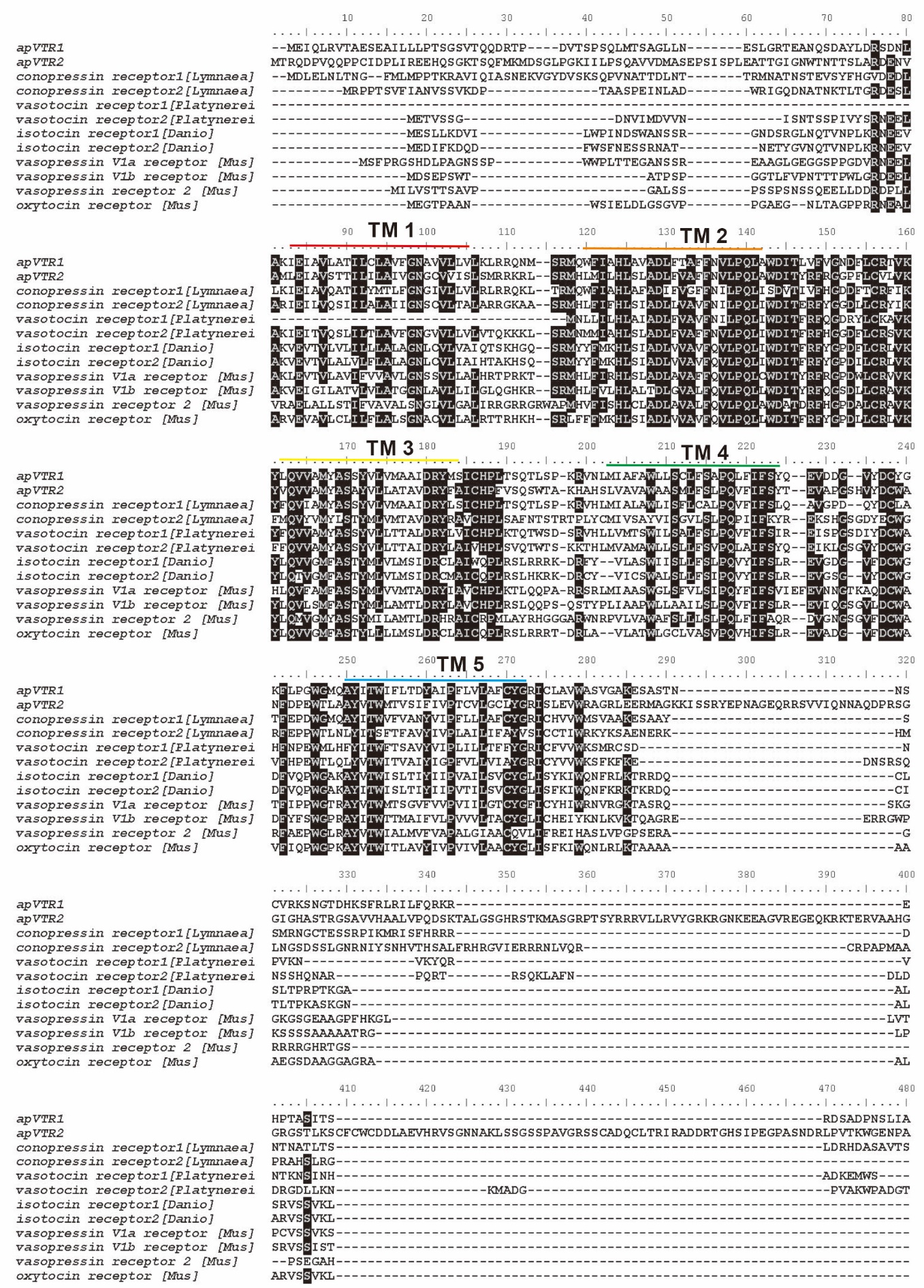


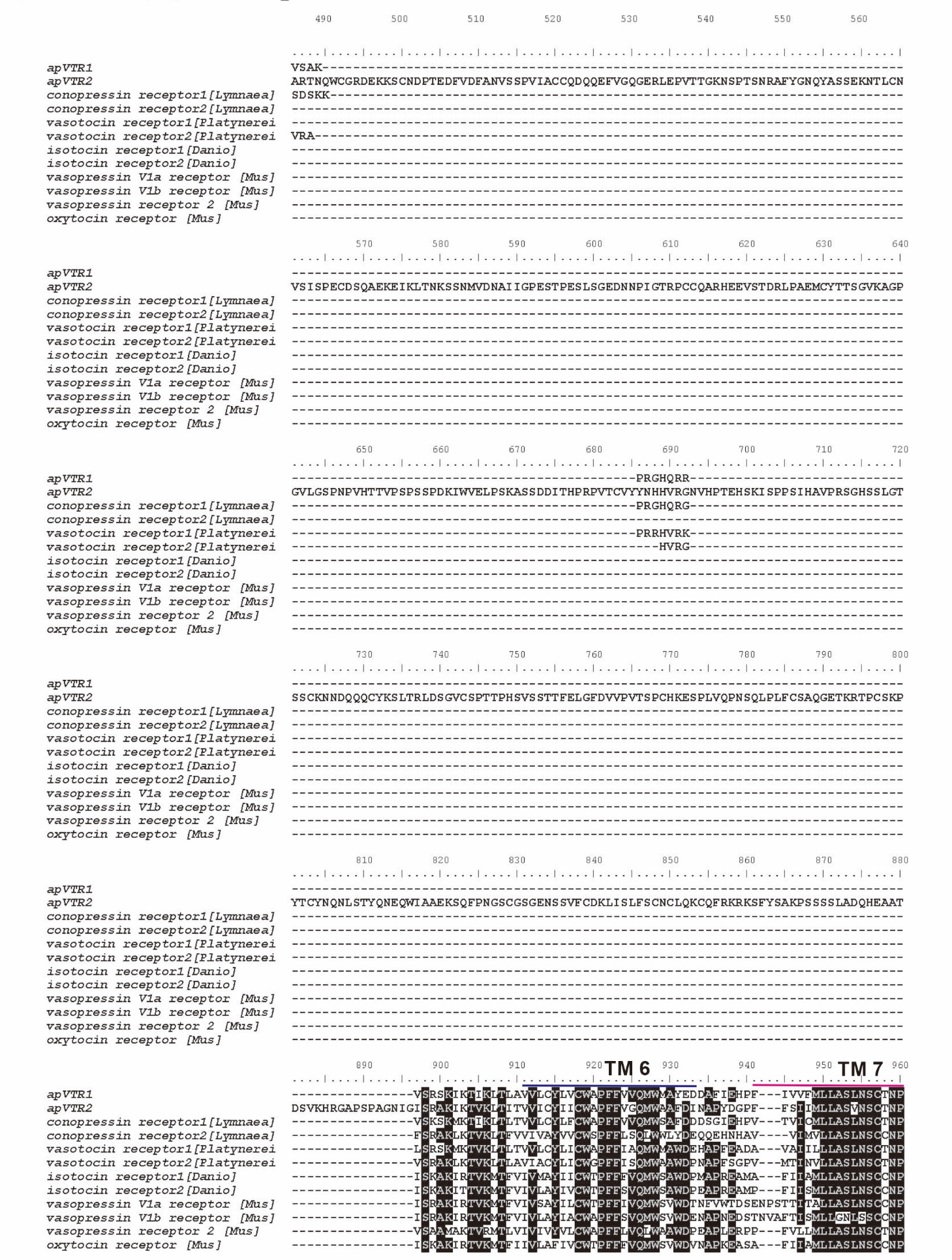


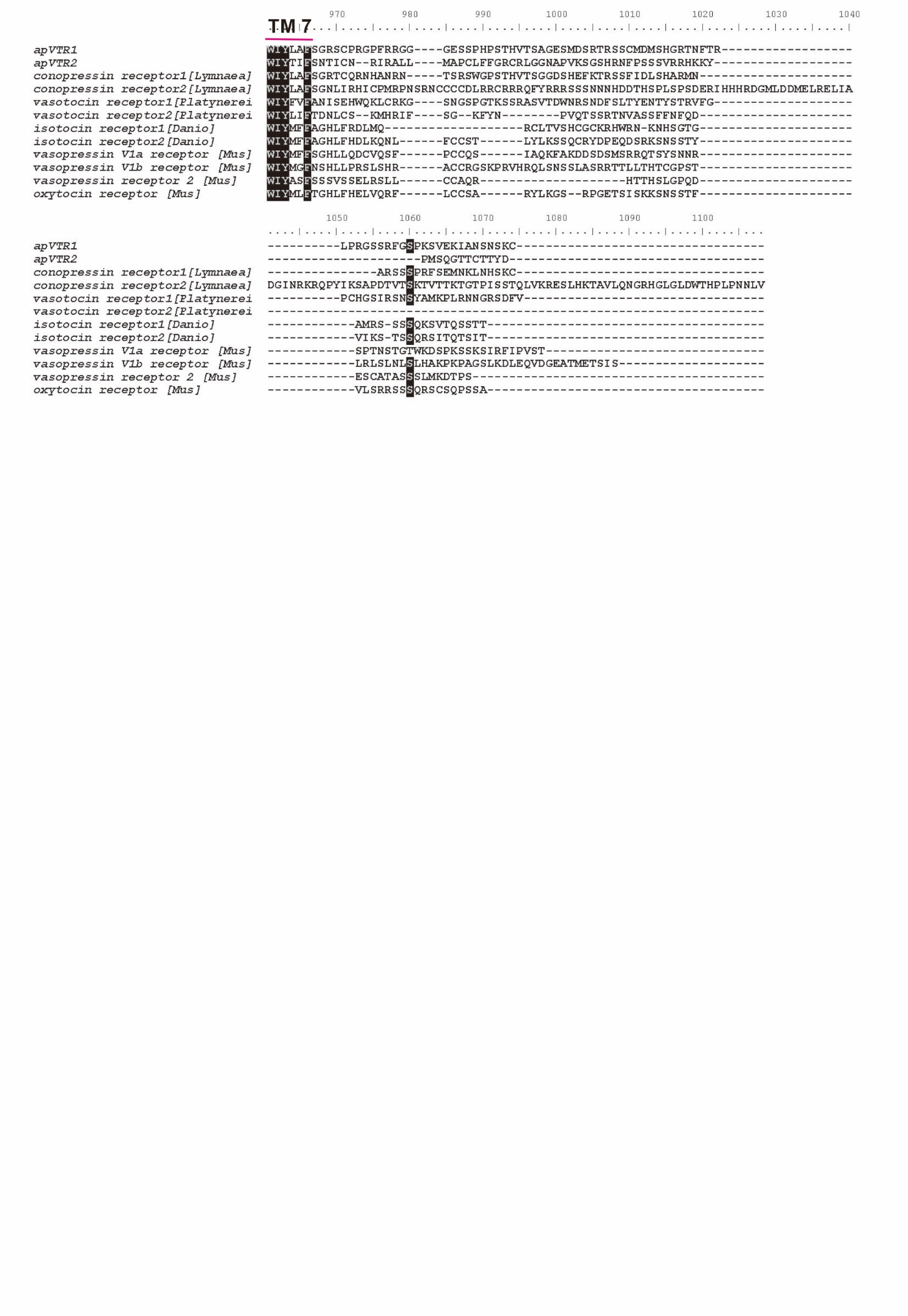


**Supplementary Figure S7.** Multiple alignments between the apVTRs and vasopressin/oxytocin receptors in other species using BioEdit (ClustalW Multiple alignments - Graphic View). It shows that the amino acids at the 7 TM domains are more conserved than amino acids at other locations.

**Supplementary Figure S8**


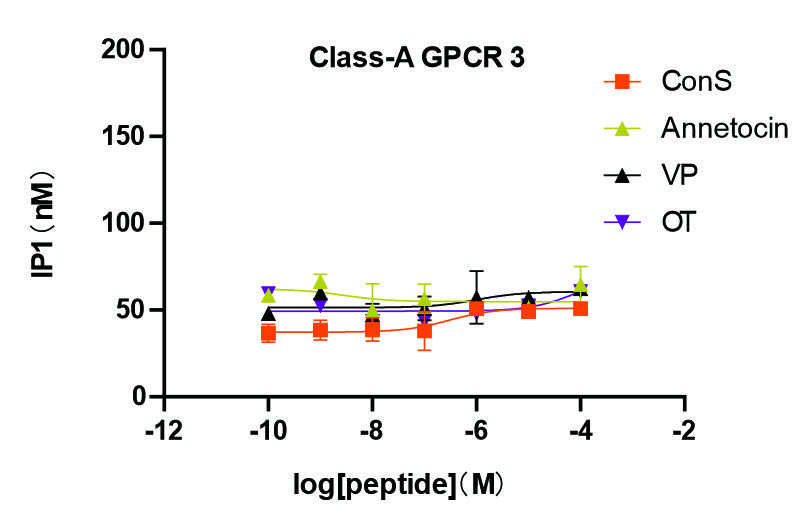


**Supplementary Figure S8.** The effect of vasopressin/oxytocin-like peptides from other species on the Class-A GPCR3. The results show that ConS, Annetocin, Vasopressin (VP), and Oxytocin (OT) (n = 3 for all) do not affect Class-A GPCR 3. In all the experiments with Class-A GPCR3, we have co-transfected the promiscuous Gαq (also known as Gα16).

**Supplementary Figure S9**


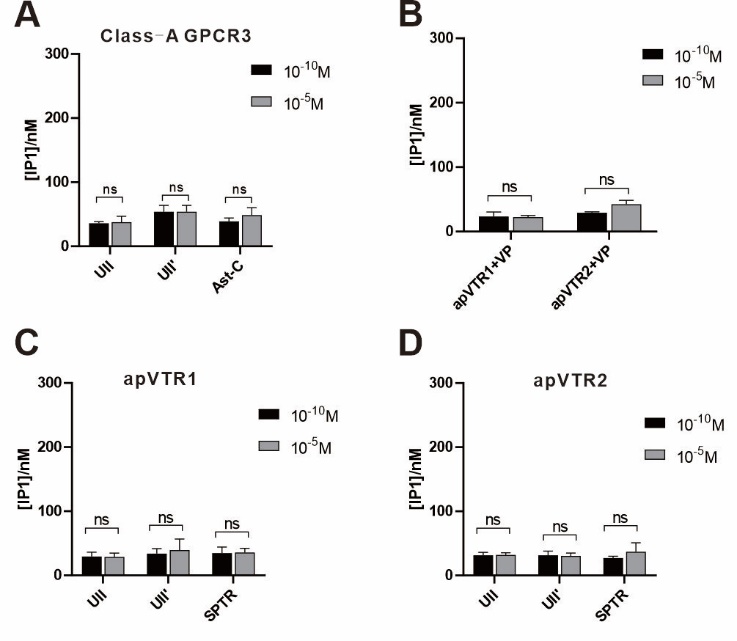


**Supplementary Figure S9.** Effects of other neuropeptides with a disulfide bond on the three putative receptors were determined using an IP1 accumulation assay. **(A)** Effects of the *Aplysia* neuropeptides (urotensin II, urotensin II', Ast-C) on the Class-A GPCR 3. In all the experiments with Class-A GPCR3, we have co-transfected the promiscuous Gαq (also known as Gα16). **(B)** Effects of the human vasopressin (VP) on apVTRs. **(C)** Effects of the other *Aplysia* neuropeptides with a disulfide bond (urotensin II, urotensin II', SPTR) on apVTR1. **(D)** Effects of the other *Aplysia* neuropeptides with a disulfide bond (urotensin II, urotensin II', SPTR) on apVTR2. Paired t-test, ns, not significant; error bar: SEM.

**Supplementary Figure S10**


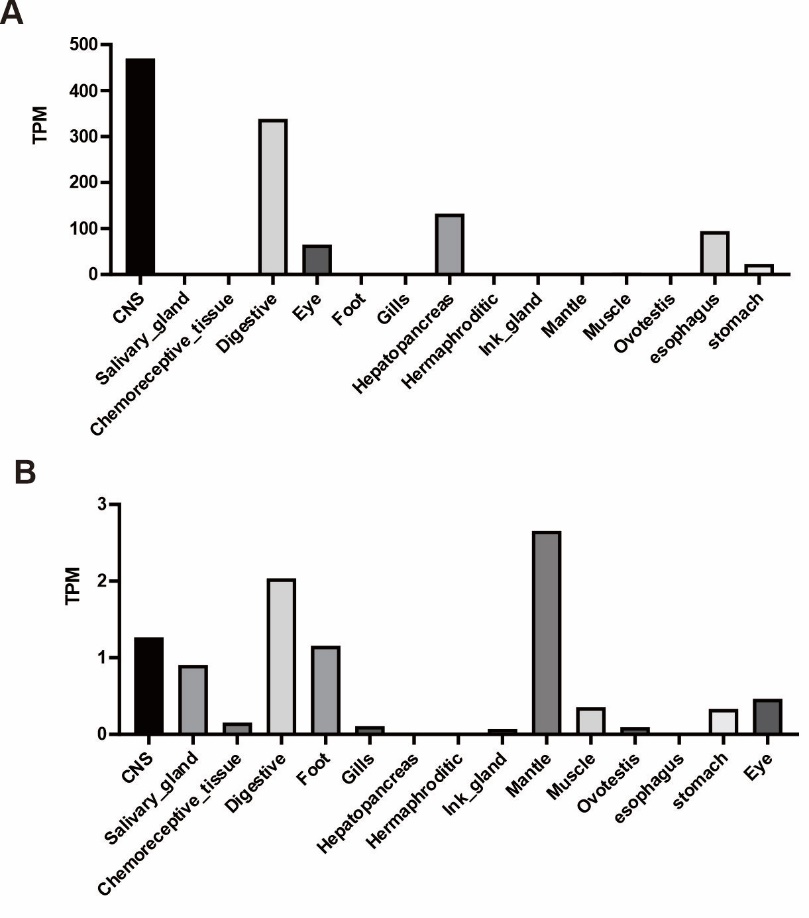


**Supplementary Figure S10.** The apVT precursor and apVTR1 expression levels in multiple tissues of *Aplysia*. **(A)** The apVT precursor (XM_013084328.1) expression levels in multiple tissues. **(B)** The apVTR1 (XM_013088972.2) expression levels in multiple tissues. The data represented as TPM

(Transcript per million) values

1. **Supplementary Tables**

**Supplementary Table S4:** The results that Class-A GPCR 3 blasting in *Mus musculus*, *Danio rerio* and *C. elegans*.

**Supplementary Table S4.1 The blast results in *Mus musculus***

| ***Mus musculus*** |  |  | **Accession** | **Total Score** | **Query Cover** | **E value** | **% Positives** |
| --- | --- | --- | --- | --- | --- | --- | --- |
|  | **apVTR1** | Vasopressin V1b receptor | NP_036054.1 | 276 | 87% | 9.00E-88 | 53.85 |
|  |  | Vasopressin V1b receptor | AAF73221.1 | 275 | 79% | 4.00E-87 | 55.26 |
|  |  | vasopressin V1a receptor | NP_058543.2 | 271 | 77% | 8.00E-86 | 57.6 |
|  |  | vasopressin V1a receptor | BAA08567.1 | 270 | 74% | 2.00E-85 | 57.98% |
|  | **apVTR2** | Vasopressin V1b receptor | AAF73221.1 | 291 | 31% | 6.00E-56 | 63.22 |
|  |  | vasopressin V1a receptor | NP_036054.1 | 291 | 31% | 7.00E-56 | 63.22 |
|  |  | vasopressin V1a receptor | BAA08567.1 | 281 | 29% | 6.00E-54 | 62.77 |
|  |  | vasopressin V1a receptor | NP_058543.2 | 281 | 26% | 7.00E-54 | 65.53 |
|  | **Class-A GPCR 3** | vasopressin V1a receptor | BAA89050.1 | 133 | 85% | 1.00E-33 | 49.26 |
|  |  | vasopressin V1a receptor | BAA08567.1 | 132 | 85% | 2.00E-33 | 49.26 |
|  |  | vasopressin V1a receptor | NP_058543.2 | 132 | 76% | 2.00E-33 | 50.54 |
|  |  | vasopressin V1a receptor | NP_036054.1 | 110 | 86% | 7.00E-26 | 46.93 |

**Supplementary Table S4.2 The blast results in *Danio rerio***

| ***Danio rerio*** |  |  | **Accession** | **Total Score** | **Query Cover** | **E value** | **% Positives** |
| --- | --- | --- | --- | --- | --- | --- | --- |
|  | **apVTR1** | vasopressin receptor 1Ab | NP_001284605.1 | 281 | 74% | 4.00E-90 | 58.5 |
|  |  | oxytocin receptor | NP_001186299.1 | 274 | 76% | 1.00E-87 | 59.5 |
|  |  | vasopressin receptor 1Aa | NP_001288043.1 | 267 | 82% | 5.00E-85 | 54.07 |
|  |  | oxytocin receptor b | NP_001186298.1 | 261 | 74% | 1.00E-82 | 61.25 |
|  | **apVTR2** | vasopressin receptor 1Aa | NP_001288043.1 | 266 | 27% | 2.00E-51 | 64.49 |
|  |  | oxytocin receptor | NP_001186299.1 | 265 | 29% | 1.00E-47 | 62.95 |
|  |  | oxytocin receptor b | NP_001186298.1 | 265 | 29% | 5.00E-46 | 65.55 |
|  | **Class-A GPCR 3** | oxytocin receptor | XP_003198871.1 | 127 | 74% | 2.00E-32 | 52.11 |
|  |  | vasopressin receptor 1Ab | NP_001284605.1 | 124 | 77% | 4.00E-31 | 49.29 |
|  |  | oxytocin receptor | NP_001186299.1 | 118 | 83% | 6.00E-29 | 49.2 |
|  |  | oxytocin receptor b | NP_001186298.1 | 116 | 83% | 2.00E-28 | 49.34 |

| ***C. elegans*** |  |  | **Accession** | **Total Score** | **Query Cover** | **E value** | **% Positives** |
| --- | --- | --- | --- | --- | --- | --- | --- |
|  | **apVTR1** | Nematocin receptor 1 | NP_493193.1 | 136 | 70% | 9.00E-36 | 46.69 |
|  |  | Nematocin receptor 2 | NP_510477.1 | 113 | 87% | 2.00E-27 | 45.09 |
|  | **apVTR2** | Nematocin receptor 1 | NP_493193.1 | 89.4 | 20% | 1.00E-18 | 51.85 |
|  |  | Nematocin receptor 2 | NP_510477.1 | 123 | 25% | 7.00E-13 | 56.88 |
|  | **Class-A GPCR 3** | Nematocin receptor 2 | NP_510477.1 | 94.7 | 65% | 4.00E-21 | 48.05 |
|  |  | Neuropeptide receptor 22 | NP_001023540.1 | 90.9 | 70% | 1.00E-19 | 45.82 |
|  |  | Neuropeptide receptor 22 | NP_001023541.1 | 90.1 | 73% | 2.00E-19 | 45.4 |
|  |  | Nematocin receptor 1 | NP_493193.1 | 70.5 | 68% | 5.00E-13 | 45.25 |

**Supplementary Table S4.3 The blast results in *C. elegans***
